# 7^th^ International Workshop on the Molecular Biology and Genetics of the Lepidoptera: August 20–26, 2006 Orthodox Academy of Crete, Kolympari, Crete, Greece

**DOI:** 10.1673/031.007.2901

**Published:** 2007-05-04

**Authors:** Kostas Iatrou, Pierre Couble

**Affiliations:** Institute of Biology, National Centre for Scientific Research “Demokritos”, Athens, Greece; iatrou@bio.demokritos.gr CNRS, University Claude Bernard, Lyon, France pierre.couble@univ-lyon1.fr

## Dedication

By Marian Goldsmith

We dedicate this year's Lepidoptera Workshop to the memory of Michael Wells, who died in May. Mike's passing was a profound loss to our community. He was a long time supporter and contributor to the Lepidoptera Workshops, going back to its founding days in the late 1980s. His lab created and hosted the meeting's website, giving us wider international exposure and facilitating registration. The research areas that he opened up in insect biochemistry and physiology provided a foundation for many of the projects reported at the meeting over the years. He was always available with constructive advice and support, both scientific and professional, and mentored many junior and senior scientists, not only in the lively discussion sessions at the meeting but also in private conversation. At the opening of this year's Workshop people commented that one of his greatest joys was in educating school children and the general public about science using *Manduca sexta*, his research organism, as a model. To recognize Mike's dedication to the spirit of science and dialogue is especially fitting in the context of the Orthodox Academy of Crete, where we regularly hold the Lepidoptera Workshops.

Abstracts are listed in alphabetical order by the last name of the first author.

