# Supplementary material for: 7th International Workshop on the Molecular Biology and Genetics of the Lepidoptera: August 20–26, 2006: Orthodox Academy of Crete, Kolympari, Crete, Greece
Source: J Insect Sci. 2007 May 4;7:29. doi: 10.1673/031.007.2901 (PMC2999431; doi:10.1673/031.007.2901)
Supplement: Related texts

# Research article: Impact of punctual mutations in the *cap* gene of *Junonia coenia* densovirus (*Jc*DNV) on virus assembly and infectivity to Ld 652 cells and *Spodoptera littoralis* larvae

**DOI:** 10.1673/031.007.2902

**Published:** 2007-05-04

**Authors:** A. Abd-Alla, F-X. Jousset, F. Cousserans, M. Bergoin

**Affiliations:** ^1^ UMR 1231 BIVI, Univ. Montpellier 2, 34095 Montpellier, France,; ^2^ Entomology Unit FAO/IAEA Agriculture and Biotechnology Laboratory, A-2444 Seibersdorf, Austria. bergoin@ensam.inra.fr

The *cap* gene of *Jc*DNV occupies the 5′ half of one strand of its ambisense genome and is under control of the P9 promoter. The 4 structural polypeptides of the virion are translated from a single unspliced 2.6 kb mRNA by translation initiation at the 1st, 2nd, 3rd and 4th AUG codons according to a “leaky scanning” mechanism. We previously reported the production of *Jc*DNV virus-like particles using different recombinant baculoviruses (Croizier *et al*., 2000 J. *Gen. Virol*, 81, 1605–1613). However, the role of each polypetide in virus assembly to produce infectious virions has not been established. We report here the effect of punctual mutations deleting one or more capsid polypeptides on virus production in cell culture and larvae. Six constructs were generated from pBRJ, a plasmid encompassing an infectious viral sequence, by site-directed mutagenesis of the 5 in-frame ATG's at positions 555 (pJm1), 1386 (pJm2), 1521 (pJm3), 1668 (pJm4), and 1674 (pJm5) of the *cap* ORF. Other plasmids pJm2+3 and pJm4+5 contained the double ATG2+ATG3 and ATG4+ATG5 mutations, respectively while pJmss6-7 contained a double TAA mutation upstream of ATG4. With the exception of pJm4+5, all these constructs generated mutant virions (VJm) when transfected to Ld 652 cells. However, infectivity tests performed by injecting virions produced in Ld 652 cells to 3rd instar *Spodoptera littoralis* larvae revealed significant differences among them: VJm2, VJms, VJm4 and VJm5 virions lacking VP2 and VPs respectively but containing VP4 appeared to be as infectious as *wt* virions. In contrast, Vjm1, VJm2+3, and VJm336-7 virions lacking VP1, VP2+VP3, VP1+VP2 +VP3 in their capsid respectively, were not infectious for *S.littoralis* larvae. Finally, mutations were performed in two regions assumed to be critical: the N-terminal “VP1up” sequence containing a phospholipase A2 activity and a Lysine-Arginine-reach region close to the N-terminal sequence of VP2. Mutation of the PLA2 active site drastically reduced infectivity of mutant virions while mutation in VP2 N-terminal region completely abolished infectivity. As demonstrated by dot-blot hybridization and PCR, all the mutant virions contained DNA. These results will be discussed in the light of confocal observations of Ld 652 cells infected with different mutants.

# Research article: Identification of the female-determining region of the *Bombyx mori* W chromosome using W chromosome variants

**DOI:** 10.1673/031.007.2903

**Published:** 2007-05-04

**Authors:** H. Abe, T. Fujii, K. Mita, M. Ajimura, T. Shimada

**Affiliations:** ^1^ Department of Biological Production, Faculty of Agriculture, Tokyo University of Agriculture and Technology, Tokyo 183–8509, Japan; ^2^ Department of Agricultural and Environmental Biology, Graduate school of Agricultual and Life Sciences, The University of Tokyo, Tokyo 113–8657, Japan; ^3^ Laboratory of Insect Genome, National Institute of Agrobiological Science, Tsukuba 305–8634, Japan, kmita@nias.affrc.go.jp

In the silkworm *Bombyx mori* (female, ZW; male, ZZ), femaleness is determined by the presence of a single W chromosome, irrespective of the number of autosomes or Z chromosomes. The W chromosome is devoid of functional genes, except the putative female-determining gene (*Fem*). However, there are strains in which the autosomal fragment containing the dominant marker genes were translocated onto the W chromosome. In this study, we analyzed the W chromosomal regions of the two sex-limited yellow cocoon strains (T(W;2)*Y*-Abe and -Chu types) and the newly obtained chromosomal fragment from the Zebra-W strain (T(W;3)*Ze* chromosome) at the molecular level. Initially, we obtained the W chromosome variant from the T(W;3)*Ze* chromosome by X-ray irradiation and obtained one zebra male larva having the chromosome fragment of T(W;3)*Ze* (W(B-YL-YS)*Ze* chromosome). The normal W chromosomes of the strains in Japan contained the 12 RAPD markers (W-Kabuki, W-Kamikaze, W-Sasuke, W-Musashi, W-Sakura, BMC1-Kabuki, W-Rikishi, W-Yukemuri-L, W-Yukemuri-S, W-Bomsai, W-Samurai and W-Mikan), the W(B-YL-YS)*Ze* chromosome contained three (W-Yukemuri-L, W-Yukemuri-S and W-Bonsai), the T(W;2)*Y*-Chu chromosome contained six (W-Rikishi, W-Yukemuri-L, W-Yukemuri-S, W-Bonsai, W-Samurai and W-Mikan), and the T(W;2)*Y*-Abe chromosome contained only one (W-Rikishi). Using prepared sucking stomachs of the p50 strain having the normal W chromosome and the sex-limited yellow cocoon strain (T(W;2)*Y*-Abe type), a single sex heterochromatin body was detected in nuclei of the p50 female moth, while no sex heterochromatin body was detected in the sex-limited yellow cocoon strain (T(W;2)*Y*-Abe). Therefore, we conclude that the regions of the W chromosome of the T(W;2)*Y*-Abe containing 11 of the 12 W-specific RAPD markers were deleted, leaving the extremely limited region containing W-Rikishi RAPD marker, which is sufficient to determine femaleness.

# Research article: Are the zinc-finger motif genes, z1 and z20, located in the W chromosome involved in the sex-determination of the domesticated silkworm, *Bombyx* mori?

**DOI:** 10.1673/031.007.2904

**Published:** 2007-05-04

**Authors:** M. Ajimura, K. Sahara, H. Abe, T. Tamura, T. Shimada, K. Mita

**Affiliations:** ^1^ Insect Genome Research Unit, Nat'l Institute of Agrobiol. Sciences, Tsukuba, 305–8634, Japan; ^2^ Div. of Applied Bioscience, Grad. School of Agric., Hokkaido University. Sapporo, 060–8589, Japan; ^3^ Faculty of Agric., Tokyo University of Agric. and Tech., Tokyo, 183–8509, Japan; ^4^ Transgenic Silkworm Res. Center, Nat'l Institute of Agrobiol. Sciences, Tsukuba, 305–8634, Japan; ^5^ Dept. of Agric. and Environ. Biology, University of Tokyo, Tokyo 113–8657, Japan. majimu@nias.affrc.go.jp, kmita@nias.affrc.go.jp

The silkworm (*Bombyx mori*) has 27 pairs of autosomes and 2 sex-chromosomes (Z and W). A putative sex-determining gene (*Fem*) is located on the W chromosome and autonomously determines the femaleness in each cell during embryogenesis. According to the previous studies on the genome structure of W, most part of the W chromosome is heterochromatic region consisting of different types of transposons and very little euchromatic region has been isolated so far. Furthermore, it is difficult to construct a genetic map of the W chromosome because no recombination is carried out in female meiosis. For such reasons the isolation of *Fem* has not been succeeded yet. If *Fem* located in the W chromosome, one unique sequence must be present as the *Fem* gene at least. Based on the hypothesis, we carried out a cloning of *Fem* by a genomic subtraction method (RDA). By the extensive screening, a 380 bp DNA fragment (18G08) showing W-specific characteristics was obtained. We isolated W-BACs covering the region and analyzed the whole sequences. The sequencing of the BACs showed that the region contains an euchromatic region expanding about 50 kbp and 250 kbp of transposon-rich part. The region includes two genes (*z1* and *Z20*) having zinc-finger motifs, C3H1- and C2H2-types. Further analysis of the genes revealed that the *Bombyx* genome has 6 copies of *z1* and *Z20* homologs; 2 copies in the chromosome XXV and 4 copies dispersed along the W chromosome. In each case, both *z1* and *Z20* homologs are present as a pair mostly in tail-to-tail orientation. Sequence comparison of these repeats revealed the four *z1*-*z20* copies in the W chromosome were generated from a autosomal gene by repeated duplications. Results of PCR and FISH experiments with several W deletion/traslocation chromosomes indicated *z1*-*z20* could be mapped within a putative *Fem* gene (about 10 % of the W chromosome). An analysis of expression pattern of the *z1* and *Z20* genes during embryogenesis showed that no transcripts of *z1* and *Z20* was detected at an early stage of embryonic development. The transcription was observed before sex-determination (at the first to second days after fertilization). On the other hand, transcriptions of the autosomal zinc-finger motif genes are observed constitutively through embryogenesis. Interestingly, we found that initiation site of the mRNA for both *z1* and *Z20* genes in the W were located within transposons which were present outside of euchromatic region. This suggests that the transcription of the *z1* and *Z20* are caused either by transposon sequence or by W-specific structure at hetero-euchromation boundary. These observations suggest both *z1* and *Z20* genes in the W are candidates for *Fem* gene. We have constructed transgenic silkworm for *z1* and *Z20* genes and are currently analyzing their phenotypes whether these genes are involved in sex-determination process. Dr. Nagaraju's group also isolated the identical gene (*z1*) independently by differential display and will talk on their results in this session.

# Research article: The PK/PBAN family of insect neuropeptides: mode of action and a target for the design of novel insect control agents

**DOI:** 10.1673/031.007.2905

**Published:** 2007-05-04

**Authors:** M. Altstein, A. Hariton, M. Davidovitch, O. Ben-Aziz

**Affiliations:** Department of Entomology, The Volcani Center, ARO, Bet Dagan, 50250, Israel. vinnie2@agri.gov.il

The PK/PBAN family is a multifunctional family of peptides that plays a major role in the physiology of insects. The family comprises of peptides such as PBAN, melanization and reddish coloration hormone (MRCH), pyrokinins, myotropins, pheromonotropin (Pss-PT) and diapause hormone (DH) - which have been found to control a variety of functions such as: stimulation of sex pheromone biosynthesis in adult female moths, cuticular melanization in moth larvae, contraction of the locust oviduct, myotropic activity of the cockroach and locust guts, egg diapause in the silkworm, and acceleration of pupariation in flesh-fly larvae. Despite the intensive studies of the bioactivity of this family, very little is known about the molecular and cellular basis that underlies the functional diversity of the PK/PBAN family. In the past few years our studies focused on a detailed characterization of the structure activity relationship (SAR) of the PK/PBAN family, on characterization of the PK/PBAN receptor and on the development of a novel strategy for the generation of PK/PBAN antagonists and their employment in studying the mode of action of the PK/PBAN peptides. Our results on those topics and their implementation for the development of a novel approach for rational design of insecticide prototypes will be presented.

# Research article: *Spodoptera frugiperda* transcription profiling in response to injection of the polydnavirus associated with the wasp *Hyposoter didymator*

**DOI:** 10.1673/031.007.2906

**Published:** 2007-05-04

**Authors:** M. Barat-Houari, F. Hilliou, F.-X. Jousset, L. Sofer, E. Deleury, J. Rocher, M. Ravallec, L. Galibert, R. Feyereisen, P. Fournier, A-N. Volkoff

**Affiliations:** ^1^ UMR 1231 Biologie Intégrative et Virologie des Insectes. INRA - Université de Montpellier II. Place Eugène Bataillon, Case Courrier 101, 34095 Montpellier Cédex, France; ^2^ UMR 1112 R.O.S.E. INRA - Université de Nice-Sophia Antipolis, Laboratoire de Génomique Fonctionnelle des Insectes, 400 route des Chappes, BP 167, 06 903 Sophia Antipolis cedex, France. volkoff@ensam.inra.fr

To succeed in their development, parasites must render their host suitable and circumvent the host immune defence. One strategy developed by some endoparasitic wasps of the ichneumonoid family is the association with symbiotic prokaryotes of the polydnavirus family. Polydnaviruses are maintained as proviruses integrated in the wasp genome. Virus particles that contain the typical polydnaviral segmented genome are produced in the wasp ovaries and then injected by the female wasp in the lepidopteran host during oviposition. In the lepidopteran host, infection of several tissues by polydnaviruses results in physiological alterations, including immune suppression, which are required for pre-imaginal development of the wasp. In order to improve our knowledge on host-parasite interactions involving polydnaviruses, we have developed a transcriptomic approach that has enabled us to start analysing the interactions between the parasitoid *Hyposoter didymator*, an ichneumonid wasp associated with the polydnavirus HdIV, and its lepidopteran host *Spodoptera frugiperda*. A microarray with 1751 cDNAs was used to analyse the gene expression profile of *S. frugiperda* hemocytes and fat bodies collected from last instar larvae, 24 hours after injection of filter-purified HdIV. Our results showed that approximately 6% of the 1751 arrayed host genes varied significantly in response to HdIV injection, indicating that factors associated with a parasitic wasp do affect the host transcriptome. HdIV injection mainly causes down-regulation of cellular genes (76% of the genes that vary are down-regulated). Interestingly, several of these genes have been previously shown to be involved in Lepidoptera innate immunity. For example, levels of transcripts related to calreticulin (-2.5), prophenoloxidase-activating enzyme (-2.2), immulectin-2 (-2.0), actin-binding proteins (-1.8 to -2.2) and a novel lepidopteran scavenger receptor (-3.0) are decreased in hemocytes of injected caterpillars. Thus different components of the host immune response (non-self recognition, humoral and cellular responses) seem to be affected and modulated 24 hours after HdIV injection. Conversely, we found an increased rate of transcription for a galactose-binding lectin (+5.5), a c-type lectin (+1.7) similar to a molecule encoded by bracoviruses, and surprisingly, for the prophenoloxidase subunits (+4.9 and +5.9). The role of these genes in *S. frugiperda* physiology and/or the reason their rate of transcription vary in response to HdIV injection will need to be further investigated. This novel microarray approach will allow identifying new targets for the virulence factors derived from parasitic wasps and their associated polydnaviruses and will certainly provide new insights on the strategies employed by parasites to manipulate their host physiology.

# Research article: Identifying DNA markers close to quantitative traits in lepidopteran genomes: using wing colour variation in *Heliconius* butterflies as a model

**DOI:** 10.1673/031.007.2907

**Published:** 2007-05-04

**Authors:** Simon W. Baxter, Nicola Chamberlain, Riccardo Papa, Sean J. Humphray, Richard H. ffrench-Constant, W. Owen McMillan, Chris D. Jiggins

**Affiliations:** ^1^ Institute of Evolutionary Biology, School of Biological Sciences, University of Edinburgh, West Mains Road, EH9 3JT, Edinburgh, U. K.; ^2^ Department of Biology and Biochemistry, University of Bath, South Building, Claverton Down, BA2 7AY, Bath, U. K.; ^3^ Department of Biology, University of Puerto Rico, P.O. Box 23360, San Juan, PR 00931; ^4^ The Wellcome Trust Sanger Institute, Wellcome Trust Genome Campus, Hixton, CB10 1SA, Cambridge, U. K. sbaxter@staffmail.ed.ac.uk

Identifying a gene responsible for a trait of interest within Lepidoptera is a multi-step process. Commonly, this involves performing crosses to enable trait segregation, using progeny to create linkage maps, identifying the genome region responsible for variation and then genetic mapping of candidate genes to test for associations. If candidate genes tested do not map to the chromosome or locus of interest, sequencing genomic BAC clones spanning the genome region is required. Here we present a molecular method for identifying DNA markers tightly linked to a quantitative trait, which can be used to screen BAC libraries, using wing colour variation in *Heliconius* butterflies as a model. *Heliconius melpomene* and *H. erato* are co-mimics that share the same warningly coloured wing patterns where they cohabit throughout Central and South America. Approximately 30 races of wing colour variants have been characterised and identifying the colour determining genes will be of value to developmental and evolutionary biology, and population genetics. We have used AFLP methods to identify the genetic locus of a strong visual trait in these sympatric species - red wing patches. Two dominant loci on the same chromosome of *H. melpomene* have previously been found to control red wing colouration: “B” is a large red spot on the forewing and “Dennis” (D) includes ray patterns on the hind wing and red colour on the proximal forewing. Crosses were performed between B/D heterozygotes (BbDd × BbDd) then AFLPs were performed on two progeny bulks; i) homozygotes lacking the B spot (bbD-) or ii) lacking the Dennis pattern (B-dd). When AFLP analysis was performed, it was expected that both bulks would share identical banding patterns, except in areas tightly linked to the B or D loci. AFLP analysis was performed using fluorescently labelled primers on a 3730 DNA Analyzer (Applied Biosystems). From approximately 300 primer combinations, nine bands were identified and confirmed linked the B or D locus. AFLPs were sequenced and used to create genotyping assays for additional broods, which found B and D to be tightly linked. Multiple of the AFLP markers were within 1 centimorgan (∼180kb) of this region, and were used as probes to screen a *H. melpomene* BAC library. A similar strategy has been used to identify an AFLP band close to the Dry locus in *H. erato*, which controls the same phenotype as B and D in *H. melpomene*. Mapping a gene close to the *H. erato* AFLP in *H. melpomene*, has shown homologous genetic regions are responsible for similar red wing phenotypes in these two species. Performing AFLP analysis on bulks of progeny, selected for the presence or absence of a quantitative trait, could be applied to other lepidopteran species.

# Research article: Two species into one: Bottleneck history of *Helicoverpa zea* from *Helicoverpa armigera* revealed by DNA barcoding

**DOI:** 10.1673/031.007.2908

**Published:** 2007-05-04

**Authors:** G.T. Behere, D. Russell, P. Batterham, W. T. Tay

**Affiliations:** ARC Special Research Centre for Environmental Stress and Adaptation Research (CESAR), Department of Genetics, Bio21 Institute of Molecular Science and Biotechnology, The University of Melbourne, Victoria 3010, Australia. p.batterham@unimelb.edu.au

The use of mitochondrial DNA Cytochrome Oxidase I (mtDNA COI) gene in species identification has gained popularity in recent years, with its effectiveness in identifying cryptic and new species demonstrated in birds and Lepidoptera. The noctuid moths of the genus *Helicoverpa* include two of the most devastating agricultural pest species: *H. armigera* of the old world, and *H. zea* found exclusively in the north and south American continents. Both of these species are polyphagous targeting >150 crop species. Phylogenies of *H. armigera* and *H. zea* have to-date been constructed based on coding nuclear DNA sequences and morphological characters, but a mtDNA phylogeny of *H. zea* and *H. armigera* has been lacking. Differentiating *H. zea* and *H. armigera* based on morphological characters relies almost exclusively on characters of the male genitalia, although accurate identification has remained problematic due to over-lapping ranges in character measurements. *H. zea* and *H. armigera* are presently recognised as two separate species despite successful bi-directional, ‘Inter-specific’, mating experiments that gave rise to viable offspring, and the trapping of *H. zea* males when using *H. armigera* sex pheromones in the North American continent. Using a 511 base pair sequence of a partial mtDNA COI gene, we analysed the phylogenetic relationships amongst 228 *H. armigera* individuals sampled from China, Australia, Africa, India and Pakistan, plus 14 *H. zea* from North America, *H. punctigera* from Australia and *H. assulta* from India, using *Heliothis virescens* as an outgroup. Our mtDNA COI phylogeny of *Helicoverpa* species indicates that *H. punctigera* is ancestral to *H. assulta* which is in turn ancestral to *H. armigera* and *H. zea*. Furthermore, the long branch-length of *H. zea* from the *H. armigera* clade suggests a recent bottleneck event in *H. zea*'s separation from *H. armigera. H. zea* and *H. armigera* show an intermediate level of nucleotide diversity, lying between expected values for intra-specific and inter-specific sequence comparisons, possibly suggesting rapid nucleotide divergence in *H. zea* due to selection pressures imposed on movement by agricultural practices.

# Research article: Developing genomic resources for a butterfly evo-devo model

**DOI:** 10.1673/031.007.2909

**Published:** 2007-05-04

**Authors:** P. Beldade, S. Rudd, J.D. Gruber, A.D. Long

**Affiliations:** ^1^ Department of Ecology and Evolutionary Biology, University of California at Irvine, USA; ^2^ Institute of Biology of the University of Leiden; The Netherlands; ^3^ Turku Centre for Biotechnology, Turku, Finland. pbeldade@biology.leidenuniv.nl

*Bicyclus anynana* (Nymphalidae, Satyrinae) has been established as a laboratory organism in the study of the evolution and development of wing patterns. I will describe an Expression Sequence Tag (EST) project for *B. anynana* that has identified the largest publicly available collection to date of expressed genes for any butterfly. By targeting cDNAs from developing wings at those stages when pattern is specified, we biased gene discovery towards genes potentially involved in pattern formation. Assembly of 9,903 ESTs from a subtracted library allowed us to identify 4,251 genes of which 2,461 were annotated based on blast analyses against relevant gene collections. Gene prediction software identified 2,202 peptides, of which 215 longer than 100 amino acids had no homology to any known proteins and thus potentially represent novel or highly diverged butterfly genes. We combined gene and Single Nucleotide Polymorphism (SNPs) identification by constructing cDNA libraries from pools of outbred individuals, and by sequencing clones from the 3′ end to maximize alignment depth. Alignments of multi-member contigs allowed us to identify over 14,000 SNPs, with 316 genes having at least one high confidence double-hit SNP. We furthermore identified 320 microsatellites in transcribed genes that could potentially be used as markers. These resources are being used to develop genomic tools for *B. anynana* and will be invaluable for exploring the potential of this and butterflies in as models in ecological, evolutionary, and developmental genetics.

# Research article: Cloning, recombinant production and activity of pacifastin-like peptides

**DOI:** 10.1673/031.007.2910

**Published:** 2007-05-04

**Authors:** B. Breugelmans, G. Simonet, S. Van de Velde, S. Van Soest, G. Smagghe, J. Vanden Broeck

**Affiliations:** ^1^ Laboratory of Developmental Physiology, Genomics and Proteomics, Department of Animal Physiology and Neurobiology, Zoological Institute, K.U. Leuven, Naamsestraat 59, B-3000 Leuven, Belgium.; ^2^ Laboratory of Agrozoology, Department of Crop Protection, Faculty of Agricultural and Applied Biological Sciences, Ghent University, Coupure Links 653, B-9000 Ghent, Belgium. Bert. Breugelmans@bio.kuleuven.be

Pacifastins are canonical serine protease inhibitors which appear to be restricted to Arthropods. Members of this family were originally purified from a Crustacean and have been identified in two Orthopteran locust species and in a Hymenopteran wasp. *In silico* data mining predicted the existence of more pacifastin-like members in several other insects such as the malaria mosquito (*Anopheles gambiae*) and the silkworm (*Bombyx mori*). Every member of this family is derived from a precursor protein. Except for one, all pacifastin precursors contain multiple inhibitor domains, which are usually separated from each other by dibasic cleaving sites. Using the available genome and EST databases, we have cloned the first lepidopteran pacifastin precursor from fat body of the silkworm (*Bombyx mori*). The precursor, named BMPP-1, codes for 13 pacifastin-like inhibitory domains and constitutes by far the largest pacifastin-like precursor in Arthropods. This brings the total of pacifastin-related peptides up to 45. Although numerous pacifastin-like peptides have been identified in several insect orders, only for a limited number of locust peptides the inhibitory activity towards serine proteases has been analysed. As a consequence, for the majority of the pacifastin-related peptides the (inhibitory) activity remains as yet to be studied. Therefore, in order to further unveil the biochemical and functional properties of this inhibitory family, we have optimised the recombinant production of both locust and *Bombyx* pacifastin-related inhibitors. This allowed us to test the inhibiting capacities of the recombinant peptides towards mammalian proteases, as well as towards endogenous proteases in crude tissue extracts (haemolymph, fat body and gut) of *Spodoptera littoralis, Bombyx mori, Schistocerca gregaria* and *Locusta migratoria*. Preliminary results of these *in vitro* tests show that (i) in addition to bovine (chymo)trypsin insect gut and hemolymph proteases are inhibited, (ii) not all inhibitors have the same potency, (iii) most inhibitors have a specificity for either trypsin- or chymotrypsin-like proteases, conforming to the P1-residue and (iv) there seems to be species selectivity but only for trypsin-like pacifastin inhibitors. Furthermore, the effect of recombinant peptides on a cell line of lepidopteran midgut cells has been analysed. Initial results show that one particular locust peptide has a distinct inhibitory effect on cell proliferation.

# Research article: The molecular genetics of the H locus: colour polymorphism determination in *Papilio dardanus*

**DOI:** 10.1673/031.007.2911

**Published:** 2007-05-04

**Authors:** R. Clark, S. Brown, D. Heckel, C. D. Jiggins, S. Collins, A. P Vogler

**Affiliations:** ^1^ Entomology Department, The Natural History Museum, SW7 5BD, London, UK and Department of Life Science, Imperial College, SW7 2AZ, London, UK.; ^2^ Dept. Genetics, University of Melbourne, Parkville 3010, Victoria, Australia.; ^3^ Max Planck Institute of Chemical Ecology, D-07745 Jena, Germany; ^4^ Institute of Evolutionary Biology, School of Biological Sciences, University of Edinburgh, EH9 3JT, Edinburgh, UK.; ^5^ African Butterfly Research Institute (ABRI), Nairobi, Kenya. rebecca.clark@ic.ac.uk

*Papilio dardanus* has been an iconic example of selection at work in the classical evolutionary and genetics literature due to the extensive polymorphism and Batesian mimicry found in the females of this species. This phenotypic diversity is controlled by a single autosomal genetic locus, termed H, of which ten distinct alleles have so far been identified through Mendelian crossing experiments. Despite extensive debate as to the nature of H (using supergene, single regulatory gene or combinatorial hypotheses) the locus has remained enigmatic. As a single locus within a single species (and therefore expressed on a relatively uniform genetic background) controlling extensive phenotypic diversity (some of which has known adaptive value) the H locus system is ideal for evolutionary developmental studies. The current assumption that major developmental genes identified in model organisms as acting during patterning through all stages of development also specify the pattern on the wing can be addressed through the identification of H and the mechanism by which alleles at this locus affect the phenotype across the wing. Molecular genetics approaches to the identification of H have resulted in the delimitation of the H genome region, through the production of flanking AFLP markers, and the mapping of a major regulatory gene to this region. This candidate gene co-segregates with the H phenotype in all female offspring (from crosses involving several colour morphs) including those known to be recombinants between the AFLP markers and H. With the identification of such a strong candidate for H population level studies are being used to strengthen the evidence supporting the association between the genotype at the candidate locus and the H phenotype.

# Research article: Dissecting butterfly wing pattern formation in Batesian and Mullerian mimicry

**DOI:** 10.1673/031.007.2912

**Published:** 2007-05-04

**Authors:** N. Chamberlain, S. Baxter, C. Jiggins, R.H. ffrench-Constant

**Affiliations:** ^1^ Center for Evolutionary and Conservation Biology, Tremough, University of Exeter, UK; ^2^ Institute of Evolutionary Biology, University of Edinburgh, UK. nc230@bath.ac.uk

We are studying two species as models for both Batesian mimicry and Mullerian mimicry. In the Eastern Tiger swallowtail of North America, which is a Batesian mimic of the Pipevine swallowtail, we are studying the biochemical basis of melanism in the melanic female mimics. We have defined the biochemical reactions that lead to the abnormal melanism of the female and highlighted the role of both dopa-decarboxylase (DDC) and BAS (the homolog of the fly gene ebony). The role of the rate of scale development in this phenomenon will also be discussed. More recently, we have been working with C. Jiggins on Mullerian mimicry in *Heliconius melpomene*. In this species, we are carrying out a chromosomal walk to clone the gene, *Yb*, which is responsible for the yellow band on the hind wing. We will describe progress in the BAC walk and look at any candidate genes in the region. The *Yb* locus is interesting as it seems to represent a ‘super-gene’ controlling all of the pattern in a different species *H. numata* (M. Joron, submitted).

# Research article: Independent modulation of multiple genes using modified ecdysone receptors

**DOI:** 10.1673/031.007.2913

**Published:** 2007-05-04

**Authors:** O. Chortyk, J. Friz, C. Thompson, P. Kumar, C. Tice, B. Vertin, R. Palli, M. Kumar, A. Meyer, T. Meteyer, H. Smith, D. Cress, B. Li, R. Hormann

**Affiliations:** RheoGene, Inc., 2650 Eisenhower Ave., Norristown, Pennsylvania, 19403, U.S.A.

Drug-inducible regulation of multiple genes independently, simultaneously, and silently with respect to host metabolism is a highly desirable capability in gene therapy and tissue engineering. Insect-derived ecdysone receptor (EcR)-based gene switches are an intriguing option due to anticipated fundamental orthogonality to transcription events related to mammalian homeostasis. The essential components of a highly orthogonal EcR dual channel gene switch and even a potential three-channel gene switch using ligands representing two and three different chemotypes, respectively, have been previously described (Kumar MB et al. 2002. Proceedings of the National Academy of Sciences USA 99:14710–14715; Mohan BK et al. 2004. Journal of Biological Chemistry, in press) Orthogonal actuation of multiple genes with ligands selected from a common chemotype, rather than several unrelated chemotypes, could provide advantages in simplifying pharmacokinetics and pharmacodynamics, and could also potentially ameliorate possible undesired pleiotropic effects. Toward the general goal of orthogonal multiplex gene-switch systems and the specific goal of systems using a single chemotype, a designed library of 246 ligands, including diacylhydrazines and ecdysteroids, was screened against a panel of 68 wild-type and LBD-mutant EcRs in a transcriptional activation assay. EcRs were fused to the GAL4 DNA binding domain in conjunction with a chimeric RXR fused to a VP16 activation domain. Luciferase was employed as a reporter gene, and the vectors were transfected into 3T3 cells. EC50 values for each receptor-ligand pair were obtained and multiple two-, three- and four-channel orthogonalities were observed. We report the influence of chemotype, ligand library subpopulation, and design principles of both mutants and ligands in their contribution to the number and robustness of the resultant orthogonal switch systems. Diacylhydrazine intra-chemotype specificity of selected duplex switches is described using CoMFA methodology employing designed training sets and diversity-selected test sets. Receptor-independent QSAR is considered in view of a diacylhydrazine-docked EcR homology model.

# Research article: Stable Transformation and RNAi in *Helicoverpa armigera*

**DOI:** 10.1673/031.007.2914

**Published:** 2007-05-04

**Authors:** Derek Collinge, Karl Gordon, Carolyn Behm, Steve Whyard

**Affiliations:** ^1^ CSIRO Entomology Canberra, Australia; ^2^ The Australian National University Canberra, Australia; ^3^ University of Manitoba, Canada. derek.collinge@csiro.au

Stable transformation is an essential tool for molecular biologists working on non-model organisms. The ability to introduce and express genes of choice in an organism provides a means to investigate important molecular questions such as gene function, biochemical pathway analysis, reporter gene studies and developmental processes. My PhD studies have focused on the transformation of the pest *Helicoverpa armigera* with the reporter gene EGFP (enhanced green fluorescence protein). There are essentially two parts to transformation, 1) DNA delivery and 2) target gene integration. In insects the latter is commonly mediated by the use of transposons gene movement. I have used the class II transposon piggyBac to facilitate the movement of EGFP into the genome of *H. armigera* eggs as a proof of integration. Biolistics DNA delivery used microscopic gold particle coated with DNA of choice which are accelerated at high velocity into cells. In the past biolistics has been used to transform many kinds of plant tissue and has had mixed success transforming *Drosophila* eggs. Extensive attempts to adapt biolistics to transform *H. armigera* eggs proved fruitless with too many technical hurdles to over come. However, recent attempts using microinjection DNA delivery have been far more encouraging with a high frequency of transient EGFP expression and the generation of two putative EGFP stably transformed *H. armigera* lines. The development of an effective microinjection technique has also allowed us to look at the roll of RNAi in *H. armigera* with the view to using RNAi to knockdown the expression of essential/specific genes which in turn will form the basis for the development of a genetic control mechanism. By co-injecting an EGFP construct and either siRNA or dsRNA against EGFP, I have observed a significant reduction in the frequency and level of EGFP expression in eggs injected with dsRNA, and to a lesser extent with siRNA's. This indicates conservation of the RNAi pathway in *H. armigera* and will allow me to begin testing phenotypic effects of silencing new gene targets. For RNAi to be developed as an effective pest control mechanism, the parameters of RNAi in specific pests must be well understood. In specific the systemic nature or RNAi within *H. armigera* must be analysed. For RNAi to be most effective, the silencing signal must be able to spread through all cells in the organism. One gene identified in *C. elegans* known as SID-1 plays a roll in mediating the systemic spread of the RNAi signal which may involve the cell-cell movement of siRNA's. Not all organisms contain a SID-1, for example no SID-1 has been identified in *Drosophila* and as a result systemic silencing is absent. Recently I have identified 2 different SID-1-like genes in *H. armigera* and will soon be undertaking detailed spatial and functional studies once the two variants have been fully cloned. The presence of SID-1-like genes in *H. armigera* strongly suggest the presence of systemic RNAi in this organism and supports further studies into the use of RNAi as a pest control mechanism.

# Research article: *Spodoptera* EST sequencing and analysis of synteny among 3 species bring new perspectives for Lepidoptera genomics

**DOI:** 10.1673/031.007.2915

**Published:** 2007-05-04

**Authors:** d' Alençon, E. Audant, P. Bernard-Samain, S. Bidegainberry, V. Brehélin, M. Brun-Barale, A. Cousserans, C. Duvic, B. Escoubas, J-M. Feyereisen, R. Fournier, Ph. Gagneur, C. Gordon, K. Gimenez, S. Heckel, D. Hotelier, Th. Hilliou, F. Mita, K. Negre, V. Sabourault, C. Suraporn, S. Volkoff, N. Weissenbach

**Affiliations:** ^1^ Biologie Intégrative & Virologie des Insectes, INRA-UMII, Université Montpellier II, 34095 Montpellier Cedex 5, France; ^2^ Réponse Organismes aux Stress Environnementaux, INRA / Univ-Nice, 400 rte Chappes, BP167, 06903 Sophia-Antipolis Cedex, France; ^3^ Génoscope, Centre National de Séquençage, 2 rue Gaston Crémieux CP5706, 91057 Evry Cedex, France; ^4^ Ecologie Microb. Insectes & Interactions Hôtes Pathogènes, INRA-UMII, Université Montpellier II, 34095 Montpellier Cedex 5, France; ^5^ CSIRO Entomology, GPO Box 1700, Canberra ACT 2601, Australia; ^6^ Max Planck Inst. für Chemical Ecology, Hans-Knöll-Str. 8, D-07745 Jena, Germany; ^7^ Unité Informatique Centre, AgroM-INRA, 2 place Viala, 34060 Montpellier Cedex 02, France; ^8^ Insect Genome Lab., National Inst. of Agrobiological Sciences, 2–1–2 Kannondai, Tsukuba, Ibaraki 305–8602, Japan.

Species of the genus *Spodoptera* are pests which cause severe economic damage on a variety of crop plants in all continents. Several of these species are used as model for studies on pesticide resistance and host-pathogens interactions. New developments in biology and pathology of these insects now rely upon a functional genomics approach. For this purpose, we (groups 1–2 & 7, see above) established sets of cDNA from tissues which were chosen because of their importance in biological processes such as immune response, development and plant/insect interaction, *i.e*. hemocytes, midgut and fat body. A total of 29,325 ESTs sequences were obtained, which were clustered into non-redundant sets (2294 clusters and 6103 singletons) and integrated into a database. SPODOBASE (Negre *et al*., 2006, BMC Bioinformatics, submitted. http://bioweb.ensam.inra.fr/Spodobase/) is constructed in such a way that ESTs from other *Spodoptera* species can also be added. User can retrieve information using text searches, pre-formatted queries, query assistant or BLAST searches. Annotation is provided against NCBI, UNIPROT or *Bombyx mori* ESTs databases, and with GO-Slim vocabulary. The whole set of ESTs (From part of this set of data a first *Spodoptera* microarray was printed and used to study host-parasitoid interaction at the transcriptomic level, see communication by Volkoff *et al*., this meeting) was compared with databases and this revealed that 15 *%* of the *S. frugiperda* ESTs after checking that these correspond to potentially coding regions - do not have a match in BLAST searches against neither NCBI-nr nor *B. mori* EST databases. We also found that only 48 % of the contigs and 30 % of the singletons show a significant similarity with a Uniprot entry. This opens a research towards the identification of potential Lepidoptera or even *Spodoptera* specific genes. Phylogeny among Lepidoptera is still poorly known and a synteny programme will allow us to develop a comparison between gene order, gene orientation, intergenic distance and sequence similarities among different species. For this purpose a consortium of 6 laboratories launched a collaboration towards the sequencing of 60 selected genomic regions for both *Spodoptera frugiperda* and *Helicoverpa armigera*, to be then compared with the same regions of the *B. mori* genome. The regions to be sequenced were selected on the basis of the presence of genes representing different major biological functions of the insect, like immune response, olfaction, phylogenic markers, targets of insecticide resistance, P450 and GST families. BAC libraries were constructed for both species and various sequences were chosen as probes against the high-density filters. These probes were obtained either from the ESTs collections or from already sequenced cDNAs. More than 320 genes have now been identified among the first 28 BACs already sequenced representing 1.9 Mb of *S. frugiperda* and 1.5 Mb of *H. armigera* genomes. Present results also show presence of a high density of sequences displaying similarities with known repeated elements (retrotransposons with or without LTR, DNA transposons of several types). They were slightly more abundant in *S. frugiperda* than in *H. armigera* (237 *vs*. 124), and their relative frequency also varies between the two species. BAC sequencing is in progress at the French Genoscope National Center of Sequencing and results will be discussed.

# Research article: Approaches to identify sex determining genes in the lepidopteron *Maruca vitrata*

**DOI:** 10.1673/031.007.2916

**Published:** 2007-05-04

**Authors:** De Simone Anna Maria, Sorrentino Angela, Di Cara Francesca, Lino Polito, Digilio F. Anna

**Affiliations:** Institute of Genetics and Biophisics “A. Buzzati-Traverso”, Via P. Castellino 111, 80131 Naples, Italy, digilio@igb.cnr.it

The legume pod borer *Maruca vitrata* (Fabricius) (syn. *M. testulalis*) (Lepidoptera: pyralidae), is a pest of legume crops such as cowpea, pigeonpea, and common bean from the tropics to the temperate zone all over the world. Larvae damage flower buds, flowers and immature pods, causing severe economical damage to several crops. One of the most important host is cowpea (*Vigna unguiculata*) which is a highly important grain legume crop grown in semi-arid and dry savannah areas of the tropics. Cowpea provides a cheap source of nutritious food and is equally important for improving soil fertility, soil conservation and the sustainability of various cropping systems. Although the basic biology of *M. vitrata* has been studied extensively, no molecular studies have so far been carried out on this species. In order to obtain more information on this insect, we are developing tools for a molecular approach. Specifically, as a first molecular characterization of developmental genes in *M. vitrata* and with the future prospect of generating sterile mutations in this species, we decided to characterize the sex-determining genes, starting from the major determinants of the fruit fly sex-determining genes and using the knowledge gained in *Drosophila melanogaster*. Within the highly diversified Class Insecta, in spite of the existence of a variety of sex-determining mechanisms, the final regulatory genes as well as doublesex (dsx), intersex (ix) and transformer 2 (tra2) are well conserved, and their have been identified in many species other than Drosophila: *Ceratitis capitata, Megaselia scalaris, Anopheles gambiae, Musca domestica* and in the silkworm *Bombyx mori*. Based on this consideration, we started our molecular approach searching for dsx, ix and tra2 homologues in *M. vitrata*, by using *D. melanogaster* and *B. mori* genes as heterologous probes. Until now these studies allowed us to identify the M. vitrata ix gene homologue and several other genes that we can use to perform a comparative study between *Maruca vitrata* and other lepidoptera characterized to date and in broad terms between Diptera and Lepidoptera. The comparative study of these two systems (Lepidoptera and Diptera) will provide both basic knowledge and data that can be applied to develop strategies focused on biological control of this pest.

# Research article: Virulence genes of parasitoid wasps encoded by symbiotic viruses

**DOI:** 10.1673/031.007.2917

**Published:** 2007-05-04

**Authors:** J-M Drezen, A. Bezier, J. Lesobre, E. Huguet, C. Dupuy

**Affiliations:** Institut de Recherche sur la Biologie de l'Insecte CNRS, Tours, France. drezen@univ-tours.fr

Hundreds of thousand species of parasitoid wasps develop during their larval stage within the body of other insects while the adults are free. In particular 17500 species of wasps, phylogeneticaly related -the microgastoids- are obligatorily associated with viruses of the bracovirus genus. Permanently integrated into the wasp chromosomes, bracoviruses are thought to originate from the same virus integrated in the genome of the ancestor of the microgastroids that lived approx. 70 millions years ago. Bracoviruses are essential for parasitism success. Mature virions are produced in the wasp ovaries and injected by female into the host lepidopteran larvae, along with the wasp's eggs. The virus particles enter host cells where viral genes are expressed, causing several alterations to the host physiology which are beneficial for the development of the wasp progeny, comprising disruption of the immune defenses, retarded growth and inhibition of metamorphosis. Sequencing of a bracovirus genome have allowed the characterization of several gene families encoding potential factors involved in parasitism success. Interestingly some of the genes recently identified contain conserved protein domains found in virulence factors from other parasites or pathogens that are known to be involved in the suppression of the immune response of mammals. This suggests a convergent evolution between parasitoid wasps, bacterial pathogens, and parasitic nematodes (filariae) to target pathways of the host cellular response conserved in vertebrates and invertebrates. The characterization of genes conserved in the virus genomes of different parasitoid species will also be an essential tool to understand the role played by bracoviruses in the radiation of the parasitoid wasp families, highly diversified.

# Research article: Probing the insect immune system with the entomopathogen *Photorhabdus*

**DOI:** 10.1673/031.007.2918

**Published:** 2007-05-04

**Authors:** I. Eleftherianos, P. J. Millichap, G. Felföldi, F. Gökcen, N. Waterfield, D. J. Clarke, R. H. ffrench-Constant, S. E. Reynolds

**Affiliations:** ^1^ Department of Biology and Biochemistry, University of Bath, Claverton Down, Bath BA2 7AY, UK; ^2^ Department of Biochemistry, Eötvos Lörand University, Pazmany setany 1/C, Budapest, H-1117, Hungary; ^3^ Institut für Allgemeine und Spezielle Zoologie, Justus-Liebig-Universität, Stephanstr. 24, 35390, Giessen, Germany; ^4^ School of Biosciences, University of Exeter, Cornwall, UK. bssie@bath.ac.uk

*Photorhabdus* is a highly virulent pathogen that produces a range of lethal toxins to kill its insect host. Here we show that *Photorhabdus* is recognised by the immune system of its host *Manduca sexta*, as indicated by a rapid increase in the levels of mRNAs encoding three different inducible microbial recognition proteins, Hemolin, Immulectin-2 and Peptidoglycan Recognition Protein in the fat-body. RNAi knock-down of any one of these genes markedly decreased the ability of the insects to withstand infection when exposed to *Photorhabdus*. RNAi against Immulectin-2 caused the greatest reduction in host resistance to infection. The decreased resistance to infection was associated with reduced haemolymph phenoloxidase activity. We also show that the insect immune system can be effectively primed by prior infection with non-pathogenic bacteria against subsequent infection by *Photorhabdus*. Induction of this protective effect is associated with up-regulation of both microbial recognition protein genes and anti-bacterial effector genes. RNAi knock-down of individual recognition proteins had a drastic adverse effect on the *E. coli* elicited immunity. Interfering with the expression of individual antibacterial effector proteins and peptides had a much less marked effect on immunity. We present evidence that the protection elicited by previous exposure to *E. coli* is due to the presence of factors within the haemolymph plasma that inhibit the growth of *Photorhabdus*. We have also used RNAi to investigate the function of recognition protein genes expressed in *M. sexta*. haemocytes. RNAi of Hemolin expression was associated with reduced ability of haemocytes to aggregate, form nodules and phagocytose the invading bacteria. A different approach to investigate pathogen-host interactions is to identify genes that enable *Photorhabdus* to persist and multiply within the insect. In order to discover such persistence genes we screened cosmids from a fully sequenced *Photorhabdus* genome and identified a *kdp* operon that encodes the protein subunits of a bacterial K^+^ ion transporter and the two-component regulator that governs their expression. Expression of these *Photorhabdus* genes allows *E. coli* to persist within phagocytic hemocytes; disruption of any one of the operon's genes prevents persistence. Since expression of the two-component regulator genes alone is sufficient for persistence, we conjectured that the distinctive feature of *Photorhabdus* kdp is the sensor-regulator pair, which can also regulate expression of the *E. coli kdp* genes to the detriment of the host insect. We confirmed this hypothesis by showing that whereas *E. coli kdp* genes are not expressed after phagocytosis, the *kdp* genes of *Photorhabdus* are strongly expressed within *M. sexta* phagocytes. Finally we show that *Photorhabdus* produces a hydroxystilbene antibiotic compound that also acts as an inhibitor of phenoloxidase, an important component of the insect immune system. These results show not only that *Photorhabdus* is recognised by the *M. sexta* immune system but also that the insect's immune system plays an active, but ultimately ineffective, role in countering infection.

# Research article: Phylogenetic hypothesis, pattern of speciation and evolution of wing pattern in neotropical *Napeogenes* butterflies (Lepidoptera: Nymphalidae)

**DOI:** 10.1673/031.007.2919

**Published:** 2007-05-04

**Authors:** M. Elias, M. Joron, K. Willmott, V. Kaiser, K. L. Silva-Brandão, A.V.L. Freitas, C. Arias Mejía, L.M. Gomez Pineres, A.V.Z. Brower, C. Jiggins

**Affiliations:** ^1^ Institute of Evolutionary Biology, University of Edinburgh, Scotland, UK.; ^2^ Florida Museum of Natural History, University of Florida, Gainesville, USA.; ^3^ Institute de Biologia, Universidade Estadual de Campinas, São Paulo, Brazil.; ^4^ Universidad de los Andes, Bogotá, Colombia.; ^5^ Universidad de Medellín, Medellín, Colombia.; ^6^ University of Tennessee, USA. marianne.elias@ed.ac.uk

Species-level phylogenetic hypotheses can be used to explore patterns of divergence and speciation. Warningly-colored mimetic butterflies are interesting models, because their wing patterns are under strong selection, and might be involved in speciation. Here we present an almost complete phylogenetic hypothesis for the neotropical mimetic butterfly genus *Napeogenes* (20 species), based on mitochondrial genes (entire cytochrome oxydase I and II) and nuclear genes (tektin, 715bp; and elongation factor 1α, 1028bp). Whenever possible, we included several subspecies of the same species (with different wing patterns). The results showed good congruence between the different genetic regions. In most cases individuals of the same species clustered together, supporting the current taxonomy. However, *N. larina otaxes* and *N. larina aethra* appeared to be two sister species rather than two subspecies. A combined evidence topology is presented based on a Bayesian analysis of all the genes regions. Using the phylogentic hypothesis we investigate whether changes in wing pattern were associated with speciation, and whether speciation was dominantly sympatric or allopatric. Using the phylogenetic hypothesis we explore patterns of diversification, and we investigate whether changes in altitudinal range and in wing pattern were associated with speciation.

# Research article: X-tox: a new family of putative antimicrobial protein specific to Lepidoptera

**DOI:** 10.1673/031.007.2920

**Published:** 2007-05-04

**Authors:** J.-M. Escoubas, P.-A. Girard, N. Volkoff, Y. Boublik, F. Cousserans, E. D'Alençon, K. Mita, P. Taillez, M. Brehélin

**Affiliations:** ^1^ ÉCologie Microbienne des Insectes et Interactions Hâte-Pathogène, Institut National de Recherche Agronomique, Université de Montpellier II, CC54, 2 place E. Bataillon, 34095 Montpellier, France; ^2^ Biologie Intégrative et Virologie des Insectes, Institut National de Recherche Agronomique, Université de Montpellier II, CC101, 2 place E. Bataillon, 34095 Montpellier, France; ^3^ Plateforme Protéines Recombinantes, C.R.B.M., Centre National de la Recherche Scientifique, 34293 Montpellier, France; ^4^Laboratoty of Insect Genome, National Institut of Agrobiological Sciences, Tsukuba, 305-8634, Japan, jmescoubas@univ-montp2.fr

A new family of putative antimicrobial proteins, characterized by imperfectly conserved tandem repeats (up to 11) of cystein-stabilized alpha beta motifs (CS-αβ), a structural scaffold characteristic of invertebrate defensins and scorpion toxins, was identified through functional genomic approaches in the lepidopteran species *Spodoptera frugiperda*. Orthologs were also found in ESTs or genomic databases from other lepidopteran species (*Bombyx mori, Galleria mellonella, Papillo dardanus*), but not in other insect orders or other invertebrate or metazoans, thus suggesting that this protein family is specific to the lepidopteran insects. In *S. frugiperda* as well as in *B. mori*, more than one transcript can be related to this protein family. *In silico* analysis of *B. mori* genome (the sole lepidopteran genome available to date) suggests that the three mRNA encoding X-tox proteins that were characterized in this insect are generated through a mechanism of alternative splicing. This mechanism thus allows the production of different X-tox proteins containing five or six CS-αβ motifs. *S. frugiperda X*-*tox* genomic organization is currently under investigation. The role of these proteins in lepidopteran immune response was investigated by transcriptional analyses with one of the *S. frugiperda* cDNAs. Results indicate that the corresponding gene, *spod*-*11*-*tox*, is expressed mainly in larval fat body and hemocytes and that transcription is enhanced in both immune tissues upon bacterial challenge. Phylogenetic analyses show that CS-αβ motifs-containing proteins are divided into three clusters. The first, considered as “ancestral” group, comprises molecules from different invertebrate taxa (odonate insect, arachnids and mollusks) and even a fungus molecule whereas the second group includes defensins isolated from neopteran insects. Finally, the third cluster contains all the CS-αβ motifs stem from lepidopteran X-tox proteins. According to this phylogenetic analysis and to the position of lepidoptera in insect evolution (one of the latest order in insect speciation), we propose that X-tox proteins evolved from insect defensins to generate a new family of antimicrobial proteins. The main feature of this family is the multi-domain organisation who leads to molecular diversity generated through a mechanism of alternative splicing. The full characterization of this new protein family (gene organization and expression, protein structure and mechanism of action) in several lepidopteran species should contribute to solve the puzzle of the evolutionary history of arthropod defensins.

# Research article: The role of serineprotease homolgue 3 (SPH-3) in *Manduca sexta* shown by RNA interference

**DOI:** 10.1673/031.007.2921

**Published:** 2007-05-04

**Authors:** G. Felföldi, I. Eleftherianos, R. H. ffrench-Constant, I. Venekei, S. E. Reynolds

**Affiliations:** ^1^ Department of Biology and Biochemistry, University of Bath, Claverton Down, Bath BA2 7AY, UK; ^2^ Department of Biochemistry, Eötvös Loránd University, Pázmány sétány 1/C, Budapest, H-1117, Hungary; ^3^ School of Biosciences, University of Exeter, Cornwall, UK. bssie@bath.ac.uk

*Photorhabdus luminescens* lives in symbiosis with nematodes that invade insects. Following entry into the insect, the bacteria are released from the nematode gut into the open blood system of the insect. Here they secrete factors, which kill the host and also convert the host tissues into food for the replicating bacteria and nematodes. One of the secreted proteins is an rtx-like zinc metalloprotease PrtA. Using a proteomic approach to determine the natural substrates of PrtA in host haemolymph, we identified a number of *Manduca sexta* haemolymph proteins that are selectively cleaved when plasma was incubated with purified PrtA. One of the PrtA protein targets in *M. sexta* haemolymph included the Serine Protease Homologue Protein 3 (SPH-3). We found that *SPH*-*3* is not transcribed in the fat-body or haemocytes of naive *M. sexta*, but only when insects are exposed to bacteria, including *Photorhabdus*. We used RNA interference (RNAi)-mediated inhibition of expression through injection of double-stranded RNA of SPH-3 into *M. sexta* caterpillars and we achieved reduced transcription of *SPH*-*3* in both fat-body and haemocyte tissues. Knock-down of SPH-3 dramatically reduced the ability of insects to resist infection to a standard dose of *Photorhabdus*, as measured by the rate at which infected insects die. RNAi of *SPH*-*3* was also associated with decreased levels of phenoloxidase (PO) activity in haemolymph cell-free plasma from insects infected with either a non-pathogenic strain of *E. coli* or *Photorhabdus*. The reduction in PO activity was also reflected by a reduction in the number of melanotic nodules present in tissues of dissected larvae. Finally, we found that RNAi of *SPH*-*3* did not affect the transcription of pattern recognition protein genes (*Hemolin, Immulectin*-*2, Peptidoglycan recognition protein, Pattern recognition Serine Proteinase, β*-*glucan recognition protein*-*1, β* *-glucan recognition protein*-*2*), but down-regulated the transcription of certain antibacterial effector genes (*Attacin, Cecropin, Moricin*). Our findings strongly suggest that *SPH*-*3* serves specific immune functions and plays a distinctive role in *M. sexta* immune pathways.

# Research article: Host plant adaptation and specialization in the Pieridae familiy

**DOI:** 10.1673/031.007.2922

**Published:** 2007-05-04

**Authors:** H. M. Fischer, C. W. Wheat, U. Wittstock, D. G. Heckel, H. Vogel

**Affiliations:** ^1^ Entomology Dept., MPI for Chemical Ecology, D-07745 Jena, Germany; ^2^ Dept. of Biological and Environmental Science, University of Helsinki, FI-00014 Helsinki, Finland; ^3^ Inst. für Pharmazeutische Biologie, Technische Universität Braunschweig, D-38106 Braunschweig, Germany. hfischer@ice.mpg.de

A metabolic diversion of a plant chemical defense caused by an enzyme (Nitrile Specifier Protein, NSP) enables a majority of Pieridae species to feed on Brassicaceous plants. The NSP is a newly recruited detoxifying protein in this family. It shows a specific repeat structure and has no sequence homology to any protein with a known function. We are trying to unravel the molecular mechanisms leading to the evolution of this protein and the subsequent adaptation to a plant defense system. The nucleotide sequence is known for two model species. By generating cDNA libraries of related species and amplifying intron and upstream regions, species and allelic differences are being located. Although NSP shows no sequence homology to any protein of known function, single domains show similarities to the so-called major allergen proteins, found in the gut lumen of many insect species. By establishing heterologous expression systems for both proteins we are trying to investigate the mode of action of NSP and the function of MA.

# Research article: The effect of microorganisms in the food on the growth rate and immune system of cabbage looper (*Trichoplusia ni*)

**DOI:** 10.1673/031.007.2923

**Published:** 2007-05-04

**Authors:** D. Freitak, D. Heckel, H. Vogel

**Affiliations:** Department of Entomology, Max Planck Institute for Chemical Ecology, Hans Knöll Str. 8, Jena 007745, Germany. dfreitak@ice.mpg.de

One way to sense the pathogen load in the environment is through the food, lepidopteran larvae are consuming vast amount of food during their relatively short lifespan, this leads to the intake of large amount of possibly harmful microorganisms. Many pathogens are using digestive system as a route to enter the host. We hypothesize that this should have lead to the evolution of pathogen recognition capacities of the digestive system and the mounting of an effective immune response after recognition. In my experiments I raised *T. ni* larvae on sterile artificial diet and on artificial diet contaminated with bacteria. I collected the hemolymph and midguts of last instar larvae and measured the antibacterial activity and protein expression of the hemolymph. Furthermore, to estimate the status of the immune system, I measured the general lysosomatic and phenoloxidase activity. Larvae grown on the bacteria-contaminated diet showed higher steady state lytic activities and arylphorin concentration in their hemolymph. Contrary to this finding, phenoloxidase activity was higher in the individuals grown on the diet without bacteria. The type of diet also showed an effect on the growth and pupation of larvae. Larvae who were fed on diet contaminated with bacteria needed more time to reach the pupal state and had a lower pupal weight. This kind of effect is mediated via digestive system. I used Real-Time PCR to check the differential expression level of different immune response related genes and found out that several genes connected to synthesis of antibacterial proteins are up regulated in the midgut of the larvae grown on bacterial diet.

# Research article: Flapless phenotype caused by the partial deletion of the Z chromosome in the silkworm, *Bombyx mori*

**DOI:** 10.1673/031.007.2924

**Published:** 2007-05-04

**Authors:** T. Fujii, H. Abe, K. Mita, S. Katsuma, T. Shimada

**Affiliations:** ^1^ Department of Agricultural and Environmental Biology, Graduate school of Agricultual and Life Sciences, The University of Tokyo, Tokyo 113–8657, Japan; ^2^ Department of Biological Production, Faculty of Agriculture, Tokyo University of Agriculture and Technology, Tokyo 183–8509, Japan; ^3^ Laboratory of Insect Genome, National Institute of Agrobiological Science, Tsukuba 305–8634, Japan. tsuguru2002@yahoo.co.jp

The silkworm, *Bombyx mori*, has a sex-determination system (ZW female: ZZ male) in which a W chromosome epistatically determines femaleness. Therefore, the putative *Fem* (female determinant) gene is assumed to be localized on the W chromosome. So far, 12 W-specific RAPD markers (W-RAPD) have been identified in the silkworm strains maintained in Japan. However, because recombination is restricted to the male in *B. mori*, genetic mapping of these W-RAPD markers and *Fem* gene to the W chromosome is impossible. On the other hand, the relative positions of the W-RAPD markers on the W chromosome can be determined using deletion mapping. In deletion-mapping of W-specific RAPD markers, we used X-ray irradiation to break the translocation-carrying W chromosome (W^Z*e*^). We succeeded in isolating a fragment of the W chromosome (Z*e* ^w^) having three of 12 W-Specific RAPD markers. It was revealed that the Z*e* ^w^ fragment does not contain *Fem* gene because it was transmitted to males. Unexpectedly, we found that the Z chromosome was also broken into a large fragment (ZL) having the +*sch* (1-21.5) and a small fragment (ZS) having the + (1-46.9) by X-ray irradiation. Moreover, it was revealed that a new chromosomal fragment (Ze^w^Zs) was generated by a fusion event between the *Ze* and the ZS fragments. We analyzed the genetic behavior of the ZL fragment and the Z*e* ^w^ZS fragment during female (2A : ZL Z*e* ^w^ZS/W) and male (2A : Z/ZL Z*e* ^w^ZS) meiosis using phenotypic markers. It was observed that the ZL fragment and the W or the Z chromosomes separate without fail. On the other hand, non-disjunction between the Z*e* ^w^ZS fragment and the W chromosome and also between the Z*e* ^w^ZS fragment and the Z chromosome occurred. Furthermore, the males (2A : Z/ZL) resulting from non-disjunction between the Z*e* ^w^ZS fragment and the W chromosome or the Z chromosome had a phenotypic defect. Namely, Z/ZL male moths could not flap their wings due to indirect flight muscle dystrophy while they could walk and copulate. It was confirmed that the ZL fragment did not cause the flapless phenotype because Z/ZL Z*e* ^w^ZS male could flap their wings vigorously. These results suggest that there is haploinsufficient gene(s) which are involved in indirect flight muscle development on the ZS region of the Z chromosome. In *B. mori*, absence of dosage compensation is suggested because 12 of 15 genes on the Z chromosome expressed more abundant mRNA in males than in females. In contrast, mRNA amounts of three of 15 genes on the Z chromosome were equivalent in males and females, or female biased (Koike et al., 2003). Therefore, if there are haploinsufficient gene(s) on the ZS region of the Z chromosome, the expression of the gene(s) may be up-regulated in some stage of female (Z/W) development.

# Research article: Molecular mechanisms of the stage-specific larval pattern formation in the swallowtail butterfly, *Papilio xuthus*

**DOI:** 10.1673/031.007.2925

**Published:** 2007-05-04

**Authors:** R. Futahashi, H. Fujiwara

**Affiliations:** Dept. of Integrated Biosciences, University of Tokyo, Kashiwa, Chiba 277–8562, Japan. kk37524@mail.ecc.u-tokyo.ac.jp

Like the adult wings, the larvae of butterflies and moths are unique in their colouring, shape, and patterns. Because butterflies and moths spend most of their lives as soft-bodied larvae, larval body patterns of them are conspicuous traits for natural selection. However, the molecular bases of insect larval pattern formation are largely unknown. The larva of the swallowtail butterfly *Papilio xuthus* changes its colour pattern dramatically during the fourth ecdysis. In the juvenile instars of *P. xuthus*, the larva have brownish-black integuments with white markings, which is inferred to mimic a bird dropping. On the other hand, the fifth (last) instar has a green camouflage colour, which is believed to allow the larger larvae to avoid predators better than the bird-dropping pattern. A comparison of the penultimate and final instar of *P. xuthus* should be useful in elucidating the regulation of larval pattern formation. In the present study, we aimed to identify the genes involved in the stage-specific larval mimicry markings of *P. xuthus*, and the hormonal control of their expression. We first compared the mRNA expression of epidermis between the third and fourth molts of *P. xuthus* using cDNA subtraction method. After analyzing 2,072 clones from two subtractive libraries, we obtained 31 and 64 candidate genes for final- or penultimate-instar-specific genes, respectively. The expression pattern of each gene during the second, third and fourth molts was examined by RT-PCR and whole-mount *in situ* hybridization. Among final-instar-specific genes (Fsg), *Fsg02* and *Fsg20* were expressed at the presumptive green region only during the fourth molt, suggesting that *Fsg02* and *Fsg20* were correlated with green coloration in the final instar. Among penultimate-instar-specific genes (Psg), we identified 10 cuticle genes expressing specifically in tubercle structures during the third molt. These genes were inferred to be involved in the formation of the unique exoskeletal structure observed in the juvenile instar. We next investigated the hormonal effect on the larval patterning. We found that the addition of juvenile hormone analogue to the 4th instar altered the fifth instar pattern into the juvenile-instar type, suggesting that juvenile hormone regulates the stage-specific larval pattern change. This study provides novel molecular markers and insights into the molecular mechanisms of the larval pattern formation and pattern change.

# Research article: Novel genes differentially expressed between posterior and median silk gland identified by SAGE-aided transcriptome analysis

**DOI:** 10.1673/031.007.2926

**Published:** 2007-05-04

**Authors:** Annie Garel, Jérôme Briolay, Patrick Brouilly, Corinne Royer, Shun-ichi Sasanuma, Motoe Sasanuma, Céline Keime, Olivier Gandrillon, Gérard Chavancy, Kasuei Mita, Pierre Couble

**Affiliations:** ^1^ Centre de Génétique Moléculaire et Cellulaire, UMR CNRS 5534, Université Claude Bernard Lyon 1, 43 Boulevard du 11 Novembre 1918 69622 Villeurbanne Cedex, France.; ^2^ Unité Nationale Séricicole, INRA, 25 Quai Jean Jacques Rousseau - 69350 La Mulatière, France.; ^3^ National Institute of Agrobiological Sciences, Owashi 1–2, Tsukuba, Ibaraki 305–8634, Japan. Royer@cismsun.univ-lyon1.fr, pierre.couble@univ-lyon1.fr

To identify functions that distinguish the posterior silk gland (PSG) and the median silk gland (MSG) of *Bombyx mori*, specialized in the production and secretion of fibroin and sericins respectively, serial analysis of gene expression (SAGE) profiles of PSG and MSG cells were analyzed and compared. 41311 and 22078 tags were extracted from respectively the fibroin and the sericin secreting cells. The construction of a *B. mori* reference tag collection from published resources and from a set of 38000 *Bombyx* EST sequenced from the 3′ side, helped us to identify a mRNA to more than 60% of the different tags whose incidence was found at more than one exemplary in the two SAGE libraries. Strikingly, a series of highly or mid abundant tags was identified in the MSG tag collection, that did not show up in the PSG SAGE library. cDNA corresponding to 19 of these MSG-specific tags were full-length sequenced. Most of these cDNA corresponded to mRNA encoding protéiens of unknown functions. Four of them were found highly abundant and coding virtually secreted proteins. Current efforts aim at identifying the role of these proteins in the secretion and spinning of silk. The analysis of these novel transcripts allow to suggest that middle silk gland cells realize more diversified functions than that already documented, of synthesis and secretion of sericins, the water soluble components of silk.

# Research article: Hemolin and antiviral defence in Lepidoptera

**DOI:** 10.1673/031.007.2927

**Published:** 2007-05-04

**Authors:** M. Geber, I. Faye, O. Terenius

**Affiliations:** Department of Genetics, Microbiology and Toxicology, Stockholm University, Stockholm, Sweden.ingrid.faye@genetics.su.se

Hemolin, an immune protein belonging to Immunoglobulin gene superfamily is so far only demonstrated to exist in lepidopteran insects. Hemolin is upregulated by viral as well as bacterial infections and in addition, by ecdysone. Several of the putative regulatory elements responding to these inducers are conserved in the promoter region of the Hemolin genes in *Hyalophora cecropia* and *Manduca sexta*. One of the conserved upstream elements falls under the consensus of mammalian interferon regulatory factor elements IRF-E and its involvement in the regulation of Hemolin by viruses and the signalling pathways involved remains to be investigeted. From early experiments with *H. cecropia* it is clear that its NFχB homolog, CIF is not activated during viral challenge. Moreover, it has been shown that partial dsRNA silencing of the Hemolin gene in the Chinese Oak silk moth, *Anthereae pernyi* increases the progress of an infection by ApNPV in the pupal stage. Recently, we decided to look at the co-evolution of Hemolin and baculoviruses, specific for the different lepidopteran species used. Apart from the six hemolin genes that were already cloned, the longest exon (IV) was cloned from dried specimens of three additional species. From baculoviruses earlier isolated from these lepidopterans, the polyhedrin genes were available in databases and used in the phylogenetic comparisons. The general evolutionary relationship between lepidopteran species was based on the gene for elongation factor 1a, also available in the databases. This study is still in progress and data achieved so far will be presented and discussed.

# Research article: Estimating genome coverage of lepidopteran BAC libraries by screening with single copy probes

**DOI:** 10.1673/031.007.2928

**Published:** 2007-05-04

**Authors:** M. Goldsmith, D. Proestou, D. Carter, E. Nicholson, C. Wu, H. Zhang

**Affiliations:** ^1^ Biological Sciences Department, University of Rhode Island, Kingston, RI, USA; ^2^ Department of Soil & Crop Sciences, Texas A&M University, College Station, TX, USA. mki101@uri.edu

We constructed large insert BAC libraries in the vector, pECBAC1, for *Manduca sexta* (Ms), *Heliconius erato* (He), and *Heliothis virescens* (*Hv*). Two libraries were generated for each species using partial digests of pupal DNA with BamHI or EcoRI. Based on haploid genome size (*M. sexta*, 500 Mb, *H. erato*, 395 MB, and *H. virescens* 400 Mb, J.S. Johnston, personal communication), mean clone insert size (150–175kb), and number of clones (19.2–21.5 X103), we estimated the genome coverage per library to be in the range of 6–9-fold. For an independent estimate of genome coverage, we screened high density BAC filters with putative single copy genes in the form of cloned cDNA or PCR-amplified genomic DNA, using the number of hits as a measure of genome redundancy. We used the non-radioactive Amersham ECL Direct system to label probes and detect hybridization by chemiluminescence with X-ray film. Advantages of this method include simple, rapid probe labeling and filter washing protocols coupled with the ability to rehybridize filters without stripping, although we found some signal carry over despite waiting the prescribed period of time before reusing filters. Our primary considerations for probe design and general findings were: 1) Probe length. Although we were able to detect positive signals on filters hybridized with probes as short as 350–400 bp, probes of 1 kb or greater gave more consistent signal-to-noise ratios. 2) Use of well-conserved sequences. To increase the information obtained from our validation studies, we asked colleagues for probes concerning key areas of research using lepidopteran models. These included genes whose products are involved in olfaction (*Ms*OR1, *Ms*OR3), nerve axon growth and guidance (*Ms*Nos128, *Ms*Eph, *Ms*FasII, *Ms*PlexA), hormone action (*Ms*Broad, *Ms*E75, *Hv*PTTH), wing patterning (*He*wg, *He*ptc, *He*Ci), Bt toxin action (*Hv*APN 120, *Hv*cad), and ribosome structure (*Hv*RpS4, *He*RpS5, *He*RpS9, *He*RpL3, *He*RpL10). We reasoned that, considering the paucity of genomic data for lepidopteran species, identifying BACs for well-conserved genes would be of value for future comparative genomics studies. 3) Avoidance of conserved domains. The lack of large-scale genome sequence data for these species made it difficult to predict whether genes for related proteins containing conserved domains were present which might generate duplicate hybridization signals. This problem first arose with a probe for *Ms*Broad containing two zinc fingers which generated twice the number of hits than average. 4) Attention to the presence of introns. Some probes amplified from genomic DNA contained introns, based on greater sequence length than predicted from cDNAs. These often gave an unexpectedly high number of positive signals, indicating the presence of a repeated element. Thus, we tried to avoid probes containing introns unless they were shown to be “clean” in this respect. 5) Amplification of cDNA-containing plasmid inserts with insert-specific primers. Initially we used universal sequencing primers to amplify inserts in plasmid vectors. In some cases the presence of short vector-specific sequences remaining on the PCR products cross-hybridized extensively with the pECBAC1 vector, generating high filter backgrounds. We subsequently switched to primers within the insert sequence. Of interest is that we usually obtained sufficient background on the filters to determine the address of positive clones despite having no known vector sequence in the probe. So far we have tested each library with 6–11 probes, of which 63–85% percent gave well-defined signals. By this approach library coverage averaged as expected for the *M. sexta* libraries but less than the values based on BAC library DNA content for the *H. erato* and *H. virescens* libraries. Possible explanations for the discrepancy will be discussed. Supported by NSF Grant IBN-0208388 to MRG, CW, and HZ. We thank L. Riddiford, F. Gould, and O. McMillan for generous gifts of insects, and A. Nighorn, H. Robertson, L. Riddiford, X. Zhou, P. Copenhaver O. McMillan, S. Gill, L. Gahan, F. Gould, R. Palli, and D. Knipple for probes.

# Research article: Developmental paradigm for the silk glands in the mulberry silkworm *Bombyx mori*: Are they different from the salivary glands?

**DOI:** 10.1673/031.007.2929

**Published:** 2007-05-04

**Authors:** K. P. Gopinathan, R. Parthasarathy, S. Dhawan

**Affiliations:** Microbiology & Cell Biology Department, Indian Institute of Science, Bangalore 560 012, India. kpg@mcbl.iisc.ernet.in

*Bombyx mori* has long been used as a lepidopteran model system for basic studies on gene expression and its regulation. Recently the entire genome of *B. mori* comprising of about 500 million base pairs has been sequenced. However, the developmental studies at the molecular and genetic levels on this economically important organism has lagged considerably behind *Drosophila*, which has served as the paradigm for development. We have been exploiting *B. mon* as a model organism for development for the past several years. There are substantial differences in the developmental programmes between *Drosophila* and *Bombyx*. Although the overall programming of appendage development in both the organisms follow common principles, the timings of operation of the patterning mechanisms appear to be significantly different. The silk proteins are produced in the silk glands of *B. mori*, a pair of tubular structures arising from the labial segment and extending in length all the way up to the caudal region. The silk glands are functionally divided into three distinct compartments, the anterior (ASG), middle (MSG) and posterior (PSG) silk glands. PSG synthesizes the silk fibre proteins and MSG synthesizes the silk glue proteins, the sericins. We have identified the operation of the canonical *Wnt* signaling pathway in the subcompartment specification within the MSG to result in the territorial regulation of the different sericin proteins. On the other hand, the PSG specification is through the *Ubx* signaling pathways. The silk glands are often considered as modified salivary glands because of their labial origin, but unlike *B. mori, Drosphila* lacks the silk glands. *B. mori* additionally has a pair of salivary glands originating from the mandibular segment. However, there are no subcompartments within the salivary glands. Although there are similarities between the two sets of glands, there are also substantial differences between them.

# Research article: Functional genomics of the midgut of the cotton bollworm, *Helicoverpa armigera*

**DOI:** 10.1673/031.007.2930

**Published:** 2007-05-04

**Authors:** K. Gordon, G. Colebatch, P.M. Campbell, I. Horne, P.D. East

**Affiliations:** CSIRO Entomology, Canberra, ACT, Australia. karlg@ento.csiro.au

The lepidopteran insect, *Helicoverpa armigera*, or cotton bollworm, is a major pest of agriculture worldwide. It is characterised by a wide host plant range and the ability to develop resistance to many control agents. The larval midgut plays a central role in both interactions, being responsible for both digestion and defence against key environmental stresses -e.g. host plant defence chemicals or chemical pesticidal agents. It is the primary target of biological control agents like Bt, now widely used in transgenic crops for pest control. We are undertaking a functional genomics study of the midgut in this insect in order to understand the molecular basis of digestion, detoxification and resistance. Furthermore, this insect's ability to become resistant to chemical pesticides, coupled with the recent detection of genes for Bt-resistance, means an ongoing need for research into novel biological methods for its control. We have undertaken an EST project to identify genes important for midgut function, growth and development and gene/enzyme pathways involved in insect-plant and insect-pathogen interactions. Sequence analysis of midgut cDNA libraries now covers ∼5,000 ESTs each from young (2nd and early 3rd instar) and from 5^th^ instar larvae. Clustering yielded a unigene set of ∼3,900 contigs. Searches against databases such as UniProt (EBI) and model organism proteomes yielded confident Gene Ontology annotation for ∼55% of the contigs. These include numerous genes associated with key midgut functions: digestive hydrolases; nutrient and ion transport and pH maintenance; a range of detoxification enzymes and genes forming the peritrophic membrane or involved in midgut cell proliferation, differentiation and adhesion. Many have not previously been identified in lepidopteran systems. Additionally, comparison with silkworm genes identified likely orphan lepidopteran genes. Some of the main groups we have analysed to date are: Proteases: Over 80 proteases have been identified. These are predominantly trypsins, with some chymotrypsins and a number of diverged proteases that may be inactive. A variety of amino- and carboxy-peptidases have also been identified. Lipases: Over 20 lipases have been identified. These include a number of diverged lipases that may be inactive. Detoxification: A number of representatives from each of the major classes of detoxification enzymes have been obtained. These include: 20 cytochrome P450 mono-oxygenases (Cyp450s), 6 glutathione-S-transferases (GSTs) and 28 carboxylesterases. Peritrophic membrane components: Three different types of proteins have been identified to date. These are several types of intestinal mucin, that have highly glycosylated mucin-like domains interspersed with cysteine-motif chitin-binding domains (CBDs); these appear to be unique to lepidoptera and appear likely to be an adaptation to their diet rich in plant matter that generates significant (oxidative) stress for the gut epithelium. A further type of peritrophin carries only CBDs; shorter versions of this are found in other arthropods. The third type contains a chitin deacetylase domain (CDA), either alone or with a CBD. These are widespread in arthropods. Immobilised enzymes appear to represent a further class of PM proteins. Microarrays are being used for expression profiling studies in the midgut and have identified numerous genes whose expression shows spatial variation. Expression of genes encoding PM components has been further studied using RT-PCR, whole-mount and in-situ hybridization experiments. Generation of this defence structure is ongoing along the midgut.

Identification of genes that may underlie Bt resistance in Helicoverpa, e.g. from families such as the candidate receptor aminopeptidases, provides a basis for development of strategies for mapping candidate resistance genes. Finally, the cDNA sequences are being used to screen BACs for sequencing by INRA (in collaboration with R. Feyereisen).

# Research article: Molecular phylogenetics and the evolution of mimicry in the butterfly genus Basilarchia

**DOI:** 10.1673/031.007.2931

**Published:** 2007-05-04

**Authors:** T.M. Hughes, J.M. Marcus

**Affiliations:** Department of Biology, Western Kentucky University, Bowling Green, KY USA. morrit@wku.edu

The butterfly genus Basilarchia contains several well-known examples of mimicry, even though the majority of species in this genus do not mimic other butterflies. The orange and black viceroy butterfly (Basilarchia archippus) mimics the distasteful monarch butterfly (Danaus plexippus) through much of its range, but the viceroy subspecies found in Florida (B. archippus floridensis) mimics the queen butterfly (Danaus gilippus berenice) instead. Closely related to the viceroy is the red-spotted purple butterfly (B. arthemis astyanax), which is bright blue in color and thought to mimic the distasteful pipevine swallowtail butterfly (Battus philenor). Competing hypotheses for the origin of mimicry in this genus either suggest that mimicry evolved only once followed by a series of model switching events, or that mimicry has arisen independently at least twice in this genus. We tested these hypotheses by generating a molecular phylogeny based on DNA sequences for three genes (cytochrome oxidase I, cytochrome oxidase II, and wingless) obtained from representatives of each of the butterflies in the genus Basilarchia, plus representative outgroups. Our results support the hypothesis of two independent origins of mimicry in the genus Basilarchia, but they also suggest that the viceroy is much more distantly related to the red-spotted purple than had previously been suspected.

# Research article: Role and evolution of viral cystatins in an insect host-parasite interaction

**DOI:** 10.1673/031.007.2932

**Published:** 2007-05-04

**Authors:** E. Huguet, C. Serbielle, V. Douris, G. Lalmanach, K. Iatrou, J-M Drezen

**Affiliations:** ^1^ Institut de Recherche sur la Biologie de l'Insecte, UMR CNRS 6035, Faculté des Sciences et Techniques, Parc de Grandmont, 37200, Tours, France; ^2^ Institute of Biology, National Centre for Scientific Research “Demokritos”, P.O. Box 60228, Aghia Paraskevi 15310 Athens, Greece; ^3^ INSERM U 618 «Protéases et Vectorisation Pulmonaires », Université François Rabelais, Faculté de Médecine, 8 ter, Boulevard Tonnellé, 37032 Tours cedex, France. elisabeth.huguet@univ-tours.fr

Parasitoid wasps have developed amazing strategies to parasitize their insect hosts. One of the most effective involves the domestication of polydnaviruses (PDV). Viral DNA is integrated into the genome of the wasp and is transmitted vertically via wasp chromosomes. Virus excision and replication occurs only in wasp ovaries resulting in the production of segmented double stranded DNA circles which are injected in the host during wasp oviposition. Expression of viral genes inside the insect host alters its immune response and developmental program resulting in the emergence of adult parasitoid wasps. PDVs are divided in two genera, ichnoviruses and bracoviruses, which are associated with tens of thousands of species of parasitoid wasps belonging to ichneumonid or braconid families. *Cotesia congregata*, is a braconid wasp that injects a bracovirus (*Cotesia congregata* Bracovirus, CcBV) during parasitism of the tobacco hornworm caterpillar, *Manduca sexta*. Complete sequencing of the CcBV genome has revealed the presence of numerous genes possibly involved in host deregulation. Here we describe the characterization of a CcBV multigene family encoding three proteins with homology to members of the cystatin superfamily. Cystatins are tightly binding reversible inhibitors of cysteine proteases. Cystatins have been described in numerous organisms and are often involved in host-pathogen interactions either acting as host defense proteins or as virulence factors. In filarial nematodes, for example, they account for a major portion of the immunosuppressive activity of secreted filarial proteins and are considered as major pathogenicity factors. Several lines of evidence suggest that viral cystatins could also play an important role in lepidopteran host immune suppression. Viral cystatins are expressed at an early stage and reach very high levels of expression during parasitism. Furthermore, the purification of a recombinant form of one of the CcBV cystatins, cystatin1, revealed that this viral cystatin is functional having potent inhibitory activity towards the cysteine proteases papain, human cathepsins L and B and *Sarcophaga* cathepsin B in assays *in vitro*. The identification of the targets of the viral cystatins in the host caterpillar should now help us to deduce the host physiological processes affected by viral cystatins.

# Research article: Genomic and nongenomic actions of 20E in programmed cell death of *Bombyx* anterior silk gland

**DOI:** 10.1673/031.007.2933

**Published:** 2007-05-04

**Authors:** Masatoshi Iga, Takayuki Sekimoto, Mohamed Elmogy, Masafumi Iwami, Sho Sakurai

**Affiliations:** Division of Life Sciences, Graduate School of Natural Science and Technology, Kanazawa University, Kanazawa 920–1192, Japan. ssakurai@kenroku.kanazawa-u.ac.jp

Anterior silk glands (ASGs) of the silk worm *Bombyx mori* undergo programmed cell death (PCD) in prepupal period in response to the large increase in hemolymph ecdysteroid titer. ASGs loose the sensitivity to JH from day 3 (V3) to 5 of the fifth instar and acquire their responsiveness to 20-hydroxyecdysone (20E) from V5 to V6, the day of gut purge. Then the PCD sequence begins 2 days after gut purge (G2) at and after which 20E is not required for completing PCD. We examined the expression profiles of 11 transcription factor genes in the fifth instar. Developmental profiles of expressions of early genes (two isoforms of each of *EcR, usp, E74, E75* and three isoforms of *BR*-*C*), an early-late gene (*BHR*-*3*) and β*FTZ*-*F1* indicated that these genes were divided into two groups. Genes of one group are *EcR*-*B1, usp*-*1, E74B* and β*FTZ*-*F1*, which were maximally expressed from V4 to 6, indicating that those genes could be involved in the acquisition of competence to respond to 20E. The other group genes, that were up-regulated after gut purge, are *EcR*-*A, usp*-*2, E74A, E75A, E75B* and *BHR3*, showing a possible involvement of these genes in PCD execution. Three *BR-C* isoforms (Z1, Z2 and Z4) were induced in a stepwise manner; first increase from V5 to V6 followed by the second increase from G1 to G2. These expression profiles are different from those found in *Drosophila* salivary glands during the period from the time shortly before puparium formation to pupation. Go ASGs undergo PCD when cultured with 1 µM 20E. During the first 24 h of the culture, *EcR*-*A, usp*-*2* and *E75A* were up-regulated in 1 h while *E74A, BRH*-*3* and three *BR*-*C* isoforms were at 8 h. By contrast, *E74B* did not respond to 20E and *usp*-*1* was rather suppressed by 20E. Interestingly, Co-existence of CHX, a translation inhibitor, with 20E enhanced greatly the 20E-induced expressions of *EcRiB1, usp*-*1, E74B* and *E75B*, although these genes were immediate response genes. This indicates the presence of suppressing factors of those genes. 20E-concentration responses for gene expression revealed that the optimum 20E concentrations for individual gene expressions coincided with the hemolymph ecdysteroid titer at the time when individual genes were up-regulated in vivo. Accordingly, the differential gene expressions of those transcription factor genes are brought about by the changes in ecdysteroid titer. In an in vitro culture of Go ASGs with 1 µM 20E, an addition of α-amanitin, a transcription inhibitor, from 0 h of culture completely inhibited PCD while that at 8 h allowed ASGs to undergo PCD with a slight delay, showing that gene expression required for PCD is completed by 8 h of 20E stimulation. By contrast, when ASGs were transferred differentially to a hormone-free medium, 20E was revealed to be required until 42 h of the culture for competing PCD. These results suggest that PCD of ASGs is governed by two different molecular mechanisms, one associated with de novo gene expression and the other mediated by nongenomic action of 20E. The non-genomic action was analyzed enzymatically by assessing activities of protein kinase C (PKC) and caspase 3-like protease and pharmacologically using various inhibitors and membrane-permeable second messenger analogues. PCD of ASGs proceeds in the order of cell shrinkage, nuclear condensation, DNA fragmentation, nuclear fragmentation and apoptotic body formation. We found that 0.2 mM CHX inhibited protein synthesis but not induced PCD. When ASGs were exposed to CHX and 20E, nuclear condensation, DNA fragmentation and nuclear fragmentation occurred, an indication that those responses do not depend on 20E-induced gene expression. In the nongenomic action of 20E, 20E acted through a calcium-PKC-caspase 3-like protease pathway for inducing DNA fragmentation and nuclear fragmentation, while PKC and caspase 3-like protease activation were not involved in inducing nuclear condensation. Although Ca2+ signal was prerequisite for nuclear condensation, calcium ionophore was capable of mimicking 20E only after 18 h preincubation of ASGs with 20E, indicating the cross talk of genomic and nongenomic action of 20E for completing the PCD.

# Research article: An EST approach for the molecular dissection of olfactory reception in a crop pest, the cotton leafworm *Spodoptera littoralis*

**DOI:** 10.1673/031.007.2934

**Published:** 2007-05-04

**Authors:** E. Jacquin-Joly, C. Merlin, S. Malpel, J. Pelletier, I. Brigaud, M-C. François, M. Maïbèche

**Affiliations:** UMR « Insect Physiology: Signalization and Communication », INRA Versailles, Route de Saint-Cyr, F-78026 Versailles Cedex, France. jacquin@versailles.inra.fr

In insects, among them economically important agricultural pests and disease vectors, olfaction plays a vital role in all aspects of their life. In particular, chemical signals underlie behaviors that are critical for mate (via sexual pheromones) or host recognition and selection (via allelochemicals). A better understanding of the molecular basis of this chemical communication could result in identifying new targets for future strategies to reduce their negative effects. Focusing on the major cotton pest, the noctuid moth *Spodoptera littoralis*, we set up an EST approach to dissect the olfactory reception mechanisms in the antennae. Indeed, recognition and selection of specific odorants by the antennae relay on the combinatorial expression and intervention of a multitude of different actors, most of which remain to be discovered, such as olfactory receptors or ion channels. Indeed, only few olfactory receptors are known to date in insects and they appear as new G-protein coupled receptor families of extremely divergent genes. Our EST strategy consisted in the elaboration of a cDNA library constructed from RNA extracted from 12000 *S. littoralis* male antennae, including a normalization step (Invitrogen, CA, USA) to enrich the library in rare transcripts, favouring the discovery of novel genes. Two thousands clones from the library were randomly sequenced from their 5′ end and sequences were analysed and compared to public databases using bioinformatics. This led to the identification of a panel of molecular elements potentially involved in the peripheral olfactory steps in *S. littoralis*. A repertoire of putative olfactory genes was established, including odorant-binding proteins, olfactory receptors, degrading enzymes, ion channels. In particular, different elements involved in the sex pheromone detection pathway were discovered, such as pheromone-binding proteins and putative pheromone receptors. In addition, numerous modulating elements (hormone receptors, clock genes) were discovered, suggesting that the olfactory response may be modulated by endogenous/exogenous factors at the peripheral level (hormonal statute, circadian clock). In collaboration with the French National Centre of Sequencing (Génoscope, Evry), high-throughput sequencing of the antennae library is under way to complete the antennal olfactory scheme in *S. littoralis*.

# Research article: Analysis and modification of protein *N*-glycosylation pathways in insect systems

**DOI:** 10.1673/031.007.2935

**Published:** 2007-05-04

**Authors:** D.L. Jarvis, J.J. Aumiller, C. Geisler, J. Hensley, J.R. Hollister, X. Shi

**Affiliations:** Department of Molecular Biology, University of Wyoming, Laramie, WY, USA. dljarvis@uwyo.edu

As eukaryotic organisms, insects have the enzymatic machinery needed to *N*-glycosylate their proteins. Insect protein *N*-glycosylation pathways generally appear to be intermediate in complexity relative to lower eukaryotes, such as yeasts, and higher eukaryotes, such as mammals. More specifically, insects generally perform the same early steps of protein *N*-glycosylation, which include *N*-glycan assembly, transfer, and trimming, as lower and higher eucaryotes. But, they lack some of the functions required for *N*-glycan elongation. Thus, the major processed *N*-glycans produced by insects are relatively simple paucimannose structures (Man3GlcNAc2-R) with or without core fucose residues. An important implication of these observations is that recombinant *N*-glycoproteins produced in insect-based systems might not have structurally authentic glycans. In fact, recombinant *N*-glycoproteins produced using baculovirus-insect cell systems typically lack the complex, terminally sialylated glycans found on many native mammalian glycoproteins. The insect-derived products typically have paucimannose N-glycans at the sites occupied by complex, terminally sialylated N-glycans in the native mammalian products. In recent years, we have been addressing this problem by using metabolic engineering methods to extend the protein *N*-glycosylation pathway of lepidopteran insect cell lines. These efforts have yielded transgenic insect cell lines that encode and express mammalian glycosyltransferases and enzymes involved in CMP-sialic acid biosynthesis. Relative to the parental lines, these new cell lines can still serve as hosts for baculovirus expression vectors and produce similar levels of recombinant glycoproteins. Unlike the parental insect cell lines, however, the transgenic lines can produce recombinant glycoproteins with complex, terminally sialylated *N*-glycans. This presentation will focus on the production and characterization of these transgenic insect cell lines and their functional capabilities.

# Research article: The evolutionary importance of mimicry in *Heliconius* butterflies

**DOI:** 10.1673/031.007.2936

**Published:** 2007-05-04

**Authors:** Chris D Jiggins

**Affiliations:** Institute of Evolutionary Biology, University of Edinburgh, Edinburgh EH9 3JT, UK. chris.jiggins@ed.ac.uk

*Heliconius* butterflies are well known for their bright mimetic colour patterns. Here I describe studies of speciation among the *Heliconius melpomene* species group. Differences in colour pattern that distinguish species involve the same genetic basis as within-species variation. Furthermore colour patterns are used as cues in mating, and therefore pattern shifts lead to reproductive isolation and contribute to speciation. In one Colombian species, *Heliconius heurippa* it has been suggested that a novel pattern may have arisen as a result of hybridisation between *H. melpomene* and *H. cydno*. By means of crosses we have demonstrated that the *H. heurippa* pattern can be re-created within three generations of hybridisation in the laboratory. Furthermore, both elements of the hybrid pattern are necessary for mate recognition, such that the hybrid trait is clearly causing reproductive isolation between natural populations. Other examples suggest that a hybrid origin for novel patterns may be relatively common in these butterflies. A definitive test of this hypothesis awaits identification of the genes at a molecular basis.

# Research article: Conserved but flexible: Genetic control of mimicry in *Heliconius* butterfly wing patterns

**DOI:** 10.1673/031.007.2937

**Published:** 2007-05-04

**Authors:** Mathieu Joron, Riccardo Papa, James Mallet, W. Owen McMillan, Chris D. Jiggins

**Affiliations:** ^1^ Institute of Evolutionary Biology, University of Edinburgh, West Mains Road, EH9 3JT, Edinburgh, United Kingdom; ^2^ Institute of Biology, Leiden University, Kaiserstraat 63, 2311 GP Leiden, The Netherlands; ^3^ Department of Biology, University of Puerto Rico, P.O. Box 23360, PR 00931, San Juan, Puerto Rico; ^4^ Department of Biology, University College London, 4, Stephenson Way, NW1 2HE, London, United Kingdom, mathieu.joron@ed.ac.uk

It is now well known that convergent morphology can evolve via repeated recruitment of the same regulatory genes in different lineages. We here contrast three butterfly species, all classic examples of Mullerian mimicry. We use a genetic linkage map to show that a locus, *Yb*, controlling the presence of a yellow band in geographic races of *H. melpomene* maps precisely to the same location as the locus *Cr*, which has very similar phenotypic effects in its co-mimic *H. erato*. Furthermore, the same genomic location acts as a ‘supergene’ determining multiple, sympatric morphs in a third species, *H. numata*, a species with a very different phenotypic appearance, whose many forms mimic different unrelated ithomiine butterflies in the genus *Melinaea*. Hence, a single locus from the multilocus colour pattern architecture in *H. melpomene* and *H. erato* appears to have gained control of the entire wing-pattern variability in *H. numata*, presumably as a result of selection for mimetic ‘supergene’ polymorphism without intermediates. Our results imply that a conserved, yet relatively unconstrained mechanism underlying pattern switching can affect mimicry in radically different ways. We also show that adaptive evolution, both convergent and diversifying, can occur by the repeated involvement of the same genomic regions.

# Research article: Isolation and functional characterization of desaturases of *Manduca sexta*

**DOI:** 10.1673/031.007.2938

**Published:** 2007-05-04

**Authors:** P. Jostova, A. Svatos, Iva Pichova

**Affiliations:** ^1^ Institute of Organic Chemistry and Biochemistry, Academy of Sciences of the Czech Republic, Prague, Czech Republic; ^2^ Max-Planck Institute for Chemical Ecology, Jena, Germany. jostovap@uochb.cas.cz

Desaturases are ubiquitous enzymes that introduce double bonds into a fatty acyl chain. Specific desaturases from female moth abdominal glands are concerned in a pheromone biosynthetic pathway and influence the communication of insect. We have used *Manduca sexta*, a tobacco hornworm, as a model organism for study of pheromone-specific desaturases. Since a number of different pheromones are produced by *M. sexta*, we suppose that several pheromone-specific desaturases are involved in their production. To determine presence and specificity of desaturases, which participate in communication of *M. sexta*, we isolated RNA from a pheromone gland, prepared cDNA library and used it as a template for PCRs. Isolation of the genes encoding desaturases of *Manduca sexta* was performed using a set of degenerated primers recognizing histidine-rich sequence motifs conserved in acyl-CoA desaturases of different organisms. The 3′ and 5′ ends of the gene were obtained by screening of cDNA library by PCR using gene specific primers. For functional expression the whole gene encoding desaturase was subcloned into pYES.2 plasmid (Invitrogen) and transformed into *Saccharomyces cerevisiae*. The products of desaturation were identified by GC-MS. So far we have characterized pheromone specific Δ11Z-desaturase. The phylogeny tree of lepidopteran desaturases revealed smallest divergence with a desaturase from *Bombyx mori* that is bifunctional and forms a conjugated diene. The possible conjugating activity of Δ11Z-desaturase is now under investigation. This work was supported by the Czech Science Foundation (Grant No. 204/03/H066).

# Research article: Positional cloning of *nsd*-*2*, a densovirus-resistance gene in *Bombyx mori*

**DOI:** 10.1673/031.007.2939

**Published:** 2007-05-04

**Authors:** K. Kadono-Okuda, K. Ito, J. Nohata, K. Yamamoto, M. Sasanuma, S. Sasanuma, R. Eguchi, W. Hara, K. Mita

**Affiliations:** Division of Insect Sciences, National Institute of Agrobiological Sciences, Tsukuba, 305–8634, Japan, kadono@affrc.go.jp

*Bombyx mori* densovirus (BmDNV) multiplies in the columnar cell nuclei of the midgut epithelia of the silkworm, *Bombyx mori*. It is classified into two species, DNV-1 and DNV-2 based on their symptoms, serological characters, genome structures and sequences. Some silkworm strains were identified as resistant against DNV-1 and/or DNV-2. In such strains the response reflects non-susceptibility rather than resistance because even high dose of inoculum does not affect their survival rate. So far four non-susceptibility genes have been reported, namely; *nsd*-*1* (L21-8.3), *Nid*-*1* (L17-31.1), *nsd*-*2* (L17-24.5) and *nsd*-*Z* (L15-50.7 and 30.0 cM from apical ends, respectively). However, none of them have yet been isolated as responsible genes. Studies on these genes are useful in understanding the mechanism of non-susceptibility against the viral invasion and multiplication and the functions of the genes by introducing them into the silkworm or cultured cell lines. We have identified four EST markers closely linked with *nsd*-*2*. By taking advantage of *Bombyx* genome information, positional cloning had been performed by BAC-contig construction and linkage analysis of the virus-selected BC1 populations. Further linkage analysis has narrowed the candidate region to 500 kb. We have succeeded in identifying the candidate gene for *nsd*-*2* in this region and examined its tissue, developmental and strain specificities by RT-PCR. Identification of *nsd*-*2* gene leads to the acceleration of the virus research by establishing transgenic cultured cell lines hypersensitive to the virus, in addition to the direct marker-assisted breeding of *Bombyx* strains resistant to DNV-2.

# Research article: Production of hexokinase and anti-human transferrin antibody for clinical diagnostic reagent using transgenic silkworm

**DOI:** 10.1673/031.007.2940

**Published:** 2007-05-04

**Authors:** I. Kiyokawa, I. Kobayashi, K. Uchino, H. Sezutsu, T. Kanda, T. Tamura, T. Miura, T. Ohashi, K. Katayama

**Affiliations:** ^1^ Biochemical Laboratory, Nitto Boseki Co., Ltd. Koriyama 963–8061, Japan; ^2^ Transgenic Silkworm Research Center, National Institute of Agrobiological Sciences, Tsukuba, 305–8634, Japan. KIYOKAWAI@jn.nittobo.co.jp

Recently, recombinant proteins can be used for medical fields, like medicine or clinical diagnostic reagent. The proteins, including antibody, retained native function are especially useful. Silkworm possessed the silk gland that has very high ability to produce and secret proteins. Therefore, the silkworm is thought to be the most suitable insect for the production of recombinant proteins. We successfully produced yeast hexokinase and mouse anti human trasferrin antibody to use diagnostic reagent with transgenic silkworms. The assessment of the produced proteins showed that they retained their functional specificity. The details of our experiments are as follows. [I] Expression of recombinant yeast hexokinase (rHexokinase) using transgenic silkworm. We produced yeast hexokinase, which is used for the ingredient of clinical diagnostic reagent to determine the level of glucose in the patient blood, in the transgenic silkworm. The transgenic silkworm was constructed using transposon *piggyBac* as a vector and the expression of the hexokinase gene was performed using *GAL4*/*UAS* system. The *UAS*-*HXK* gene was constructed by inserting the fusion gene of fibroin L chain cDNA and the hexokinase gene with the tag sequence for purification into the downstream of *UAS*. The gene was transferred to the inverted repeat of *piggyBac* and injected into the silkworm eggs. The strain was mated with the silkworm possessed the *GAL4* gene under the control of fibroin L chain promoter (Imamura et al., 2003). The silkworms with the two genes were detected by PCR with the primers for *GAL4* and HXK genes. Then, we purified the rHexokinase by homogenizing the silk glands of the 5th instar larvae. The purified rHexokinase was detected by SDS-PAGE and its enzyme activity was confirmed in the purified fraction. [II] The Transgenic Silkworm Producing Mouse Antibodies Against Human Transferrin. We first constructed the plasmid vector pUASFvaTf for the production of single chain variable fragment antibody against human transferring (scFvaTf) in the silkworm. The *GAL4*/*UAS* expression system was also used. The vector was constructed by inserting the gene downstream of *UAS* and the *UASFvaTf* strain was mated with the strain with the *GAL4* gene under the control of sericin 1 gene promoter (Tamura et al., 2004). Western Blotting for the detection of the target protein was performed as follows. First, *SerGAL4*/*3XP3DsRed* and *UASFvaTr*/*3XP3GFP* strains were mated and the eggs with the both genes were selected by observing the stemma using fluorescent stereo microscope equipped with DsRed and GFP filters. The silkworms with the both genes were reared and the silk glands were dissected at the day of spinning, next day and two days later. The recombinant scFvaTf was extracted from the silk gland homogenate with lyses buffer. The extracted samples were undertaken SDS-PAGE, transfer to PVDF membrane and reaction with rabbit anti mouse Ig-HRP labeled antibody. The result of Western Blotting identified the protein with the molecular weight of the predicted size, indicating the production of the recombinant scFvaTf. The specificity of the produced antibody against the antigen was determined by ELISA. The silk gland extract of the transgenic silkworm was first reacted with human transferrin-coating micro titer plate. Then, transferrin - scFvaTf HRP-linked rabbit anti- mouse immunoglobulin complex was formed using HRP-linked rabbit anti-mouse immunoglobulin antibody. Finally, the reaction with substrate system for ELISA was performed. The result showed that the amount of reacted materials was increased in proportion to the extract of the silk gland, indicating that the produced antibody possessed activity as an antibody against the transferrin.

# Research article: Development of inducible RNAi using by *GAL4*/*UAS* system in the transgenic silkworm

**DOI:** 10.1673/031.007.2941

**Published:** 2007-05-04

**Authors:** I. Kobayashi, H. Sezutsu, K. Uchino, T. Tamura

**Affiliations:** Transgenic Silkworm Research Center, National Institute of Agrobiological Science, Owashi 1–2, Tsukuba, Ibaraki 305–8634, Japan. ttamura@affrc.go.jp

RNA interference (RNAi) is a powerful tool to analyze the gene function in a variety of organisms. We previously reported that an injection of dsRNA into the early developmental embryo efficiently works to repress the target gene. However, the method is only useful to study the gene expressed at early stage of development. We are interested to develop the method working the gene at late stage of development. The inducible RNAi using the transgenic organism was known to be useful to analyze the function of the late genes. In the experiment, we attempted to apply the method in the silkworm. We first constructed a new vector to transcribe the RNA with hairpin structure of the target genes in the transgenic silkworm. The vector contains a marker gene of *3XP3GFP* and the silkworm cytoplasmic actin intron to transport the transcribed RNA to cytoplasm. The introduced gene is placed under the control of *UAS* in opposite direction and driven by GAL4. Using the vector, we constructed three different constructs corresponding to the cDNA sequences of three different genes. We used the two mutant genes, *white egg 3* (*w3*) and *Gialo Ascoli translucent* (og). It was known that *w3* codes the *Bombyx white* gene (*Bmwh4*) and *og* corresponds to *molybdenum cofactor sulfuruse* gene (*MoCo*). The mutation of *Bmwh4* produces the white egg and eyes, and translucent integument. The mutation of *MoCo* caused the translucent integument at larval stage. In addition, we constructed the vector to repress the expression of *fibroin H chain* gene (*fibH*). The transgenic strain having each RNAi constructs (*UAS*-*Bmwh*-*IR, UAS*-*MoCo*-*IR* and *UAS*-*fibH*-IR) was generated by the ordinal method for germ line-transformation using the transposon *piggyBac* and mated with *GAL4* strains. When *UAS*-*Bmwh*-*IR* strain was mated with *GaL4* line with a promoter of *Bombyx* cytoplasmic actin gene (*A3GAL4*), the part of the integument of the larvae with the both genes became translucent and formed mosaic pattern mixed with the translucent and opaque regions. The area of the translucent integument was increased when the introduced gene present in homozygous. The content of uric acid in the integument in the larvae with the both genes was lowered compared to the normal strain. However, the morphological change was not observed when the *UAS*-*MoCo*-*IR* gene was expressed by *A3-GAL4*. We interpreted the result that the repression of the expression of *MoCo* is not enough to induce the morphological change in the silkworm. When the larva having the *UAS*-*flbH*-*IR* sequence and the *GAL4* gene with the promoter *of fibroin L chain* gene was reared, the cocoon shell weight was lighter than that of the control and the reduction of the fibroin H chain mRNA was observed. We concluded from the results that the reduction of the mRNA of the target gene occurred by our method and that the inducible RNAi method developed in our experiment is useful for the study of gene function of the silkworm although the effect of repression is limited.

# Research article: Insect photoperiodism and circadian clocks: expression patterns of genes *period, timeless, cycle* and *cryptochrome* in *Sesamia nonagrioides* (Lepidoptera: Noctuidae)

**DOI:** 10.1673/031.007.2942

**Published:** 2007-05-04

**Authors:** A. Kourti, T. Gkouvitsas

**Affiliations:** Department of Agricultural Biotechnology, Agricultural University of Athens, Iera Odos 75, 11855, Athens, Greece. akourti@aua.gr

Photoperiodic clocks allow organisms to predict the coming season. In insects, the seasonal adaptive response mainly takes the form of diapause. Though our knowledge of the molecular details of the circadian clock has advanced rapidly, the functional elements of the photoperiodic clock remain largely unknown. As a step to approach this issue, we report here the sequences and expression paterns of *period* (*per*), *timeless* (*tim*), *cycle* (*cyc*) and *cryptochrome* (*cry*) mRNAs in the corn stalk borer *Sesamia nonagrioides* (Lepidoptera: Noctuidae). Nucleotide and deduced amino acid sequences of the genes in *S. nonagrioides* show high similarity to homologous genes in other insects that have been investigated. *S. nonagrioides*. The PERIOD has in PAS domain the pentapeptide GTPEK-like sequence. A diel rhythmicity of *per* and *tim* mRNA abundance was detected in the larvae heads (peak during scotophase), while *cry* and *cyc* mRNA abundance remained fairly constant throughout. The abundance of *cyc* mRNA was quaite low when compared to *per, tim* and *cry* mRNA. Rearing temperature affected the amount of *per* and *tim* mRNAs: abundance of *per* mRNA increased at 20 °C when compared to 25 °C, but that of *tim* mRNA decreased. Photoperiod influenced the expression patterns of *per* and *tim* mRNA: the peak of *per* mRNA expression shifted in concert with onset of the scotophase, while a shift in *tim* mRNA expression was less pronounced. The amplitude of *tim* mRNA was severely dampened under long daylength, but that of *per* mRNA was not affected. These distinct patterns of expression suggest that this information could be used to determine photoperiodic responses such as diapause. The project is co-funded by european social fund (75%) and national resources (25%) - O.P. “EDUCATION” I.

# Research article: Cellular response to DNA double strand breaks (DSBs) in cultured silkworm cells

**DOI:** 10.1673/031.007.2943

**Published:** 2007-05-04

**Authors:** T. Kusakabe, H. Mon, M. Takahashi, J.M. Lee, Y. Kawaguchi

**Affiliations:** Laboratory of Silkworm Science, Kyushu University Graduate School of Bioresource and Bioenvironmental Sciences, Hakozaki 6–10–1, Fukuoka 812–8581, Japan kusakabe@agr.kyushu-u.ac.jp

DNA double-strand breaks (DSBs), caused by a variety of endogenous and exogenous DNA-damaging agents, are one of the most serious forms of DNA damage that occurs in a cell's genome, and thus preserving genomic integrity in all organisms is an essential process. Compared with other organisms, insects are believed to exhibit a pronounced resistance to IR. Cells from insects also retain this pronounced intrinsic resistance to IR even when proliferating *in vitro*. These findings point out the presence of an inherent cellular basis for the resistance and lead to the hypothesis that insect possess the specific mechanisms for protecting themselves from DNA damage induced by IR. In this study, we focused on the cell cycle regulation response to DSB by γ- and UV- irradiation in the cultured silkworm cells, BmN4. Upon the serious DNA damage, BmN4 cells arrested cell cycle at the G2/M phase by γ-irradiation, but not at G1 or S phase. In constrast to the mammalian cells, the apoptic cell death after the irradiation could not be detected in the BmN4. We are analyzing the activation of sensor proteins after DSB induction in the silkworm cells.

# Research article: The interaction of the *Cotesia congregata* bracovirus CcV1 protein with *Manduca sexta* hemolin

**DOI:** 10.1673/031.007.2944

**Published:** 2007-05-04

**Authors:** V. Labropoulou, V. Douris, D. Stefanou, C. Magkrioti, E. Andronopoulou, L. Swevers, K. Iatrou

**Affiliations:** Insect Molecular Genetics and Biotechnology Group, Institute of Biology, National Center for Scientific Research “Demokritos”, PO Box 60228, GR 153 10 Aghia Paraskevi (Athens), Greece. beslabro@mail.demokritos.gr

During parasitization, endosymbiotic polydnaviruses (PDVs) associated with the female hymenopteran parasitoid wasp *Cotesia congregata* are injected into the lepidopteran host *Manduca sexta* together with the embryos of the parasitoid. PDVs do not replicate in the lepidopteran host but the expression of their genes in the host cells causes physiological alterations and suppression of the host's immune system. To identify hemocyte proteins of *Manduca sexta* that interact with PDV proteins of *C. congregata*, we employed the yeast two-hybrid assay as a screening tool for a cDNA (prey) library made from non-immunized *M. sexta* hemocytes against two *C. congregata* PDV-encoded proteins (baits), CcV1 and CcVank1. The screening process resulted in the detection of several putatively interacting hemocyte proteins. Sequence analysis of the hemocyte clones whose products interact with CcV1 in yeast showed that the clones encode hemolin and a serine protease-like protein (SPH2), while lebocin, hemolin, scolexin, transferrin and serpin1 were identified as interacting preys for the CcVank1 bait. Most of the putatively interacting proteins participate in the immune response of the host and all interactions have been confirmed to occur in yeast cells. To characterize further the interaction between CcV1 (a secreted protein with predicted and observed molecular weights of 53 kDa and 85kDa, respectively, containing coiled coil, syndecan and proline rich domains and several O- and N-glcosylation sites) and hemolin (a major immune response protein of M. sexta), the two proteins were expressed as epitope-tagged forms in lepidopteran cells. Co-immunoprecipitation and chemical cross-linking assays employing the recombinant proteins confirmed the deduced interactions. Further functional studies suggest that CcV1 can alter some of the hemolin's functions including its capacity to cause agglutination of E. coli bacteria and its binding to immobilized lipopolysaccharides. Furthermore, CcV1 was shown to be actively uptaken by BTI-TN-5B1-4 (HighFive™) cultured cells in vitro and Bombyx mori hemocytes upon in vivo injection into larvae. Future studies aim at the further elucidation of the role of CcV1 in the immune suppression response by PDVs, more specifically its putative inhibition of hemolin and SPH2 function. Also the possible function of CcVank1 in the inhibition of the NFχB-mediated transcriptional response to infection will be evaluated.

# Research article: Analysis of the first Banchinae polydnavirus genome sequence: A comparison with Bracovirus and Ichnovirus genomes

**DOI:** 10.1673/031.007.2945

**Published:** 2007-05-04

**Authors:** R. Lapointe, K. Tanaka, W. Barney, J. Whitfield, J. Banks, C. Béliveau, D. Stoltz, B.A. Webb, M. Cusson

**Affiliations:** ^1^ Laurentian Forestry Centre, Natural Resources Canada, Canadian Forest Service, 1055 rue du PEPS, Sainte-Foy, QC, G1V 4C7, Canada; ^2^ Dep. of Entomology, University of Kentucky, Lexington, KY, 40546–0091, USA; ^3^ Dept. of Entomology, University of Illinois at Urbana-Champaign, Urbana, IL, 61801, USA; ^4^ Dept. of Microbiology and Immunology, Dalhousie University, Halifax, NS, B3H 4H7, Canada. cusson@cfl.forestry.ca

To overcome physiological barriers encountered in their hosts, many ichneumonid and braconid endoparasitoids transmit a polydnavirus (PDV) to caterpillars during parasitization. The viral entities carried by wasps of each family have been assigned to separate taxa, ichnovirus (IV) and bracovirus (BV), respectively. Although they are believed to have no direct common ancestor, IVs and BVs share several features, including a segmented dsDNA genome and wasp-restricted replication from chromosomally integrated proviral DNA; however, the proteins encoded by their genomes appear largely unrelated. All IVs characterized to date are found in species of the ichneumonid subfamily Campopleginae. As a consequence, little is know about the PDV particles carried by wasps of the subfamily Banchinae, the only other ichneumonid taxon known to carry these unusual viruses. Here we report on the genome sequence analysis of a PDV transmitted to the spruce budworm, *Choristoneura fumiferana*, by the banchine parasitoid *Glypta famiferanae*. With an aggregate genome size of ∼290 kb and 105 genome segments, this virus displays a degree of genome segmentation that far exceeds levels reported for BVs and campoplegine IVs. The size range of its genome segments (1.5 – 5.2 kb, median 2.7 kb) is also significantly lower than that which is observed in these latter two groups. As reported for other PDVs, the predicted ORFs of this virus cluster into gene families, including the viral ankyrin (*ank*) family, but phylogenetic analysis suggests that the *ank* genes of the Gf virus are more closely related to those predicted from BV than IV genomes. With 23 representatives, protein tyrosine phosphatases (PTPs) make up the largest gene family of this virus. PTPs are also very abundant in BV genomes but those of the Gf virus and BVs form distinct phylogenetic clusters. We believe that the unique genomic features of the present banchine virus, along with the previously reported morphological singularities of its virions, call for the establishment of a new PDV taxon.

# Research article: Chromosomal conservation in Lepidoptera: Synteny versus collinearity

**DOI:** 10.1673/031.007.2946

**Published:** 2007-05-04

**Authors:** Siu Fai Lee, David G. Heckel

**Affiliations:** ^1^ Bio21 Institute, CESAR, Dept. Genetics, University of Melbourne, Parkville, Victoria 3010, Australia; ^2^ Max Planck Institute for Chemical Ecology, Department of Entomology, Hans-Knoell-Str. 8, Jena D-07745, Germany. s.lee32@pgrad.unimelb.edu.au

To determine the degree of chromosomal conservation in Lepidoptera, we mapped a core set of 60 anchor loci in the cotton bollworm *Helicoverpa armigera* and the European corn borer *Ostrinia nubilalis*. Most of the anchors were ribosomal protein genes; which are generally single-copy in insect genomes and have highly conserved sequences on the amino acid level. The degree of DNA sequence similarity is high enough for cDNA probes from one species to give hybridization signals on Southern blots made from DNA of other lepidopteran species; while there is sufficient polymorphism in introns and flanking UTR sequences for mapping. We compared our results with available information from *Bombyx mori*. We found a high degree of synteny (co-occurrence of the same anchors on the same chromosome), but not always the same collinearity (order of the anchors within the chromosome). The most frequent violations of synteny involved gene duplication of ribosomal protein genes, which seem to be lineage-specific. In comparing species with different chromosome numbers, there must be at least some violations of synteny. One large linkage group showed some gene order differences among the species, indicating that some intra-chromosomal rearrangements have taken place. Thus synteny does not always imply collinearity. Currently, the density of marker loci limits our ability to test this on the smaller chromosomes; we suggest methods to overcome this limitation.

# Research article: Role of the RNA triphosphatase activity of baculovirus LEF-4 in late gene expression and viral replication

**DOI:** 10.1673/031.007.2947

**Published:** 2007-05-04

**Authors:** Yi Li, Linda A. Guarino

**Affiliations:** Department of Entomology, Texas A&M University, College Station, TX 77843, U.S.A. lguarino@tamu.edu

Baculovirus encode an RNA polymerase that transcribes viral late genes. This 4 subunit complex also has promoter recognition, mRNA capping, and polyadenylation activities. The capping activity has been mapped to the LEF4 subunit, which has guanyltransferase (GTase) and RNA triphosphatase activities (RTPase). Transient expression assays, as well as the isolation of a temperature sensitive virus, indicate that the LEF4 subunit is required for late gene expression and virus replication. In addition, construction of LEF-4 knockout virus indicates that the GTase function is required for viral replication. Here we report on the requirement for RTPase activity. To address the role of baculovirus RTPase activity, site specific mutations were constructed that destroyed RTPase activity in LEF4 without affecting GTase activity. The mutant constructs were tested for the ability to support late gene expression by transient assay, and also reconstructed into whole virus to determine levels of virus replication in the absence of RTPase activity. Transient expression assays revealed that RTPase activity was dispensable as reporter levels were only slightly decreased as compared to wildtype protein. Results with mutant virus were similar. No viable virus was recovered in the absence of LEF4 or with a LEF4 mutant containing a critical substitution within the GTase domain. RTPase mutants, however, were viable and, furthermore, the yields of infectious virus were indistinguishable from wildtype. The lack of a requirement for RTPase function was surprising as this enzyme activity is required for the formation of an authentic cap structure which is required for efficient translation. This suggests that either the host enzyme or the viral protein PTP, which also has RNA triphosphatase activity, can substitute for LEF4.

# Research article: Genetic mapping and analysis of quantitative trait loci affecting cocoon quality in the silkworm, *Bombyx mori*

**DOI:** 10.1673/031.007.2948

**Published:** 2007-05-04

**Authors:** Muwang Li, Minhui Li, Qiuhong Guo, Xuexia Miao, Chengxiang Hou, Hongxuan Lin, Yongping Huang

**Affiliations:** ^1^ Institute of Plant Physiology and Ecology, Shanghai Institutes for Biological Sciences, Chinese Academy of Sciences; Shanghai, 200032, P.R. China; ^2^ Sericultural Research Institute, Chinese Academy of Agricultural Sciences, Zhenjiang, 212018, P.R. China, mwli@sibs.ac.cn

A backcrossed population (BC1) was derived from a cross between Jingsong and Lan10 SSR technique was employed for mapping QTLs. The QTLs for the whole cocoon weight, cocoon shell weight, ratio of cocoon shell, weight of pupae were analyzed, and 7 QTLs were detected based on the constructed linkage map. Three QTLs for whole cocoon weight, pupal weight and cocoon shell weight were localized on linkage group 1 and 23 respectively, and 2 of the QTLs were on the same locus, the other one was on different locus of group 23. Two QTLs for cocoon shell ratio were localized on linkage group 18 and 19. These results provide an important base for the marker assisted breeding of the silkworm.

# Research article: Development and analysis of an EST database from the moth *Choristoneura fumiferana*

**DOI:** 10.1673/031.007.2949

**Published:** 2007-05-04

**Authors:** Lan Li, Sichun Zheng, Tim Ladd, Dayu Zhang, Deborah Buhlers, Peter J. Krell, Basil M. Arif, Arthur Retnakaran, Qili Feng, Daniel Doucet

**Affiliations:** ^1^ Great Lakes Forestry Centre, Canadian Forest Service, 1219 Queen Street East, Sault Ste. Marie, Ontario, Canada P6A 2E5; ^2^ Department of Molecular and Cellular Biology, University of Guelph, Guelph, ON, Canada, N1G 2W1; ^3^ College of Life Sciences, South China Normal University, Guangzhou, China. Dan.Doucet@NRCan.gc.ca

An expression sequence tag resource from the spruce budworm, *Choristoneura fumiferana*, has been developed to expand the knowledge on basic biological processes in this insect such as development and immune defense. Close to 50,000 ESTs from six normalized cDNA libraries (epidermis, fat body and midgut from larvae molting from 5th to 6th instar stages, embryo, diapausing second instar and adult head) were analyzed. The ESTs have been assembled into approximately 8600 unigenes. A data pipeline of generation and analysis of EST data has been developed, including raw sequences screening, sequence de-contamination, trimming, assembling, annotation and gene ontology classification. Annotation was conducted against the protein database Swiss-Prot/TrEMBL and the GenBank of NCBI. The unique sequences were classified according to their molecular function, cellular location, and biological process by using the Gene Ontology classification system. A spruce budworm EST database containing high quality EST that are vector-free and at least 200bp in length has been established and can be searched by BLAST and Keyword. The relational database comprises information of clone ID, gene description, EST numbers, GO classification. A further bioinformatics analysis of transcripts of the spruce budworm is based on the annotated data resources.

# Research article: Prostaglandin signaling and cAMP production control the rate of follicle progression from vitellogenesis to choriogenesis during silkmoth oogenesis

**DOI:** 10.1673/031.007.2950

**Published:** 2007-05-04

**Authors:** Ednildo Machado, Luc Swevers, Kostas Iatrou

**Affiliations:** ^1^ Instituo de Biofísica do Rio de Janeiro da UFRJ, Brazil; ^2^ Institute of Biology, NCSR “Demokritos”, Athens, Greece. swevers@bio.demokritos.gr, iatrou@bio.demokritos.gr

*In vitro* cultures have shown that follicle development in the ovary of the silkmoth, *Bombyx mori*, occurs autonomously from early stages at mid-vitellogenesis until the completion of choriogenesis. Experiments measuring the kinetics of follicle development indicate that follicles enter and complete choriogenesis with similar rates in *in vitro* cultures as *in vivo*. While the above experiments suggest that follicles develop according to an endogenous developmental program that does not require any additional factors from tissues outside the ovariole, they do not exclude the possibility of autocrine/paracrine signaling among follicles of different stages within the same ovariole to coordinate the simultaneous progression of follicle development. Prostaglandins are oxygenated metabolites of the C20 polyunsaturated fatty acid arachidonic acid that essentially function as paracrine mediators in a variety of physiological processes such as tissue differentiation, reproduction and immunity. Recent experiments have shown that prostaglandins play a critical role in the regulation of the transition from vitellogenesis to choriogenesis. Ovarioles cultured in the presence of aspirin and indomethacin, potent inhibitors of cyclo-oxygenase, the rate-limiting enzyme involved in prostaglandin biosynthesis, accumulate follicles at the stages of late vitellogenesis indicating that a developmental arrest or delay occurs prior to the initiation of choriogenesis. These findings are corroborated by the detection by Western blot analysis of high levels of cyclo-oxygenase protein at the vitellogenesis-choriogenesis transition. The developmental delay/arrest induced by cyclo-oxygenase inhibitors can be rescued by exogenous addition of prostaglandins or cAMP, invoking a model in which the local production of prostaglandins induces cAMP signaling in the follicles and ensuing progression towards choriogenesis. Our results indicate a major role for paracrine or autocrine signaling by prostaglandins to regulate the transition from vitellogenesis to choriogenesis during silkmoth oogenesis.

# Research article: On the evolution of insect wings

**DOI:** 10.1673/031.007.2951

**Published:** 2007-05-04

**Authors:** Kalpana Makhijani, V Bharathi, Ramakrishnan Kannan, L S Shashidhara

**Affiliations:** Center for Cellular and Molecular Biology, Uppal Road, 500007 Hyderabad, India. shashi@ccmb.res.in

Suppression of wing fate and specification of haltere fate in *Drosophila* by the homeotic gene *Ultrabithorax* (*Ubx*) is a classical example of Hox regulation of serial homology (Lewis EB. 1978. *Nature* 276: 565–570), which has served as a paradigm for understanding homeotic gene function. The differential development of wing and haltere constitutes a good genetic system to study cell fate determination at different levels such as growth, cell shape, size and its biochemical and physiological properties. They also represent the evolutionary trend that has established the differences between fore and hind wings in insects. Interestingly, Ubx protein itself has not evolved amongst the diverse insect groups, although there are significant differences in Ubx sequences between *Drosophila* and crustacean Arthropods (Galant R, Carroll SB. 2002. *Nature* 415: 910–3; Ronshaugen, M, McGinnis N, McGinnis W. 2002. *Nature* 415: 914–7). Nevertheless, over-expression of Ubx derived from a non-winged arthropod, such as Onychophora, is sufficient to induce wing-to-haltere transformations in *Drosophila* (Grenier JK, Carroll SB. 2000. *Proc. Natl. Acad. Sci*., *USA* 97: 704–709). This suggests that in the dipteran lineage, certain wing patterning genes have come under the regulation of Ubx (Weatherbee et al. 1999. *Current Biology* 9: 109–15). One way to approach the mechanism of *Ubx* function is to reconstruct a wing appendage in the third thoracic segment without altering the patterns/levels of *Ubx* expression. This necessitates identification of genes that are differentially expressed between wing and haltere discs and reverse-engineer the expression of one or more of those genes during haltere development. We have employed several complementary approaches to identify targets of *Ubx* (Shashidhara LS et al. 1999. *Developmental Biology* 212: 491–502; Mohit P, Bajpai R, Shashidhara LS. 2003. *Development* 130: 1537–47; Mohit P et al. 2007. *Developmental Biology* (in press); Bajpai et al. 2004. *Mechanism of Development* (*GEP*). 5: 113–121). Several observations from our lab and from others suggest that *Ubx* down regulates activities of the signaling centers, such as anterior-posterior (A/P) and dorso-ventral (D/V) organizers, to specify haltere fate. Downstream of A/P and D/V signaling pathways, *Ubx* negatively regulates EGFR pathway to repress the development of wing-specific features, such as veins and sensory organs of the wing-margin. Our observations suggest a mechanism in which *Ubx* dampens organizing activities of compartment boundaries and thereby, repress the wing fate. We discuss a possible a mechanism in which fine tuning of key signaling pathways may have led to the evolution of halteres in dipteran insects. Our future plan in this direction involves identifying those genes that have come under the influence of *Ubx* specifically during dipteran evolution. This involves extensive bioinformatics analyses and identifying direct targets of Ubx by Chromatin-immunoprecipitation (ChIP) from different insect groups such as *Apis*, butterflies, silkworm, *Tribolium*, mosquito and different species of *Drosophila*.

# Research article: Using *Junonia coenia*, the buckeye butterfly, as a model system to study the evolutionary developmental genetics of lepidopteran color patterns

**DOI:** 10.1673/031.007.2952

**Published:** 2007-05-04

**Authors:** J.M. Marcus

**Affiliations:** Department of Biology, Western Kentucky University, Bowling Green, KY USA. jeffrey.marcus@wku.edu

Butterfly color patterns are an important case study for research at the interface between development and evolution. Color pattern traits often have clearly identifiable functions in the ecology of butterflies, playing roles in activities such as mate selection, avoidance of predation, and thermoregulation. We also have a growing understanding of the genetic interactions that are required to produce color patterns. The ability to understand a single phenotype at so many different levels of biological organization, combined with the fact that butterfly color patterns are structurally very simple, existing in only two dimensions, has made color patterns an attractive system for both experimental and theoretical biologists. I will describe progress we have made in developing genetic, genomic, developmental, phylogenetic, and computational tools to study color pattern evolution in the common buckeye butterfly, *Junonia coenia*. Our experiments in germ line transformation, AFLP-based genome mapping, immunohistochemistry, molecular phylogenetics, population genetics, and computer simulation of genetic interactions have begun to demonstrate the synergistic power of studying a single model species from many different complementary perspectives. This approach not only facilitates the production of better answers to research questions, it also helps us ask better questions.

# Research article: Chlorophyllid A binding protein, a new large fluorescent lipocalin (Polycalin) from the midgut of *Bombyx mori* L

**DOI:** 10.1673/031.007.2953

**Published:** 2007-05-04

**Authors:** Bernard Mauchamp, Corinne Royer, Annie Garel, Audrey Jalabert, Martine Da Rocha, Anne-Marie Grenier, Valérie Labas, Joëlle Vinh, Kazuei Mita, Keiko Kadono-Okuda, Gérard Chavancy

**Affiliations:** ^a^ Unité Nationale Séricicole/INRA, 25 quai JJ. Rousseau, 69350 La Mulatière, France; ^b^ Lab. Neurobiologie ESPCI, 10 rue Vauquelin 75005 Paris France; ^c^ Genome Research Dep. NIAS, 1–2 Owashi, Tsukuba, Ibaraki 305–8634 Japan; ^d^ Centre de Génétique Moléculaire et Cellulaire,CNRS,69100 Villeurbanne, France; ^e^ present address: Unit. Physiol. Comportement, UMR 6175 INRA 37380 Nouzilly Fr. Royer@cismsun.univ-lyoni.fr

The midgut is the gate for infection of silkworms after oral ingestion of BmNP virus. A protein previously called red fluorescent protein was described as possessing antiviral activity against BmNPV, but no molecular characteristics were available. We focussed our studies on the determination of its amino acid sequence and the nucleotid sequence of its gene. Protein after extraction was purified on 2D gel electrophoresis; the fluorescent spot was trypsinized and obtained peptides analyzed by mass spectrometry. The amino acid sequence of several peptides was determined. Some of these sequences were blasted in *B. mori* EST. From total RNA and RT-PCR with specific primers we established the total cDNA sequence of the protein, then its deduced amino acid sequence. The cDNA encoded a protein of 2721 amino acids (302kDa). In addition, WGS gene bank blast listed the contigs BAABO1026110, AADKO1000800 and AAKO1004726.1, and obtain information on the structure of the genomic sequence characterized by a large number of introns (45) and exons (46). This protein belongs to the lipocalin family, but its size and its arrangement are very new. This protein has 15 lipocalin structures each one characterized by a well-conserved tertiary structure. We introduce the notion of polycalin (pentadecacalin) and called it Chlorophyllid A binding protein since the ligand is chlorophyllid, the product of degradation of chlorophyll by chlorophyllase.

# Research article: Cloning and expression of manganese superoxide dismutase of the silkworm, *Bombyx* *mori* by Bac-to-Bac/BmNPV Baculovirus expression system

**DOI:** 10.1673/031.007.2954

**Published:** 2007-05-04

**Authors:** Yungen Miao, Wanfu Yue, Xinghua Li, Xiaofeng Wu

**Affiliations:** College of Animal Sciences, Zhejiang University, Hangzhou 310029, China, miaoyg@zju.edu.cn

Superoxide dismutase (SODs) are metalloenzymes that catalyze the dismutation of the superoxide anion to molecular oxygen and hydrogen peroxide and thus form a crucial part of the cellular antioxidant defense mechanism. On other hand, the silkworm has become an ideal multicellular eukaryotic model system for basic research. The major advantages of expressing foreign genes in silkworm larvae are the low cost of feeding, the extremely high levels of expression achievable compared with expression in cell lines and increased safety because the baculovirus is noninfectious to vertebrates. In this paper, we used the total fat body RNA of silkworm, *Bombyx mori* L to clone and sequence a 648 bp Mn-SOD cDNA fragment through RT-PCR. Furthermore a newly established Bac-to-Bac/BmNPV Baculovirus expression system was used to overexpress the recombinant Mn-SOD enzyme in silkworm larvae. The hemolymph was collected from the infected larvae on 96 h post-infection and subjected to a 12% SDS-PAGE and western blotting. A 18.0 kDa-protein was visualized after rBacmid/BmNPV/SOD infection. The SOD enzyme activity was determined with a tetrazolium salt for detection of superoxide radicals generated by xanthine and xanthine oxidase and its peak appeared in 96 hours post-infection with 2.7 times of the control larvae. The availability of large quantities of SOD that the silkworm provides should greatly facilitate the future research and testing of this protein for potential application in medicine. The results also showed that the Bac-to-Bac/BmNPV baculovirus expression system is an efficient tool to express the target gene in silkworm larvae, which takes only 7–10 days for generating recombinant baculovirus, compared with the traditional homologous recombination method, which needs at least 40 days for multiple rounds of purification and amplification of viruses.

# Research article: Transgenic pink bollworm and tobacco budworm with piggyBac-like elements present

**DOI:** 10.1673/031.007.2955

**Published:** 2007-05-04

**Authors:** T.A. Miller, Y. Park, X. Ren

**Affiliations:** ^1^ Department of Entomology, University of California, Riverside, CA, 92521, USA; ^2^ Department of Entomology, Kansas State University, Manhattan, KS, 66506, USA; ^3^ Department of Molecular microbiology and Immunology, Johns Hopkins University, Baltimore, MD 21205, USA. thomas.miller@ucr.edu

Active transposable elements can self-replicate and mobilize to cause deleterious or adaptive mutations. The *piggyBac* element, originally discovered in a cell line of the cabbage looper moth, *Trichoplusia ni*, is widely used in transgenesis of insects. *PiggyBac*-*like* elements (PBLE) have been found in pink bollworm, *Pectinophora gossypiella* (Saunders) and tobacco budworm, *Heliothis virescens* (Fabricius). Both active (*HvPLE1*) and inactive (*HvPLE2*) versions were present in tobacco budworm as well as elements hybridizing to a probe based on the original *PiggyBac* element from cabbage looper. Large diversities of PBLE were found among individual tobacco budworm worms in laboratory populations suggesting that they are active in transposition. This activity could explain why genetic transformation of tobacco budworm using a PBLE was putatively successful, but lead to unstable strains in which marker genes were lost by the second generation. If true, one must be aware that tobacco budworm has the ability to mutate via transposable elements to a high degree. The tobacco budworm is a multi-host pest insect able to induce a variety of enzymes depending on specific host plant. Pink bollworm is a microlepidopteran moth with a narrow host range (malvaceous plants such as cotton *Gossypium* spp., okra, *Abelmoschus* spp., and hibiscus, *Hibiscus* spp.) and was genetically transformed with *a piggyBac* element in 1998 and has been reared through dozens of generations in quarantine without loss of markers and out-crossed and back-crossed to improve fitness. We found that pink bollworm also has endogenous PBLEs present. These appear to have little or no effect on stability of transgenics in stark contrast to tobacco budworm.

# Research article: Prospects on the *Bombyx* genome analysis

**DOI:** 10.1673/031.007.2956

**Published:** 2007-05-04

**Authors:** K. Mita, M. Kasahara, S. Sasaki, Y. Nagayasu, T. Yamada, H. Kanamori, N. Namiki, M. Kitagawa, H. Yamashita, Y. Yasukochi, K. Kadono-Okuda, K. Yamamoto, M. Ajimura, G. Rvikumar, M. Shimomura, Y. Nagamura, T. Shin-I, H. Abe, T. Shimada, S. Morishita, T. Sasaki

**Affiliations:** Genome Research Department, National Institute of Agrobiological Sciences, Tsukuba 305–8634, Japan, kmita@nias.affrc.go.jp

In Lepidoptera, the genome information had been quite limited so far. Now the situation changed quickly, since National Institute of Agrobiological Sciences (NIAS) is performing the *Bombyx* genome project including *Bombyx* whole genome shotgun sequencing, cDNA collection and construction of physical map systematically in collaboration with many groups. Also Southwest Agricultural University (SWAU) group has published their *Bombyx* WGS sequence data. The integration of both WGS data has just been launched for a complete *Bombyx* genome sequence, which will provide a powerful basis for the analysis of many biological phenomena to *Bombyx* and other Lepidoptera. A) - EST db: So far, about 164,000 ESTs were collected from 59 cDNA libraries prepared from various tissues of different developmental stages including 9 full-length cDNA libraries. All ESTs are compiled into KAIKObase <http://sgp.dna.affrc.go.jp>, where EST db is combined with genome and map information as the integrated database. B) Linkage map: About 2,000 genetic markers were mapped in 28 linkage groups under the efforts of several groups. Recently, SNP analysis using BAC end sequences was carried out and a high density map of BAC markers is available (Yamamoto et al., 2006). C) - BAC library: Three BAC libraries prepared by different restriction enzymes are available. D) - EST microarray: EST microarray containing 6,000 non-redundant ESTs was available for functional studies. Recently, oligoarray, which is much more powerful than the old 6,000 EST microarray in gene coverage and sensitivity, having 60mers of more than 34,000 clusters from 164K ESTs has been made and available for functional studies. E) - Genomic sequence: NIAS carried out *Bombyx* WGS, whose sequence contig data are public in KAIKObase of NIAS HP. Also SWAU WGS sequence data are included and available in KAIKObase. Integration of both WGS data has been launched from this April, and a complete genome sequence information will be expected.

# Research article: Double-strand break repair by homologous recombination in silkworm cells

**DOI:** 10.1673/031.007.2957

**Published:** 2007-05-04

**Authors:** H. Mon, R. Sugahara, J.M. Lee, Y. Kawaguchi, T. Kusakabe

**Affiliations:** Laboratory of Silkworm Science, Kyushu University Graduate School of Bioresource and Bioenvironmental Sciences, Hakozaki 6–10–1, Fukuoka 812–8581, Japan. mhiro@agr.kyushu-u.ac.jp

Maintenance of genome stability relies on the accurate repair of double-strand breaks (DSBs) that arise during DNA replication or from DNA-damaging agents. Failure to repair such breaks can lead to the introduction of mutations, chromosomal translocations. In eukaryotes, homologous recombination (HR) has emerged as the major mechanism for the error-free homology - directed repair of DSBs. The analysis of radiosensitive yeast mutants has revealed a number of genes involved in HR, which comprise the RAD52 epistasis group. As to insects, it is highly likely that DNA repair systems are more or less different from those in other organisms. To understand the HR repair mechanism, we have isolated RAD51, BRCA2 and paralogs of RAD51 from the silkworm, *Bombyx mori*. RAD51, a homolog of bacterial RecA, plays a central role in homologous DNA recombination, and its activity is controlled by a number of RAD51 cofactors, BRCA2 and RAD51 palarogs. Using yeast two-hybrid analysis, the BRC repeats and carboxy-terminal region of BmBRCA2 can interact with BmRAD51. Furthermore, we detected the interaction between the Rad51C and Rad51D. In addition, we observed the RNAi against BRCA2 mRNA resulted in a decrease of HR efficiencies. These findings might suggest that the existence of an evolutionarily conserved pathway for DSBs repair in *Bombyx mori*.

# Research article: Testing the role of candidate genes in butterfly eyespot development using transgenics

**DOI:** 10.1673/031.007.2958

**Published:** 2007-05-04

**Authors:** Antónia Monteiro, Bin Chen, Diane Ramos, Firdous Kamal, Gary Glaser, Steven Stockslager

**Affiliations:** ^1^ Biological Sciences, University at Buffalo, Buffalo, NY 14260, USA; ^2^ Ecology and Evolutionary Biology, Yale University, 165 Prospect Street, New Haven, CT 06520-8106. monteiro@buffalo.edu

Eyespots with concentric rings of colored scales are complex structures that appear in a variety of lepidopteran families. Moths and butterflies share similar gene expression patterns in their eyespot centers, suggesting that a conserved gene network has been triggered multiple times into action. To date, however, none of these genes has been functionally implicated in eyespot formation. We are currently testing whether several candidate transcription factors and ligands, when ectopically expressed, lead to alterations in eyespot patterns. For this purpose we have developed the technique of germ line transformation for the Nymphalid butterfly *Bicyclus anynana*, and developed a new method for ectopically activating genes on the developing wing in a controlled temporal and spatial fashion. This method makes use of precise laser heat-shocks that activate transgenes via a heat-shock promotor.

# Research article: Role of male sexual pheromones in sexual selection in the African butterfly *Bicyclus anynana*

**DOI:** 10.1673/031.007.2959

**Published:** 2007-05-04

**Authors:** C. Nieberding, V. Schneider, H. De Vos, J.M. Lassance, C. Lofstedt, P.M. Brakefield

**Affiliations:** ^1^ Institute of Evolutionary Biology, Leiden University, The Netherlands; ^2^ Zoogeographical Research Unit, Liege University, Belgium; ^3^ Department of Ecology, Lund University, Sweden. caroline.nieberding@ulg.ac.be

Sexual pheromones are major main trait undergoing natural selection as they ensure, from arthropods to mammals, recognition between conspecific individuals, discrimination between sexes, and mate choice. In particular, female sexual pheromones in insects seem to play key roles in the determination of: *i*) reproductive success at the level of individuals within a species; and *ii*) the reproductive isolation and speciation processes between species (Wyatt TD. 2003. Pheromones and animal behaviour, communication by smell and taste. Cambridge University Press). Whilst interest has focused on female pheromones, male Lepidoptera also produce sexual pheromones, usually during the close-range courtship behaviour. The role of sexual pheromones produced by males in reproductive success and isolation remains largely unknown in insects. In this study, based on the relevant model sub-Saharan African butterfly *Bicyclus anynana* (Butler, 1879) (Nymphalidae), we assessed experimentally the role of male sexual pheromones in mating success and the process of sexual selection by females. Male sexual pheromones in *Bicyclus* are produced by secondary sexual organs, the androconia, present on the wings. In the wild-type stock population of *B. anynana* collected in Malawi and reared in standard conditions in Leiden, we described the courtship behaviour of male *B. anynana* and showed by high speed camera images that these plumes of specialized hairs fan out during courtship and probably distribute pheromones. We identified the three active male compounds secreted by the androconia, i.e. tetradecenol, hexadecanal, and 6,10,14 trimethylpentadecanol using gas chromatography coupled to electroantennograms (GC-EAG) and mass spectrometry (GC-MS). The median composition of the pheromone blend is 19:1:80 of tetradecenol, hexadecanal, and 6,10,14 trimethylpentadecanol respectively, between day 3 and 7 after adult emergence (the peak of male sexual activity). The pheromone blend varies with the age of the males: tetradecenol and trimethylpentadecanol production increases during the first week following adult emergence, then decreases to disappearance at 4 weeks of age. Hexadecanal production starts around day 8 and continuously increases until death. *B. anynana* mating success of mixed populations can be measured in an environment that is ecologically relevant (tropical greenhouse) by tracking mating success via transfer of coloured marker dusts from male to female during copulation (Joron M, PM Brakefield. 2003. Captivity masks inbreeding effects on male mating success in butterflies. Nature 424: 191–194). Competition experiments in this field-like environment showed that the mating success of males is dependent on the presence of androconia. Indeed, males with surgically removed androconia display markedly reduced mating success compared to sham-operated males or stock males. This demonstrates the importance of male sexual pheromones in *B. anynana* mating behaviour. In order to determine whether the composition of their individual pheromone blend may affect male fitness, the mating success of males from the stock population was compared to that of males from the mutant *comet* lineage possessing vestigial androconia and a lower pheromone load. Competition experiments in the tropical greenhouse revealed that *comet* (cc) males display markedly reduced mating success compared to hybrid (c+) or stock (++) males.

# Research article: Nitric oxide is necessary for maintaining *Manduca sexta* antennal lobe neuron activity and odor responsiveness

**DOI:** 10.1673/031.007.2960

**Published:** 2007-05-04

**Authors:** A. Nighorn

**Affiliations:** ARL Division of Neurobiology, University of Arizona Tucson, AZ, USA. nighorn@neurobio.arizona.edu

Nitric oxide (NO) can mediate communication within the nervous system without regard to specific circuitry or synaptic contacts. The unique glomerular architecture of the primary olfactory neuropil along with the high expression of nitric oxide synthase (NOS) in this tissue, has lead to the hypothesis that NO plays an important role in the processing of olfactory information. We are using the moth, *Manduca sexta* as a model to understand the function of NO in the olfactory system. We show that enzymes involved in NO signaling, including NOS and soluble guanylyl cyclase (sGC), are expressed in subsets of neurons within the M. sexta olfactory system and, moreover, that NO is produced in olfactory glomeruli in response to odor stimulation. The function of NO in the olfactory system was examined in individual olfactory neurons with intracellular recording techniques while manipulating levels of NO signaling with pharmacological agents. Blocking NOS with either L-NAME or 7-NI resulted in changes in the behavior of both local interneurons (LNs) and projection neurons (PNs). Both PNs and LNs showed changes in baseline activity, including both increases and decreases in spike firing rate in LNs and the presence of bursts in many PNs. The odor-evoked activity in both neuron types was either missing or altered. The effects were mimicked in several neurons when sGC signaling was blocked using ODQ. However, some of the neurons that were affected by NO blockade did not contain detectable levels of sGC as measured by immunohistochemistry of the recorded and dye-filled neurons. These results indicate that NO has a variety of effects on olfactory neurons and that these effects are mediated by both sGC-dependent and sGC-independent mechanisms. We are currently testing these ideas using RNAi to knockdown the expression of NOS and other genes important for the function and development of the olfactory system. We will discuss our current progress in these attempts to analyze olfactory function and development using RNAi. This work is funded by NIH -NIDCD DC04292

# Research article: The wide utility of lepidopteran genomic resources

**DOI:** 10.1673/031.007.2961

**Published:** 2007-05-04

**Authors:** A. Papanicolaou, M.L. Blaxter, C.D. Jiggins

**Affiliations:** ^1^ Max Planck Institute for Chemical Ecology, Jena, Germany; ^2^ Institute of Evolutionary Biology, University of Edinburgh, Edinburgh, UK. apapanicolaou@ice.mpg.de

Lepidopteran research would greatly benefit from the accumulation of genomic data. For this reason, a number of labs are investing in the development of resources such as sequences from large and small insert genomic or cDNA libraries. Such information-rich data needs to be analysed in an efficient manner. Transcriptomic analyses in Lepidoptera have been hindered by the fact that, until recently, no public datasets were available other than the model system of *Bombyx mori*, thereby preventing comparative studies within the Lepidoptera. Here I present a powerful, user-friendly Internet database for the few Lepidoptera cDNA projects currently deposited in the public domain, including those from *Bombyx mori, Bicyclus anynana, Heliconius sp*., *Manduca sexta* and *Spodoptera frugiperda*. The aim of ButterflyBase (available from http://www.heliconius.org) is to create highly annotated, non-redundant gene objects and protein predictions and to provide the means to extend the utility of EST datasets beyond the original intent of the sequencing lab. I outline how ButterflyBase can be used as a gene finding and comparative tool by designing specific molecular markers for phylogenetic and linkage mapping studies, identifying Lepidoptera specific, homologous or candidate genes and annotating cDNA AFLPs markers. Although it is evident that sequencing projects are of high value to the wider Lepidoptera community and public datasets need to both increase and diversify, the community also needs to support these efforts through the development of a suite of integrated bioinformatic applications.

# Research article: Modular promoter architecture and transcriptional regulation of silkmoth chorion genes

**DOI:** 10.1673/031.007.2962

**Published:** 2007-05-04

**Authors:** A. Papantonis, S. Sourmeli, R. Lecanidou

**Affiliations:** Dept. of Biochemistry and Mol. Biology, Faculty of Biology, University of Athens, Greece, apapant@biol.uoa.gr

The epithelial cells that surround the developing silkmoth oocyte comprise the sole locus where chorion genes can be found in a transcriptionally active state. The process of choriogenesis is governed by alternating phenomena of gene activation and repression. This intricate course of events is thought to require the recruitment of multiple transcription factors that target gene promoter modules with temporal-specificity, thus leading to their up- or down-regulation.

In this study we provide *in vitro* as well as *in vivo* evidence for the direct implication of BmC/EBP in chorion gene regulation. Primarily, the expression profile of BmC/EBP on the transcriptional level is closely correlated with the activation of early and early-middle chorion genes. Moreover, its translational profile is restricted to choriogenic stages and quantitatively matches its transcription pattern. We performed gel retardation assays where recombinant BmC/EBP was allowed to interact with early or late-specific promoter fragments. The formed complexes denoted strong interaction with characterized *cis*-elements. They also exhibited the same migration and intensity as staged follicular nuclear extracts. The key role of BmC/EBP was finally revealed when short antisense DNA fragments designed to repress its function were introduced into isolated ovarioles *ex vivo*. When the growing ovarioles were harvested and RT-PCR was performed, we witnessed a repression of chorion gene expression relative to the down-regulation of BmC/EBP. Additional mobility shift assays followed our observation of the significant dispersion of C/EBP elements in chorion gene promoters from close (*Anthaerea* sp.) or distant insect relatives (*C. capitata, D. melanogaster*) of *B. mori*. Previous data from in *vivo* trials, using the respective promoter fragments, identified DNA stretches which included one or more of these sequences as repressor and/or activator elements. This combination of *in vitro* and *in vivo* data allowed us to evaluate the impact of modular chorion gene promoter architecture on the development of DNA-protein interaction in a specific and orderly manner. The above are discussed as a whole and lead to the presentation of a parsimonious model for role of BmC/EBP in insect choriogenesis. This study also includes our first attempt to implicate proteins bearing both an ‘architectural’ and a ‘regulatory’ role in the chorion gene regulation cascade. We investigated the role of BmHMGA and BmCHD1, two proteins orthologous to mammalian High Mobility Group and Chromo-Helicase/ATPase-DNA binding proteins respectively. BmHMGA is mainly transcribed during late vitellogenesis, whereas BmCHD1 is expressed in various choriogenic stages, while exhibiting higher protein availability during middle choriogenesis. Both molecules were expressed as hexahistidine fusions, affinity purified and used in gel retardation assays. We observed distinct binding to chorion gene promoter fragments regardless of their developmental descent, which was characterized by high specificity and affinity. The interaction grew stronger when both proteins bound the respective fragments synergistically, with each other or with BmC/EBP. Gel retardation assays with DNA fragments derived from the pBend2 vector allowed us to evaluate the effect of BmHMGA on the curvature of full length promoters. Furthermore, the exact binding positions were footprinted using Dnase I and the topology of the respective *cis*-elements is discussed by focusing on middle chorion gene promoter architecture.

This work is in part supported by a GSRT grant ‘PENED 2003’ program code no: 03ED 124)

# Research article: Identification and characterization of olfactory genes in the antennae of the silkmoth: *Bombyx mori*

**DOI:** 10.1673/031.007.2963

**Published:** 2007-05-04

**Authors:** J. Pelletier, I. Brigaud, S. Malpel, M. Da Rocha, M. Maïbèche, C. Royer, E. Jacquin-Joly

**Affiliations:** ^1^ UMR PISC, «Physiologie de l'Insecte: Signalisation et Communication », INRA centre de Versailles, Route de Saint-Cyr, 78026 Versailles cedex, France.; ^2^ UNS INRA, 25 quai Jean-Jacques Rousseau, 69350 La Mulatière, France. Julien.Pelletier@versailles.inra.fr

Olfaction is widely used by insects to communicate, well exemplified by the sex pheromonal communication system of moths by which females emit a pheromone to attract conspecific males for mating. This system presents a remarkable sensitivity and specificity that have been deeply studied in the silkworm moth *Bombyx mori*. Recently, genomic resources were developed on this species and are publicly available, including numerous EST libraries and genome sequencing. Biotechnological tools such as transgenesis are also available, then offering the possibility to decipher the molecular basis of olfaction in this species. The pheromone of *B. mori* was the first one identified and consists of bombykol (Bol), a C-16 alcohol, in association with its aldehyde oxidized form, the bombykal (Bal), whose precise function still remains unclear. These molecules encounter different steps in the antennae. They are first supposed to bind pheromone binding proteins (PBPs) in order to cross the aqueous lymph of the sensilla that housed the olfactory neurons (ONs). Then, they interact with olfactory receptors anchored in the dendritic membrane of the neurons. The male pheromone sensilla houses two kinds of ONs, one responsive to Bol, the other to Bal, and two pheromone receptors have been to date identified, each expressed in one of these neurons. Up to now, only one PBP (BmPBP1) has been identified in *B. mori* antennae and it has been shown to bind Bol. However, some studies in different moth species revealed that several PBPs are usually expressed in the antennae. Together with functional data showing specific binding of PBPs with pheromonal components, this diversity within a species lets suggest that PBPs may participate in the first step of odor discrimination. The presence of other PBP(s) in *B. mori* antennae is then strongly suggested. Taking advantages of the available genome, we identified by bioinformatic approaches two new PBP genes (BmPBP2 and BmPBP3). They are clustered with BmPBP1 on a single chromosome location and they present 40–50% amino-acid identity between them. Their deduced amino acid sequences revealed common features of PBPs, in particular a secretion signal peptide and 6 cysteins in conserved positions. RT-PCR and Northern Blot experiments were performed and showed an antennal expression of both genes. Interestingly, BmPBP3 seemed more expressed in female than in male antennae, whereas BmPBP2 seemed equally expressed in antennae of both sexes. In addition, several new putative olfactory receptors were discovered by BLAST analyses using already known insect olfactory receptors (*Drosophila, Anopheles, Heliothis* uirescens), that presented typical G-protein coupled receptor features. Two transmembrane domain receptors were also identified that showed homologies with insect Sensory Neuron Membrane Proteins (SNMPs), a lepidopteran family of ON-specific receptors of unknown function. Functional tools are now developed to address the respective contribution of the newly identified PBPs and receptors to the peripheral olfactory processes.

# Research article: *Toxoneuron nigriceps* bracovirus (*Tn*BV) and its role in the host regulation

**DOI:** 10.1673/031.007.2964

**Published:** 2007-05-04

**Authors:** F. Pennacchio, P. Falabella, P. Varricchio, C. Malva

**Affiliations:** ^1^ Dipartimento di Entomologia e Zoologia Agraria “F. Silvestri”, Università; di Napoli “Federico II”, Portici (NA), Italy; ^2^ Dipartimento di Biologia, Difesa e Biotecnologie Agro-Forestali, Università della Basilicata, Potenza, Italy; ^3^ Istituto di Genetica e Biofisica, CNR, Napoli, Italy. pennacchio@unibas.it

Polydnaviruses (PDVs) are obligate symbionts of Hymenoptera parasitoids of lepidopteran larvae, which induce host immunosuppression and physiological redirection. PDVs include bracoviruses (BVs) and ichnoviruses (IVs), which are associated with braconid and ichneumonid wasps, respectively. *Toxoneuron nigriceps* is an endophagous parasitoid of the tobacco budworm, *Heliothis virescens*. Parasitized host larvae show the suppression of the immune response and are unable to attain the pupal stage, becoming developmentally arrested as mature larvae. This complex syndrome is largely induced by a symbiotic bracovirus (*Tn*BV), which is injected by the adult wasps at the oviposition. *Tn*BV genome has been sequenced and several genes expressed in parasitized hosts have been cloned and characterized. A total of 20 circular DNA segments, ranging from 3.5 to 13 kb, have been fully sequenced. Like observed in other PDVs, the overall sequence has a strong bias toward A-T content (69%), and is largely non-coding. Each circle contains at least 1 coding gene and no more than 4, which are often interrupted by introns and are members of gene families. Some of these gene families have been found in different BVs and IVs, while others appear to be unique. The current study of *Tn*BV genes expressed in parasitized hosts provides new insights on the possible regulation mechanisms of host immune disruption and developmental arrest induced by *T. nigriceps* parasitism. The functional analysis of some of these genes indicates that they are involved in immune suppression, by inducing apoptosis of haemocytes or by disrupting NF-χB signalling pathways. This latter alteration is determined by IχB-like (ANK) proteins, similar to those of insects and mammals, but characterized by shorter ankyrin domains and by the absence of regulatory domains. The phylogenetic analysis of PDV ANK proteins indicates that they have a common origin, even though BVs and IVs are thought to be unrelated. The evolutionary implications of this finding are discussed.

# Research article: Elevated rates of opsin amino acid evolution following gene duplication in *Lycaena* butterflies (Lepidoptera)

**DOI:** 10.1673/031.007.2965

**Published:** 2007-05-04

**Authors:** Nelida Pohl, Marilou Sison-Mangus, Adriana D. Briscoe

**Affiliations:** Comparative and Evolutionary Physiology Group, Department of Ecology and Evolutionary Biology, University of California, Irvine, CA, 92697, U.S.A. npohl@uci.edu

The process by which genes acquire new functions is not well understood, but gene duplication is considered an important mechanism for generating functional diversity. Gene duplications are often followed by an accelerated rate of evolution. We tested this hypothesis by examining the evolution of a pair of blue opsin duplicate genes in the butterfly genus *Lycaena*. Visual pigments are the light-sensitive molecules in the arthropods' compound eye. The specific amino acid sequence of the opsin protein determines the peak absorption maximum of the visual pigment. We used PCR, cloning and sequencing of eye derived cDNAs to characterize all four opsin genes of *L. heteronea* and *L. helloides*, which we combined with the previously characterized *L. rubidus* opsin sequences. The translated opsin amino acid sequences were aligned and used to construct a phylogenetic tree. Each of these sequences falls within one of three well-supported clades in the insect opsin gene tree, comprised respectively of ultraviolet (UV), blue (B) and long-wavelength (LW) sensitive pigments. One of these genes belongs to the ultraviolet (UV) opsin clade, and encodes the visual pigment with peak sensitivity to 360 nm. A second gene clusters within the long wavelength (LW) clade and corresponds to the visual pigment with peak sensitivity to 568 nm. Two genes clustered within the blue-sensitive opsin clade (B1 and B2), representing respectively the visual pigments with peak sensitivity to 437 (blue) and 500 (green) nm. We used Tajima's (1993) method to test whether or not the P500 opsin displayed an elevated rate of amino acid evolution following its divergence from the P437 opsin, which has a more typical peak sensitivity for opsins of that clade. Visual inspection of the branch lengths of the opsin tree suggested that Bi and B2 are evolving at different rates, and results from Tajima's test support this finding for all three *Lycaena* species (p<0.05 for all species). A second analysis separating the transmembrane (TM) and the non-transmembrane (Non-TM) domains of the opsin protein, shows that only the amino acid differences between the TM domains are responsible for this different rate of evolution between Bi and B2 in all three species (TM domains p<0.01, Non-TM domains p>0.4), further suggesting that one of these genes has evolved a new function.

# Research article: Embryonic development and wing colour patterns in the tropical butterfly, *Bicyclus anynana*

**DOI:** 10.1673/031.007.2966

**Published:** 2007-05-04

**Authors:** S.V. Saenko, P.M. Brakefield, P. Beldade

**Affiliations:** Evolutionary Biology, Institute of Biology, Leiden University, Kaiserstraat 63, 2311 GP Leiden, The Netherlands, s.v.saenko.2@umail.leidenuniv.nl

Butterfly wing patterns provide an attractive system for studying interactions between the evolutionary and developmental processes that shape morphological variation. The African butterfly *Bicyclus anynana* with its conspicuous eyespots has been established as an “evo-devo” laboratory model and successfully used to study the genetic mechanisms underlying variation in wing patterns. The problem, however, is that none of the insect model species has eyespot patterns which appear to be a Lepidoptera-specific trait. Genetic comparison and identification of the genes involved in colour pattern formation become a challenge when genomic resources are only starting to be developed. Over twenty mutant stocks with dramatically altered eyespot pattern are maintained in our laboratory and several of them appear to have disturbed embryonic development. Examination of the genes with such pleiotropic effects and the search for comparable mutations in *Drosophila melanogaster* and *Bombyx mori* can help unravel the signaling pathways involved in embryonic and wing pattern development. We studied embryogenesis in “wild-type” butterflies and in mutants with altered eyespot size and colour composition. Homozygotes for these mutant alleles die during the first days of development; heterozygotes develop normally but show the disturbed eyespot pattern. Expression of several developmental genes such as *Engrailed* was examined in “wild-type” and mutant embryos and showed similarities with some of the Drosophila segment polarity mutants. This suggests candidate developmental pathways potentially involved in the phenotypic variation for eyespot pattern. We will discuss the significance of the research and future perspectives.

# Research article: CCCH-type zinc finger genes: Candidate regulators of sex determination pathway in the silkworm, *Bombyx mori*

**DOI:** 10.1673/031.007.2967

**Published:** 2007-05-04

**Authors:** V. Satish, J.N. Shukla, J. Nagaraju

**Affiliations:** Laboratory of Molecular Genetics, Centre for DNA Fingerprinting and Diagnostics, Hyderabad-500076, INDIA. jnagaraju@cdfd.org.in

Doublesex gene in the silkworm, *Bombyx mori*, conforming to the bottom-up hypothesis, splices into male and female isoforms, which are responsible for the subsequent male or female differentiation respectively. But the regulators of the splice state of doublesex have been elusive, except for the identification of a female-determining region on the W chromosome. Differential display analysis on a strain possessing a W-harboured translocation, resulted in the identification of a W-linked C-x8-C-x5-C-x3-H zinc-finger domain-containing gene. RT-PCR analysis showed that the zinc finger gene is expressed very early in the development prior to the expression of dsx gene and also through out the life cycle and in all the tissues. We also identified two other putative genes containing the C-x8-C-xs-C-x3-H domain present on the autosome. Sequence analysis suggested an initial duplication, followed by a translocation on to the W-chromosome and subsequent sequence divergence. The W-linked gene is found to be *Bombyx*-specific as identified by PCR performed on different lepidopteran species, while the autosomal genes are not. Transient RNAi assays revealed a possible interaction between the zinc-finger genes in determining the splice state of the downstream gene, doublesex, in a direct or indirect manner. Thus we propose a model for the sex determination mechanism in the silkworm involving the zinc-finger genes.

# Research article: Hemocytes of *Manduca sexta*: new data about hemocyte components, subpopulations, and their phagocytic capability

**DOI:** 10.1673/031.007.2968

**Published:** 2007-05-04

**Authors:** Scholz Frank, Lesch Tine, Beez Susann, Holthusen Traute, Anderl Ines, Silvia Geuenich, Trenczek Tina

**Affiliations:** Institute of General and Applied Zoology, Justus-Liebig University of Giessen, Stephanstreet 24, D-35390 Giessen, Germany. Tina.E.Trenczek@allzool.bio.uni-giessen.de

The focus of our research interest is the analysis of the function of hemocytes and their differentiation to become immune competent cells, in particular of the model insect *Manduca sexta*. Within recent years we have collected data on their numbers, distribution, appearance, subpopulations and fluctuations during wounding, infection and postembryonal development as well as embryonal development (see also accompanying poster Gökçen et al.) using classical and modern histological, immunohistochemical and biochemical methods. In addition we have developed a cDNA library of hemocytes and of fat body of infected larvae. A few cDNA clones of proteins that may play an important role in wound and immune reactions have been characterized in more detail. The presentation will provide an overview of the present state of knowledge available from our research group. This includes sequence data of some hemocyte cDNA clones and some characteristics of their corresponding proteins as well as new aspects of hemocyte characteristics obtained by in vivo and in vitro experiments. Research has been performed in part in stimulating collaboration with the research group of Prof. M.R. Kanost (KSU Manhattan, USA) and was supported by several DFG grants (Tr254/xx).

# Research article: Development of enhancer trap mutagenesis in *Bombyx mori*

**DOI:** 10.1673/031.007.2969

**Published:** 2007-05-04

**Authors:** H. Sezutsu, K. Uchino, K. Kojima, I. Kobayashi, T. Niimi, M. Hatakeyama, T. Tamura

**Affiliations:** ^1^ Transgenic Silkworm Research Center, National Institute of Agrobiological Sciences, Tsukuba, 305–8634 Japan; ^2^ Silk-Materials Research Unit, National Institute of Agrobiological Sciences, Tsukuba, 305–8634 Japan; ^3^ Grad. School of Bioagricultural Sciences, Nagoya University, Nagoya 464–8601, Japan; ^4^ Invertebrate Gene Function Research Unit, National Institute of Agrobiological Sciences, Tsukuba 305–8634, Japan. hsezutsu@affrc.go.jp

We are developing tools for functional genomics in *Bombyx mori*. The genome draft sequence of *B. mori* was released in 2004. In the post-genomic study, binary expression system, enhancer trap, gene trap, insertional mutagenesis, gene targeting and RNAi will be important tools for functional analysis of the genome. We have been developing enhancer trap system in silkworm to control transgene expression and to identify novel gene function. At first, GAL4/UAS binary system for targeted gene expression were adopted with EGFP as the reporter using *piggyBac* transposon-derived vector. Jumpstarter strain, which provides *piggyBac* transposase and promotes remobilization of mutator (promoter-GAL4) transposon, was also established using *Minos* transposon. We have analyzed the efficiency of transposition of mutator and its position effects. When mutator transposon moves and traps genomic enhancer/silencer, the reporter (UAS-EGFP) will show stage- and tissue-specific expression. In the feasibility study, cytoplasmic actin A3 promoter of *B. mori* was used for mutator (A3-GAL4) to test whether or not the mutator can detect enhancer/silencer activity. In the A3-GAL4 lines, the expression patterns of EGFP varied greatly due to the insertion positions of the mutator. We have obtained the strains in which GAL4 expressed at various organs. Then, we have developed tTA/TRE binary system. In the system, transcription of transgene will be turned off in the presence of tetracycline or doxycycline (Tet-OFF). We have produced transgenic silkworm with hsp-tTA transactivator and TRE-EGFP response element that were used in *Drosophila melanogaster*. The heat shock promoter (hsp) of *Drosophila* was weakly activated by heat shock treatment in *B. mori*. One of the four transgenic strains showed ubiquitous expression of EGFP without heat shock, possibly by enhancer trapping. The expression of EGFP was decreased when tetracycline/doxycycline was added to the artificial diet. In addition, the effect of tetracycline was transmitted to eggs maternally. The results showed that tTA/TRE system works in *B. mori*. GAL4/UAS and tTA/TRE binary systems should be useful for enhancer trap mutagenesis and control of transgene expression in the silkworm.

# Research article: Developmental characteristics of transgenic silkworm with overexpression of juvenile hormone esterase

**DOI:** 10.1673/031.007.2970

**Published:** 2007-05-04

**Authors:** Takahiro Shiotsuki, An-Jiang Tan, Toshiki Tamura

**Affiliations:** ^1^ Invertebrate Gene Function Research Unit, National Institute of Agrobiological Sciences; ^2^ Transgenic Silkworm Research Center, National Institute of Agrobiological Sciences, Tsukuba 305–8634, Japan. shiotsuk@affrc.go.jp

In insect physiology, especially in development and metamorphosis, juvenile hormones (JHs) have important roles but studies of the molecular mechanisms of the JH have not been progressed much yet. Hydrolysis of the methyl ester of JH by a JH-specific esterase is a key pathway for the degradation of JH to regulate JH titer in the hemolymph. We generate transgenic silkworm strains that overexpress the JH esterase using the GAL4/UAS system. First, we confirmed high expression of JH esterase mRNA from embryo to 3rd stadium. Enzymatic activity of the JH esterase in the hemolymph of 2nd and 3rd instar of the transgenic line is 8 to 10 times higher than that of middle stage of the 5th instar, which is the highest activity through the whole stage of wild-type. It was also found that the high JH esterase activity in whole body homogenates at embryo and 1st stadium in the transgenic line. The overexpression of JH esterase from the embryonic stage resulted in larval-pupal metamorphosis after the third stadium, two stadia earlier than that observed in wild-type insects. This precocious metamorphosis occurred after 3rd instar indicates that JHs are not critical for normal development of embryo or larva before the second molt in Lepidoptera. The transgenic approach developed in this study allowed us to dissect the function of key physiological events that occur from embryogenesis. These types of studies were only possible in later larval stadia using physical techniques such as allatectomy or the application of JH analogs. The experimental system with the overexpression of JH esterase will allow further studies in insect endocrinology and physiology related to JH action.

# Research article: An EST library from the midgut of the Lightbrown Apple Moth, *Epiphyas postivittana* (Walker) (Lepidoptera: Tortricidae)

**DOI:** 10.1673/031.007.2971

**Published:** 2007-05-04

**Authors:** Robert Simpson, Richard Newcomb, Lesley Beuning, Yah-Khing Yauk, Ross Crowhurst, Heather Gatehouse, Laurence Gatehouse, Ngaire Markwick, Dave Chagne, Andrew Gleave, John Christeller

**Affiliations:** ^1^ HortResearch, Private Bag 11030, Palmerston North, New Zealand; ^2^ HortResearch, Private Bag 92169, Auckland, New Zealand. rsimpson@hortresearch.co.nz

The lightbrown apple moth is a quarantine pest of New Zealand fruit, and is also closely related to a complex of native leafroller pests which cause economic damage. The larval midgut is the target of many control strategies, and an understanding of the genomics of the midgut has the potential to unearth new targets for pest control. We have produced an EST library that will allow identification of genes of the midgut involved in digestion, growth and pathogen interactions, as well as revealing active genes of unknown function. Sequence analysis of midgut cDNA libraries now covers ∼ 6,000 good quality ESTs from 3^rd^, 4^th^ and 5^th^ instar larva midguts. Clustering yielded a unigene set of ∼ 1,200 contigs. Searches against the SwissProt database and model organism proteomes yielded confident annotations for ∼ 60% of the contigs, with approximately 20% of these with unknown function. The remainder were involved in digestion, nutrient transport, energy transfer, peritrophic membrane synthesis and cell maintenance and proliferation. Some groups of genes, including serine proteases and aminopeptidases have been analysed. The library has been used in conjunction with proteomic data from the brush border membrane to discover the depth of EST coverage and to identify potential pest control targets.

# Research article: Polydnavirus-mediated suppression of the insect immune response

**DOI:** 10.1673/031.007.2972

**Published:** 2007-05-04

**Authors:** M. R. Strand

**Affiliations:** Department of Entomology, University of Georgia, Athens GA 30602, USA. mrstrand@bugs.ent.uga.edu

Insects rely upon a well-coordinated innate immune system for protection against invading pathogens and parasites. Larger, multicellular parasites are usually killed by encapsulation, which involves attachment of multiple layers of hemocytes to the foreign target. Smaller pathogens, in contrast, are killed by a combination of hemocyte-mediated phagocytosis and humoral defenses. Despite the fundamental importance of these responses, our understanding of their regulation in relation to the counter strategies pathogens use to evade host defense responses is limited. Viruses in the family Polydnaviridae are symbiotically associated with parasitoid wasps and are among the most virulent immunosuppressive pathogens of insects. Polydnaviruses are divided into two genera, bracoviruses (BVs) and ichnoviruses (IVs), on the basis of their association with wasps in the families Braconidae and Ichneumonidae. Genome analysis reveals important similarities in the organization BV and IV genomes but these viruses share almost no sequence homology with one another suggesting their association with parasitoids arose independently. Functional analysis has also identified several key genes involved in immunosuppression. Notably, most virulence factors encoded by polydnaviruses like *Microplitis demolitor* bracovirus (MdBV) target signaling pathways that regulate important immune effector responses rather than effector molecules themselves. Overall, these results provide important insight on the evolution of polydnaviruses and also identify key virulence determinants underlying immunosuppression.

# Research article: Insect cell-based high-throughput screening systems for the identification of compounds with ecdysteroid mimetic insecticide activities

**DOI:** 10.1673/031.007.2973

**Published:** 2007-05-04

**Authors:** L. Swevers, D. Stefanou, T. Soin, K. Van Loocke, G. Smagghe, C. Wheelock, T. Harada, M. Akamatsu, Y. Nakagawa, K. Iatrou

**Affiliations:** ^1^ Insect Molecular Genetics and Biotechnology, Institute of Biology, NCSR “Demokritos”, Aghia Paraskevi, Athens, Greece; ^2^ Laboratory of Agrozoology, Faculty of Agricultural and Applied Biological Sciences, University of Ghent, Ghent, Belgium; ^3^ Division of Applied Life Sciences, Graduate School of Agriculture, Kyoto University, Kyoto, Japan. swevers@bio.demokritos.gr

Insect pests are responsible for global crop losses of 20–50% of potential production and for transmitting some of the world's most important diseases such as malaria, filariasis and dengue fever. The majority of methods to control insect pest populations involve the use of chemical compounds that are toxic to the environment and human health. A great need exists for the development of alternative methods of insect pest control that are characterized by higher specificity, lower toxicity and increased potency. Compounds that specifically disrupt the hormonal balance in insects have great potential to be developed as alternative, specific and environmentally friendly insecticides. Important hormonal targets in insects include the molting hormone, 20-hydroxy-ecdysone (20E), and the “anti-metamorphosis” hormone, juvenile hormone (JH). However, the search and identification of compounds that disrupt the insect endocrine system has been hampered by the lack of availability of high-throughput screening systems. To address this need, we have developed transformed silkmoth (Bombyx mori)-derived Bm5 cell lines that respond to the addition of ecdysone-like substances through the expression of the green fluorescent protein (GFP) and the appearance of green fluorescence. Because the amount of green fluorescence can be easily quantified in individual wells of a 96 well plate by a fluorescence plate reader, the engineered cell lines can be used for the screening of compounds with ecdysteroid mimetic activity in high-throughput format. The generated reporter cell lines are used for the screening of plant extracts from available plant collections and for monitoring subsequent activity during enrichment and purification steps. The cell lines are also used to screen chemical libraries of dibenzoyl hydrazine compounds to assess structure-activity relationships. Measurements of EC50 values of a large collection of compounds were used to construct a quantitative structure-activity relationship (QSAR) model that describes the ecdysone agonist activities of the dibenzoyl hydrazine analogs. Finally, for some of the identified agonists, it was shown that their activity as determined by the cell-based screening assays parallels their bioactivity in growth inhibition and toxicity assays using live insects. While the current high-throughput screening system targets lepidopteran insects (moths and butterflies), future work aims at the expansion of the technology to cell lines that originate from other insect orders, such as Diptera (flies and mosquitoes) and Coleoptera (beetles). The availability of such insect order-specific screening systems is predicted to assist significantly in the search of new compounds that target the insect endocrine system with increased specificity and potency. This work is supported by a grant of the General Secretariat of Science and Technology, Greek Ministry of Development (Bilateral S & T Cooperation Greece-Japan, 05NON-EU-78).

# Research article: A direct role of JH in the control of imaginai disc formation and growth in *Manduca*

**DOI:** 10.1673/031.007.2974

**Published:** 2007-05-04

**Authors:** JW Truman, K Hiruma, JP Allee, SGB MacWhinnie, D Champlin, LM Riddiford

**Affiliations:** ^1^ Department of Biology, University of Washington, Seattle, WA 98195–1800, USA; ^2^ Faculty of Agriculture and Life Sciences, Hirosaki University, Hirosaki 036–8561, Japan; ^3^ Department of Biological Sciences, University of Southern Maine, Portland, ME 04103, USA. lmr@u.Washington.edu

In Lepidoptera the eye and the leg imaginai discs form only in the final larval instar from imaginal primordia that make larval cuticle during the earlier instars but remain diploid. Formation of these discs in the tobacco hornworm, *Manduca sexta*, begins about 18 hr after ecdysis with the appearance of Broad in these cells and the detachment of the primordium, followed by the onset of proliferation by 24 hr. Starvation from the time of ecdysis prevents this formation, which can be restored by feeding on sucrose plus casein; sucrose only permits the up-regulation of Broad, but not proliferation. By contrast, these discs form and grow slowly in starved allatectomized larvae lacking juvenile hormone (JH), and this formation can be prevented by JH. Ligation experiments show that this disc morphogenesis induced by the removal of JH is independent of ecdysteroid action. Manipulation of starvation and JH treatments both *in vivo* and *in vitro* showed that JH acted directly on the primordia to suppress morphogenesis, but that a second unidentified factor dependent on nutrients is necessary for the morphogenesis to occur. This factor that we call “metamorphosis initiating factor” appears only in the final instar and can override the JH suppression of disc formation. Thus, disc growth in the final instar is comprised of both morphogenetic growth under the suppressive control of JH and nutrient-dependent growth. One major role of JH then during larval life is to allow isomorphic growth of these imaginai primordia as the larva grows. This suppression of morphogenesis is also seen in embryos of more basal insects where premature exposure to JH suppresses embryonic patterning and induces precocious terminal differentiation. Thus, the ancient role of JH is to allow switching between growth and morphogenesis. Supported by grants from NSF to JWT and to LMR, USDA to LMR, Japan Society for the Promotion of Science to KH, and Bioscience Research Institute of Southern Maine to DTC.

# Research article: Interactions of polydnavirus-encoded gap junctions with lepidopteran cells

**DOI:** 10.1673/031.007.2975

**Published:** 2007-05-04

**Authors:** M.W. Turnbull

**Affiliations:** Department of Entomology, Soils, and Plant Sciences, Clemson University, Clemson, S.C. 29634–0315 U.S.A. turnbul@clemson.edu

Gap junctions occur in all multicellular animals, and provide direct cell-cell transfer of small molecules between adjacent cells. Through this selective communication, the structures play important roles in coordination of multicellular behaviors; consequently, disruption of gap junctional communication is highly correlated to a variety of pathological states. Although gap junctions have been long recognized to form between a variety of cell types in insects, the failure to isolate the encoding genes and proteins comprising the structures prohibited in-depth dissection of their roles. Conversely, analysis in mammals proceeded rapidly, isolating the connexin genes and proteins that compose gap junctions in those organisms. It is now known that gap junctions in insects are formed by a family of proteins that are non-homologous to the chordate connexins, and these evolutionarily distinct, yet functionally analogous, proteins have been termed innexins. Like connexins, innexins polymerize to form hemichannels, and gap junctions are formed by the interaction of hemichannels of adjacent cells. Recent work has demonstrated that the characteristics of a given gap junction are determined by the specific members of the innexin gene family which comprise the structure. Indeed, as with connexins in mammals, experimental alteration of gap junction composition has strongly negative consequences in insects. Sequencing of the genome of *Campoletis sonorensis* Ichnovirus (CsIV) (Polydnaviridae) resulted in the identification of four *innexin* homologues, termed *vinnexins*. All four genes are transcribed in *Cs*IV-infected *Heliothis virescens*, and antisera raised against the Cs-VnxQ2 protein localizes uniquely to hemocytes (Turnbull et al. 2005, *Curr Biol* 14, R491-3). Furthermore, two of the vinnexins, Cs-VnxD and Cs-VnxG, form electrophysiologically functional gap junctions in *Xenopus laevis* oocytes. Sequencing efforts of other labs have demonstrated that the *vinnexins* are both widely conserved and highly diversified across other ichnoviruses, suggesting that the proteins are likely involved in IV-associated pathologies in infected caterpillars. We hypothesize that the vinnexins act to alter gap junctional intercellular communication between vinnexin-expressing cells and other cells of the lepidopteran, disrupting host physiology. As at least one vinnexin localizes primarily to hemocytes, it seems most likely that the role of the vinnexins in *Cs*IV-mediated host physiological disruption is to disrupt immunity. Current work in the lab therefore is focused on understanding the role of native innexins in cellular immune responses of lepidopteran larvae, and on the functional biology of the vinnexins. We will detail the current state of our knowledge concerning the lepidopteran *innexins*, particularly those that are active in the hemocytes of larvae and in cell culture. We have, to date, isolated from *Pseudoplusia indudens* two novel *innexins* which are orthologues of *Drosophila inx1* (*ogre*) and *inx2*, and found both to be transcribed widely throughout juvenile development. We also have developed epitope tagged vinnexin fusion proteins and demonstrated that they exhibit similar subcellular localization as do *Drosophila* innexins and Cs-VnxQ2. We have now begun using the isolated gene products, in combination with standard and novel molecular, biochemical, and physiological approaches, to ask questions concerning the biologies of both innexin- and vinnexin-composed gap junctions. This multifaceted approach should aid in clarifying the role of gap junctions in lepidopteran biology, including their potential for manipulation by polydnaviruses.

# Research article: Moth W chromosomes under magnifying lens of CGH and ZOO-FISH

**DOI:** 10.1673/031.007.2976

**Published:** 2007-05-04

**Authors:** M. Vitkova, S. Kubickova, F. Marec

**Affiliations:** ^1^ Faculty of Biological Sciences, University of South Bohemia, CZ-370 05 Ceske Budejovice, Czech Republic,; ^2^ Biological Centre, ASCR, Institute of Entomology, CZ-370 05 Ceske Budejovice, Czech Republic; ^3^ Veterinary Research Institute, CZ-62132 Brno, Czech Republic. vitkova@entu.cas.cz

It is generally believed that the sex chromosomes in XY or WZ systems evolved from a pair of autosomes. Evolutionary processes such as restriction of recombination between X and Y or Z and W, followed by gradual decay of genes and accumulation of repetitive sequences and transposones in Y or W, have been forming the Y or W chromosomes until they become (almost) genetically inert. This can end in the loss of Y or W, and the life cycle of sex chromosomes starts again. The full evolutionary life cycle of sex chromosomes can be found also in Lepidoptera, the insects with female heterogamety and a WZ/ZZ system (female/male) or derived variants. Recently, we started to study molecular evolution of the lepidopteran sex chromosomes. Here we tried to uncover, whether the W chromosomes are composed more of female specific DNA or simply accumulate junk DNA sequences from autosomes, and how similar are the W chromosomes of different species to each other. Our experimental objects were four representatives of the lepidopteran family Pyralidae, the flour moth, *Ephestia kuehniella*, the almond moth, *Cadra cautella*, the Indian meal moth, *Plodia interpunctella*, and the wax moth, *Galleria mellonella*. These species has an old W-Z pair of sex chromosomes, which are highly differentiated from each other. First we tested similarity of their W chromosomes. For this purpose, we prepared a W chromosome-specific probe derived from laser-microdissected W chromatin bodies of *Ephestia* females. The probe was cross-hybridized to chromosome spreads of the other three species with the aim to detect common parts of their W chromosomes. Although this approach (the so-called ZOO-FISH) is widely used for the detection of homeologous chromosomes in fairly distant species of vertebrates, in the pyralid moths it revealed a surprisingly low similarity between their W chromosomes. While in *Ephestia* the probe labelled the W chromosome equally, hybridization signals were scattered in *Cadra* and *Plodia*, both belonging to the same subfamily Phycitinae, and almost absent in *Galleria*, a member of different subfamily Galeriinae. The results suggest rapid molecular evolution of the non-recombining W chromosomes, resulting in great dissimilarities between W chromosomes of even closely related species. In next experiments, we examined gross molecular composition of the W chromosomes by means of comparative genomic hybridization (CGH). In each species, genomic DNAs from males and females were labelled by a different fluorochrome and hybridized to female chromosome spreads in the presence of an excess of unlabelled male genomic DNA. Results of CGH suggest that the W chromosomes of *Ephestia* and *Cadra* are composed mainly of repetitive DNA common for both sexes but accumulated in the W chromosomes, whereas *Plodia* and *Galleria* possess large regions of female specific DNA sequences in their W chromosomes.

# Research article: Can insects manipulate plant defenses?

**DOI:** 10.1673/031.007.2977

**Published:** 2007-05-04

**Authors:** H. Vogel, J. Kroymann, A. Mithöfer, W. Boland, D. Heckel

**Affiliations:** Max Planck Institute for Chemical Ecology, Hans-Knöll-Strasse 8, 07745 Jena, Germany, Dpts. of Entomology^1^,; Genetics & Evolution^2^,; Bioorganic Chemistry^3^. hvogel@ice.mpg.de

Coevolution between plants and their insect herbivores played a significant role in the “origination of organic diversity”. In the long course of plant-insect interaction, plants have developed sophisticated mechanisms to ward off insect attack. In addition to preformed chemical and physical barriers, plants have evolved induced protection mechanisms, involving biosynthetic processes and/or the up-regulation of gene expression both locally and systemically by attacking herbivores. We are studying these highly complex, yet very specific plant defenses, their inducibility against insects such as *Plutella xylostella* (Diamondback Moth), *Trichoplusia ni, Helicoverpa armigera* or *Spodoptera littoralis* as well as insect counter-defenses and adaptive mechanisms. Specialist insects have evolved mechanisms to evade the host plants' defenses and several detoxification mechanisms have been identified at both the biochemical and molecular levels. However, there is growing evidence that insects may manipulate plant defenses by means of specific spit or regurgitate factors. The development of a mechanical device (MecWorm), programmable to replicate very closely pattern, time course, and quantity of damage inflicted by different insect herbivores, has enabled us to overcome previous experimental constraints in separating physical and chemical signals originating from insects during herbivory. We will try to address what insect tissues (gland, foregut) are involved in these interactions, whether those factors are small molecules or proteins and what role in insect counter-defense these factors may play. This will facilitate our understanding whether and to which extent herbivorous insects manipulate plant defenses, a central question in studies of plant-insect co-evolution.

# Research article: The regulation of pattern in the sensory organization of the adult antenna of *Manduca sexta*

**DOI:** 10.1673/031.007.2978

**Published:** 2007-05-04

**Authors:** R.G. Vogt, M-d. Franco, J Bohbot, K. Fernandez, P. Kobres, J. Hanna, J. Poppy

**Affiliations:** ^1^ Dept. Biological Sciences, University of South Carolina, Columbia, SC 29208, U.S.A.; ^2^ currently Dept. Biology, Regis University, Denver, CO 80221, U.S.A; ^3^ currently Dept. Biological Sciences, Vanderbilt University, Nashville, TN 37232, U.S.A. vogt@biol.sc.edu

We are using the adult antenna of *Manduca sexta* to study the early developmental mechanisms influencing the phenotypes of olfactory sensilla. The adult antenna of *Manduca sexta* is a highly patterned appendage. During metamorphosis, the small larval antennae, each with about 20 olfactory receptor neurons (ORNs), are replaced by a pair of structurally complex adult antenna each with about 250,000 olfactory receptor neurons distributed among some 100,000 olfactory sensilla. These sensilla are distributed among about 80 nearly identical segment-like annuli. Each annulus divided into two regions: a sensory region containing the olfactory sensilla; and a largely non-sensory region containing scales and a very small number of sensory structures. In male antennae, each sensory region is further divided into a peripheral domain containing the sex-pheromone specific sensilla trichodea surrounding a central domain containing several classes of intermixed sensilla responsive to plant volatiles, including short sensilla trichodea, two morphologically distinct classes of sensilla basiconica as well as sensilla coeloconica (e.g. Lee K, Strausfeld NJ. 1990. *J. Neurocytology* 19: 519–538). The numbers of sensilla in the pheromone and plant-volatile sensitive domains are equivalent. The sensory region of each female annulus is more homogeneous, appearing to have only a single domain of intermingling sensilla types but lacking the pheromone-specific long sensilla trichodea (e.g. Shields VDC, Hildebrand JG. 1999. *Canadian Journal of Zoology* 77, 302–313). Emerging data support views that distinct classes of sensilla process different classes of odorant (e.g. Hansson BS et al., 2003. *Journal of Comparative Physiology A* 189: 301–308; Yao CA, Ignell R, Carlson JR. 2005. *Journal of Neuroscience* 25: 8359–8367). Olfactory sensilla might be considered small olfactory organs, comprised of neurons and support cells. These different cells may derive from a single sensory organ precursor (SOP) through asymmetric cell divisions. Sanes JR and Hildebrand JG, 1976 (*Developmental Biology* 51: 300–319) demonstrated that the mitotic activity producing these cells occurred during an approximately 24–65 hr period after pupation (a.p.). We have observed that mitotic activity initiates along the proximal and distal border of each annulus, beginning around 24 hr a.p., expanding towards the mid-annular region over the next 48 hrs a.p. Mitotic activity emerges from zones of small cells lining the proximal and distal annular boarders; these zones are already present at pupation. The spatial patterns share a strong resemblance to the distribution of olfactory sensilla on the mature antenna. The activities are ecdysteroid sensitive, and may be temporally organized in such a manner as to accommodate developmental arrest in anticipation of diapause. We have characterized the expression of a number of transcription factors, especially those of the Distal-less related pathways, suggesting the use of this basic patterning pathway in the organization of at least the lepidopteran antenna.

# Research article: Functions and uses of polydnavirus genes

**DOI:** 10.1673/031.007.2979

**Published:** 2007-05-04

**Authors:** Bruce A. Webb, Torrence A. Gill, Angelika Fath-Goodin, Jeremy Kroemer

**Affiliations:** Department of Entomology, University of Kentucky Lexington, Kentucky 40546–0091, U.S.A. bawebb@pop.uky.edu

Polydnaviruses are an unusual group of viruses that are obligate symbionts of some parasitic wasps. The genome sequence of a number of polydnavirus isolates has been determined and some functional studies of their encoded genes have begun. Polydnaviruses have been studied, in part because they have a variety of important pathogenic effects on their parasitized lepidopteran hosts. Notably, these pathogens inhibit growth and development of lepidopteran larvae and suppress immune responses of these insects. We have investigated several ichnovirus gene families from the *Campoletis sonorensis* ichnovirus (CsIV) and associated specific gene families with some of the physiological effects observed in virus infected insects. Furthermore, we have gone on to investigate the utility of members of these gene families in selected biotechnological applications. The CsIV gene family that has been associated with disruption of host growth and development is know and the cys-motif gene family based on the presence of a cysteine-rich domain in the encoded proteins. When expressed from recombinant baculoviruses or transgenic plants, several members of the cys-motif gene family inhibit host growth and development. Interestingly, we have also observed an increase in mortality in these insects and associated this mortality with effects of secondary pathogens. One polydnavirus gene family, the viral ankyrin gene family, is thought to be associated with inhibition of immune responses based upon its similarity to a known inhibitor of immune responses, the ankyrin genes. This gene family is present in all sequenced polydnavirus genomes and members of the viral gene family from the *Microplitis demolitor* polydnavirus are known to interact with members of the NF-χB signaling pathways in *in* *vitro* assays (Thoetkiattikul et al., 2005). In CsIV, the localization of viral ankyrin proteins changes markedly in transfected cells in response to immune challenge or virus infection. This studies also suggest a role for these proteins in immune suppression although we have not yet demonstrated direct binding to members of the NF-χB signaling pathway. However, we have shown that the CsIV viral ankyrin proteins alter the course of baculovirus infection by delaying death and lysis of baculovirus-infected cells. This phenomenon has enabled development of cell lines and recombinant baculoviruses that enhance expression of heterologous recombinant proteins relative to conventional systems. Taken together these studies indicate that the sequenced polydnavirus genomes have identified viral genes and gene families that may prove useful not only for investigation of functional and mechanistic studies of polydnaviruses and their host insects but also in diverse biotechnological applications. Thoetkiattikul H, Beck M and Strand MR (2005) PNAS USA 102:11426–11431.

# Research article: Genomic and proteomic analysis of innate immunity in the greater wax moth *Galleria melonella*

**DOI:** 10.1673/031.007.2980

**Published:** 2007-05-04

**Authors:** M. Wedde, B. Altincicek, A. Vilcinskas

**Affiliations:** Institute of Phytopathology and Applied Zoology, Justus-Liebig-University of Giessen, Heinrich-Buff-Ring 26–32, D-35392 Giessen, Germany. Andreas.Vilcinskas@agrar.uni-giessen.de

Systematic identification and characterization of immune-related genes and proteins in Lepidoptera was performed using larvae of the greater wax moth *Galleria mellonella* as a model. To analyse their immune-related transcriptom we used the suppression subtractive hybridization, a PCR-based method for cDNA subtraction, to screen for mRNAs that are differentially expressed in response to injected bacterial lipopolysaccaride (LPS). In addition, we subjected cell-free hemolymph samples from untreated and immunized larvae to comparative proteomic analysis. Using 2D-gelectrophoresis for separation and mass spectrometry or Edmann-sequencing for characterization of new or enhanced spots we identified numerous proteins which are synthesized and released within the hemolymph in response to injected elicitors of innate immune responses. Combined transcriptomic and proteomic analysis of innate immunity in *G. mellonella* elucidates similarities and differences when compared with the well-known *Drosophila* immune system. Lepidoptera and Diptera share some evolutionary conserved immune-related molecules such as particular antimicrobial peptides or molecules contributing to recognition of infectious non-self (e.g. peptidoglycan recognition proteins), immune-related signaling (c-Rel-like molecules) or melanization (e.g. prophenoloxidase). Among the immune-related effector molecules from *G. mellonella*, which have been purified from hemolymph of immunized larvae and/or cloned and heterologously expressed to enable further molecular characterization, we discovered a novel antifungal peptide named gallerimycin and the first specific inhibitor of microbial metalloproteinases reported from animals. This insect metalloproteinase inhibitor (IMPI) represents a potent novel inhibitor of thermolysin-like virulence factors produced by human pathogenic bacteria such as *Listeria, Clostridium, Pseudomonas* and *Vibrio*. Therefore, we presently investigate the potential of the IMPI as a template for the rational design of second generation antibiotics. Gallerimycin may represent the prototype of a novel group of insect defensins with potential in modern plant protection strategies because its transgenic expression in tobacco was recently demonstrated to confer resistance against fungal phytopathogens. Interestingly, we discovered a novel feature of insect innate immunity in *G. mellonella*: The ability to sense and to inactivate metalloproteinases associated with invading microbial pathogens, e.g. thermolysin. The presence of such non-regulated metalloproteinases in the hemolymph causes hydrolysis of proteins and subsequent generation of peptidic fragments (smaller than 3 kDa) among which particular collagen-IV fragments have been identified as potent elicitors of innate immune responses. Proteomic analysis of hemolymph samples from larvae preinjected either with LPS or with hemolymph protein fragments confirms that both induce a similar spectrum of immune-related proteins. These observations lend some credit to our hypothesis that microbial pattern recognition in Lepidoterans includes the sensing of microbial metalloproteinases via the products of their non-regulated activity which in turn induce expression of the IMPI along with other antimicrobial peptides to stop excessive degradation of host proteins during infection.

# Research article: A genomic approach to studying pyrethroid resistance in the cotton bollworm, *Helicoverpa armigera*

**DOI:** 10.1673/031.007.2981

**Published:** 2007-05-04

**Authors:** Choon Wei Wee, Charles Robin, David G Heckel

**Affiliations:** ^1^ Max Planck Institute for Chemical Ecology, Beutenberg Campus, Hans-Knoell-Str. 8, D-07745 Jena, GERMANY; ^2^ ARC Special Research Centre for Environmental Stress and Adaptation Research (CESAR), Department of Genetics, The University of Melbourne, Victoria, 3010, Australia. weechoonwei@hotmail.com

*Helicoverpa armigera* is a polyphagous lepidopteran that causes severe agricultural losses globally and readily develops resistance to insecticides. Previous research has shown that metabolic based resistance is the major cause of pyrethroid resistance in Australia, and both cytochrome P450 monooxygenases and carboxylesterases have been implicated. The AN02 strain of *H. armigera* exhibits 50 fold resistance to fenvalerate in the larval and adult stages. Resistance in AN02 can be almost completely synergised by a P450 inhibitor, piperonyl butoxide. Two techniques, cDNA-AFLP and cDNA microarrays were used to identify genes differentially expressed between fenvalerate resistant and susceptible backcross progeny from an AN02 heterozygous resistant female and susceptible male. A novel cytochrome P450 in a new subfamily was found to be constitutively overexpressed in resistant individuals, and was mapped to within 1 cM of the resistance locus *RFen1*. Sequence analysis reveals no consistent polymorphisms in the CDS associated with resistance. Two other previously-identified P450s were also over-expressed in resistant individuals, as were two glutathione S-transferases and one carboxylesterase. Mapping of these detoxicative genes to other linkage groups suggested evidence of their *trans*-regulation by a factor on the *RFen1* linkage group. Topical application of esfenvalerate on larvae of another strain (Toowoomba) results in tissue-specific induction of these detoxicative genes as well.

# Research article: A phylogenomic backbone for lepidopteran model systems

**DOI:** 10.1673/031.007.2982

**Published:** 2007-05-04

**Authors:** Christopher W. Wheat, Conrad Labandeira, P. Andolfatto, P. Beldade, Q. Feng, D. Heckel, C. Jiggins, R. Simpson, Heiko Vogel

**Affiliations:** ^1^ Pennsylvania State University, University Park, PA, USA; University of Heslinki, Helsinki, Finland, cww10@psu.edu; ^2^ National Museum of Natural History, Washington, DC 20013–7012; ^3^ University of California, San Diego, California, USA; ^4^ Leiden University, Leiden, The Netherlands; ^5^ Natural Resource Canada, Ontario, Canada; ^6,9^ Max Planck Institute for Chemical Ecology, Jena, Germany; ^7^ University of Cambridge, Cambridge, UK; ^8^ HortResearch, Palmerston North, New Zealand.

Expressed sequence tab (EST) libraries have been made for diverse lepidopteran taxa to provide genomic insight for myriad research projects ranging from wing pattern development to detoxification mechanisms of the larval gut. Here we utilize these EST resources to provide a suite of genes for reconstruction of the phylogenetic relationships among these taxa, which are all located within the Dirysia. Currently, there is little to no phylogenetic resolution above the family level for the Ditrysia, with morphilogial insights resulting in large polytomies, and molecular sources providing little to no resolution at this evolutionary depth. The shared ribosomal proteins for phylogenetic reconstruction were searched from the EST libraries of the following species: *Bicyclus anynana* (Papilionoidea), *Heliconius erato*/*melpomine* (Papilionoidea), *Pieris rapae* (Papilionoidea), *Papilio* sp. (Papilionoidea), *Manduca sexta* (Bombycoidea), *Bombyx mori* (Bombycoidea), *Helicoverpa armigera* (Noctuoidea), *Spodoptera frugiperda* (Noctuoidea), *Trichoplusia ni* (Noctuoidea), *Epiphyas postvittana* (Tortricoidea), *Choristoneura fumiferana* (Tortricoidea), *Plutella xylostella* (Yponomeutoidea), *Hydropsyche angustipennis* (Trichoptera). We show that 10 ribosomal proteins in combination with cytochrome oxidase and elongation factor 1 alpha provide robust phylogenetic inference across three different methods of phylogenetic reconstruction, in close agreement with results of morphological analyses of these taxa. We also provide an analysis of the age of butterflies.

# Research article: Progress in gene manipulation in the cotton bollworm *Helicoverpa armigera*

**DOI:** 10.1673/031.007.2983

**Published:** 2007-05-04

**Authors:** A. K. Williams, P. Batterham, D. G. Heckel

**Affiliations:** ^+^ CESAR, Department of Genetics, Bio21, University of Melbourne, Australia; ^*^ Max Planck Institute for Chemical Ecology, Jena, Germany. ak.williams@unimelb.edu.au

*Helicoverpa armigera* is a major economic crop pest that is highly resistant to pesticides. We are developing systems to analyse candidate genes involved in insecticide resistance by over-expression, ectopic expression and gene knockdown, methods routinely used in *Drosophila*.

Using piggyBac vectors we have been able to transiently drive strong EGFP and DsRed expression in eggs and young larvae via several promoters and shown that the GAL4-UAS system is functional. A recently isolated bollworm mutant, *yellow eyes* could provide a useful genetic background for germline transformation. The *armigera* homologue of the *Drosophila white* eye pigment transporter was cloned, however cDNA and Southern analysis revealed that *white* and *yellow eyes* map to different chromosomes. A genetic interaction between the *yellow eyes* mutant and a *dark eyes* mutant is reminiscent of the Drosophila *scarlet*/*brown* interaction, producing a depigmented double mutant. The *armigera* homologue of *scarlet* is being cloned to determine if it is the lesion in the *yellow eyes* mutant. The *yellow eyes* mutant also exhibits reduced pigmentation in the egg and first instar larva. We have produced partial phenocopies of the egg phenotype by targeting the *white* gene with double stranded RNA interference and antisense morpholino oligonucleotides. Transient EGFP expression has also been knocked down using dsRNA to the EGFP transcript.

# Research article: Genome-wide analysis of expression profiles in silkworm using an oligonucleotide microarray

**DOI:** 10.1673/031.007.2984

**Published:** 2007-05-04

**Authors:** Qingyou Xia, Zeyang Zhou, Cheng Lu, Zhonghuai Xiang, Liang Zhang

**Affiliations:** ^1^ The Key Sericultural Laboratory of Agricultural Ministry, Southwest University, Chongqing 400716, China; ^2^ CapitalBio Corporation, Beijing 102206, China. xiaqy@swau.cq.cn

With whole genome sequencing of target organisms finished, the genome project is gradually changing from structural genome into functional genome research. Microarrays, which can measure the individual transcript level of tens of thousands of genes simultaneously, provide a means to high-through-put analysis of gene expression levels at different developmental stages or under different physiological conditions. Microarray analysis has been broadly applied in the study of functional genomes, such as those of the human, mouse, fruit fly, rice and nematode etc. The silkworm, *Bombyx mori* is not only one of the most important economic insects, but also a model lepidopteran insect for genetic, molecular and genomic studies. The bioinformatical analysis of silkworm WGS data has provided a great deal of information of genes involved in several important traits such as sex differentiation, immune response, silk formation, and metamorphism etc. Therefore, it is possible to approach gene function on a genomic scale and identify candidate gene groups associated with specific developmental pathways as well. Undoubtedly, microarray is an excellent method that is suitable for achieving the goal just mentioned above. We designed a genome-wide oligonucleotide microarray for the silkworm with the purpose of monitoring gene expression profiles of different tissues and different stages on a global scale, developing experimental evidence for functional assignments and identifying gene clusters related to important economic traits of silkworm. The results of this microarray analysis will also be helpful in interpreting the network of gene regulation and creating new genetic materials. Here, we briefly report the results about the production and application of the silkworm oligonucleotide microarray. We designed and produced a 69-mer oligonucleotide microarray that represents approximately 23, 000 known and predicted mRNAs based on the silkworm WGS, ESTs and other sequences in Genbank. This is the first genome-wide microarray for the silkworm. Considering the important regulative function of miRNA, we produced the first miRNA microarray which contains 106 predicted miRNAs in the silkworm. The expression profiles of nine tissues of silkworm in the third day of the fifth instar have been completed. The raw data were normalized by Lowess and clustered by cluster 3.0. The results revealed almost no significant expression difference between males and females except for the gonads. However, it showed remarkable differences between the expressional patterns of testis and ovary. Gene ontology classification also indicated that the genes involved in sex differentiation and propagation have a higher expression in testis. The analysis of tissue-specific expression showed that genes encoding sperm tail associated protein, spermatogenesis apoptosis-related protein and malate dehydrogenase were specifically expressed in the testis and in formation of sperm. Large numbers of genes related to storage proteins such as the 30KD proteins, SP1 and SP2 were highly expressed in fat body. A cluster of genes had similar expression patterns among the head and the epidermis, suggesting that there is some common composition between them. In addition, genes related to the formation of the sensory system were highly expressed in the head. Genes involved in digestion and absorption were specifically expressed in the midgut. Further research showed that a large number of genes with a tissue-specific expression patterns have no exact function annotation. These results are very helpful for discovering new genes and investigating their molecular roles. Additionally, the preliminary results of the miRNA microarray were exciting, which showed that some miRNAs were detected in the head and the gonad of silkworm in the third day of the fifth instar. The most interesting discovery was that several miRNA displayed obvious expression difference between testis and ovary, which suggested that miRNAs may play a very important role in sex differentiation of silkworm. These results may provide important insights for understanding how miRNA is involved in the regulation of silkworm development.

# Research article: Silkworm genome analysis: Integration of SNPs linkage map, physical map and sequence scaffold using BAC clone information as a staple

**DOI:** 10.1673/031.007.2985

**Published:** 2007-05-04

**Authors:** Kimiko Yamamoto, Junko Narukawa, Keiko Kadono-Okuda, Junko Nohata, Yoshitaka Suetsugu, Motoe Sasanuma, Shun-ichi Sasanuma, Hiroshi Minami, Michihiko Shimomura, Kazuei Mita

**Affiliations:** ^1^ Genome Research Department, National Institute of Agrobiological Sciences, Tsukuba, Ibaraki 305–8634, Japan; ^2^ Mitsubishi Space Software Co. Ltd., Tsukuba, Ibaraki 305–0032, Japan. kiya@nias.affrc.go.jp

Recently, there is an increasing importance of silkworm as genetic and bio-material resources that have potentials to provide new applications in medical, agricultural and industrial fields. In Japan, the genetic data of the silkworm has been accumulated through the sericultural study. The silkworm is also important as a model insect for Lepidoptera, which include the most highly destructive agricultural pests. Due to these industrial and agricultural interests, the genome analysis of the silkworm is urgently required. We have been also proceeding with silkworm whole-genome sequencing (WGS), EST analyses and physical mapping (BAC-contig construction) by finger-print method, etc. The integration of the results from these analyses and presently available linkage maps based on molecular markers will play a key role in post-genome investigations. To realize the integration, the BAC clone information such as partial BAC nucleotide sequences and a BAC based molecular linkage map are strongly required. For this purpose, we had performed the end sequence analysis of ca. 80,000 BAC clones from three libraries constructed with *Eco*RI, *Hindlll* and *Bam*HI. Then, we have developed a SNP linkage map for the silkworm based on the polymorphisms in the obtained BAC end sequences between strains p50T. In silkworm, although several silkworm linkage maps based on molecular markers including RFLP, RAPD, and AFLP are presently available, no linkage map composed of single-nucleotide polymorphisms (SNPs) has been constructed so far. The SNPs in the BAC end regions were detected by the sequencing of the 5,760 randomly selected end sequence regions of the parent strains and F1. Then, using 190 segregants from a backcross of a p50T female × an F1 (p50T × C108T) male, we analyzed segregation patterns of SNPs between p50T and C108. As a result, we constructed a SNP linkage map composed of more than 1500 SNP markers spanning 1387 cM in total length distributed over 28 linkage groups. We assigned these linkage groups to previously reported ones using morphological mutants except for chromosome × (Z), which was assigned based on sex-specific segregation patterns of the SNP markers. The developed SNP linkage map will play a key role as a staple to integrate WGS, EST and BAC-contig data, since the common BAC clones were used for BAC contig construction and EST analyses. At present, we are proceeding with the survey of EST candidates contained in the mapped BAC clones using HDR filter and BLAST search, and the localization of significant numbers of ESTs on the linkage map were assigned by exploiting the BAC markers. Now we are also constructing an integrated silkworm genome database named KAIKObase. The SNP map, together with the KAIKObase, will be a powerful tool for investigating silkworm genome properties, mutation mapping, and map-based cloning of genes of industrial and agricultural interest. This study was supported by Insect Genome Project, MAFF and fund of Promotion of Basic Research Activities for Innovative Biosciences from BRAIN.

# Research article: Molecular evolutional comparison among some populations of *B. mandarina* distributing in China, Korea, and Japan while as comparing with some strains of *B. mori* by analyzing *og* gene, mitochondrial *cox1* gene, *Alp* genes, and *mariner*-like-elements

**DOI:** 10.1673/031.007.2986

**Published:** 2007-05-04

**Authors:** K. Yukuhiro, M. Itoh, Y. Banno, N. Kômoto, E. Kosegawa, M. Hirokawa, K. Tatematsu, M. Nishimura, H. Maekawa, Y. Kawanishi, Y. Nakajima

**Affiliations:** ^1^ Insect Molecular Evolution Laboratory, Insect Evolution and Genetics, Department, National Institute of Agrobiological Sciences, Tsukuba, 305–8634, Japan; ^2^ Dept. Applied Biology, Kyoto Institute of Technology, Kyoto, 606–85853, Japan; ^3^ Institute of Genetic Resources, Graduate School of Bioresource and Bioenvironmental Sciences, Kyushu University, Fukuoka, 812–8581, Japan; ^4^ Insect Genetics Laboratory, Insect Evolution and Genetics, Department, National Institute of Agrobiological Sciences, Kobuchizawa, 408–0044, Japan; ^5^ Division of Radiological Protection and Biology, National Institute of Infectious Diseases, Tokyo 162–8640, Japan; ^6^ Division of Functional Genomics, Center of Molecular Biosciences (COMB), University of the Ryukyus, Okinawa, 903–0213, Japan. yumik028@comb.u-ryukyu.ac.jp

*Bombyx* mandarina is thought to have the same ancestor of domesticated silk moth *Bombyx mori*. From the molecular evolutional calculation, it has estimated that they branched off each other from the common ancestor about several million years ago. *B. mandarina* distribute in mainly East Asian region according to the difference of the chromosome numbers. That to say, *B. mandarina* inhabiting in China are 28, on the other hand, *B. mandarina* in Korea and also Japan are 27. Moreover, we can sometimes see the hybrid specimens between 27 and 28 in Korea. From the sequence comparison about some DNA marker gene or flanking region between them, genetically, *B. mori* might be close to the population of *B. mandarina* inhabiting in China. So how and when both species have imported into Japan may be the different way and route. From the viewpoint of comparative genome, we are now studying the molecular level of polymorphism in *B. mori* and also progressing to compare some genes or DNA marker regions between *B. mori* and *B. mandarina*, or among some populations of *B. mandarina* distributing in different area of East Asian regions, We preset the recent analyzing or comparing results of them with as following regions. First we studied the molecular level of polymorphism in *B. mori* molybdenum cofactor sulfurase gene (*og*) whose mutants cause translucent skin because of lack or deficiency of accumulation of uric acid under the skin in *B. mori*, We found five haplogroup sin 33 regional races of *B. mori*. Furthermore, we analyzed the *og* gene of Japanese *B. mandarina* samples and compare level of genetic diversity and mutational profile between two species. In addition, we conducted an analysis of mitochondrial *cox1* gene of *B. mori* and found low level of sequence diversity relative to that of the *og* gene. Alkaline phosphatase (*Alp*) genes are tandemly duplicated in *B. mori*. The *Alp* genes (*Alp*-*m* and *Alp*-s) share about 60% identity in the nucleotide sequences, but structure of the intervening region is highly polymorphic with large in/del from strain to strain. In this study we found that the *Alp* genes were shown to be duplicated in *B. mandarina*, similarly as in *B. mori*, in all four gemones from three local wild populations. The structure of the intervening region in *B. mandarina* suggested that the most recent common ancester of the current silk worm strains and wild *B. mandarina* existed in China. We analyzed the full length of MLEs from the genome of *B. mandarina* and inserted sequences into them which have been isolated by the PCR method using the Inverted Terminal Repeat of *Hyalophora cecropia marmer*-like-element. The features of the Cecropia-ITR-MLE sequences from *B. mandarina* might relate with the geographical distribution region of them. Most of MLE sequences from the specimens of *B. mandarina* in the main island of Japan composed one unique group (Japanese type); on the other hand, the sequences from the Chinese and Korean specimens settled down in another group (Continental type). Sequences of *B. mandarina* inhabiting in Japan close to the Asian Continent spread over these two distinctive groups. We will also show the features of inserted sequences into the Cecropia-ITR-MLE from *B. mandarina* in Japan.

# Research article: DNA microarray analysis of gene expression patterns during molting in the spruce budworm, *Choristoneura fumiferana*

**DOI:** 10.1673/031.007.2987

**Published:** 2007-05-04

**Authors:** Dayu Zhang, Tim Ladd, Sichun Zheng, Lan Li, Deborah Buhlers, Arthur Retnakaran, Peter J, Krell, Basil M. Arif, Daniel Doucet, Qili Feng

**Affiliations:** ^1^ Great Lakes Forestry Centre, Canadian Forest Service, Natural Resources Canada, 1219 Queen Street East, Sault Ste. Marie, Ontario, Canada, P6A 2E5; ^2^ Department of Microbiology, University of Guelph, Guelph, Ontario, Canada, N1G 2W1; ^*^ Current address: College of Life Sciences, South China Normal University, Guangzhou, China 510631. qlfeng@scnu.edu.cn

Insect molting process is initiated by 20-hydroxyecdysone (20E) and regulated by a cascade of gene expression. To analyze gene expression patterns during the larval molts, a cDNA-based microarray was constructed. The array contained 2,600 unigenes represented by 13,000 expressed sequence tags (ESTs) from three cDNA libraries. Gene expression patterns in the larvae molting from the 5^th^ to 6^th^ instar stage were analyzed using this array. Out of 2,600 unigenes 332 genes showed at least three-folds change in expression levels either before or after the reference stage, head capsule slippage. These genes were clustered into four major groups based on their expression patterns and responses to 20E. The predicted functions of these genes are mainly involved in the biological events such as cuticle synthesis and degradation, chitin synthesis and degradation, cuticle pigmentation, myogenesis, transcription and translation, digestion metabolism, cell proliferation and death. This project was supported by a Canadian Biotechnology Strategy Fund and by Genome Canada through the Ontario Genomics Institute.
